# Apical bulkheads accumulate as adaptive response to impaired bile flow in liver disease

**DOI:** 10.15252/embr.202357181

**Published:** 2023-07-31

**Authors:** Carlotta Mayer, Sophie Nehring, Michael Kücken, Urska Repnik, Sarah Seifert, Aleksandra Sljukic, Julien Delpierre, Hernán Morales‐Navarrete, Sebastian Hinz, Mario Brosch, Brian Chung, Tom Karlsen, Meritxell Huch, Yannis Kalaidzidis, Lutz Brusch, Jochen Hampe, Clemens Schafmayer, Marino Zerial

**Affiliations:** ^1^ Max Planck Institute of Molecular Cell Biology and Genetics Dresden Germany; ^2^ Department of Medicine I, Gastroenterology and Hepatology University Hospital Carl‐Gustav‐Carus, Technische Universität Dresden (TU Dresden) Dresden Germany; ^3^ Center for Information Services and High‐Performance Computing Technische Universität Dresden Dresden Germany; ^4^ Central Microscopy, Department of Biology Christian‐Albrechts‐Universtät zu Kiel (CAU) Kiel Germany; ^5^ Department of General Surgery University Hospital Rostock Rostock Germany; ^6^ Center for Regenerative Therapies Dresden (CRTD) Technische Universität Dresden (TU Dresden) Dresden Germany; ^7^ Department of Transplantation Medicine, Clinic of Surgery, Inflammatory Medicine and Transplantation, Norwegian PSC Research Center Oslo University Hospital Rikshospitalet Oslo Norway; ^8^ Research Institute of Internal Medicine, Clinic of Surgery, Inflammatory Diseases and Transplantation Oslo University Hospital and University of Oslo Oslo Norway

**Keywords:** Apical bulkheads, Bile canaliculi, Hepatocyte rosettes, Hepatocytes, Primary sclerosing cholangitis, Digestive System, Molecular Biology of Disease

## Abstract

Hepatocytes form bile canaliculi that dynamically respond to the signalling activity of bile acids and bile flow. Little is known about their responses to intraluminal pressure. During embryonic development, hepatocytes assemble apical bulkheads that increase the canalicular resistance to intraluminal pressure. Here, we investigate whether they also protect bile canaliculi against elevated pressure upon impaired bile flow in adult liver. Apical bulkheads accumulate upon bile flow obstruction in mouse models and patients with primary sclerosing cholangitis (PSC). Their loss under these conditions leads to abnormally dilated canaliculi, resembling liver cell rosettes described in other hepatic diseases. 3D reconstruction reveals that these structures are sections of cysts and tubes formed by hepatocytes. Mathematical modelling establishes that they positively correlate with canalicular pressure and occur in early PSC stages. Using primary hepatocytes and 3D organoids, we demonstrate that excessive canalicular pressure causes the loss of apical bulkheads and formation of rosettes. Our results suggest that apical bulkheads are a protective mechanism of hepatocytes against impaired bile flow, highlighting the role of canalicular pressure in liver diseases.

## Graphical abstract

Hepatocytic apical bulkheads protect bile canaliculi against dilation and hepatocyte rosette formation upon elevated canalicular pressure. These findings highlight the significance of canalicular pressure in liver diseases and the protective role of apical bulkheads in the adult liver.

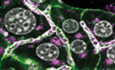

## Introduction

Bile is essential for energy homeostasis and detoxification. It is produced by hepatocytes in the liver and is secreted through the apical membrane into the biliary tree. The most proximal part of the biliary tree is the bile canaliculi network formed by hepatocytes and connected to distant bile ducts lined by bile duct cells. Even though hepatocytes and bile duct cells are both epithelial cells that arise from a common progenitor in embryonic development (Müsch, 2018), their cellular architectures and lumen morphologies are strikingly different: Bile canaliculi are narrow lumina of 0.5–1 μm in diameter lined by the apical membrane of just two adjacent hepatocytes. Each hepatocyte shares the apical surface with many neighbouring hepatocytes, collectively forming a 3D network of bile canaliculi. In contrast, bile ducts are typical epithelial tubes of > 5 μm in diameter formed by multiple bile duct cells arranged around the same central lumen and enveloped by a basal membrane (Steiner & Carruthers, 1961; Motta & Fumagalli, 1975; Benedetti *et al*, 1996). Due to these arrangements, bile ducts have a characteristic cross‐sectional morphology of rosette‐like structures. These differences in lumen morphology between bile canaliculi and bile ducts underlie fundamental changes in cell polarity determined by distinct cellular trafficking and organization (Zeigerer *et al*, 2012; Gissen & Arias, 2015). Hence, liver function depends on the proper polarization of the two cell types.

Different liver diseases are characterized by morphological alterations of the biliary tree, including disruption of bile canaliculi connectivity (Segovia‐Miranda *et al*, 2019) and compensatory proliferation of reactive bile ducts. The latter process is called ductular reaction, characterized by bile duct hyperplasia (Sato *et al*, 2019). These reactive bile ducts can be formed by an intermediate hepatobiliary cell type that is thought to aid liver regeneration (Song *et al*, 1998; Roskams *et al*, 2004; Sato *et al*, 2019). Morphologically, they are characterized by a rosette‐like arrangement of cells similar to bile duct cells that are also directly connected to pre‐existing bile ducts (Lenzi *et al*, 1992). Another tissue alteration of the biliary tree in liver disease are so‐called liver cell rosettes. These are characterized by substantial canalicular dilation concomitant with a rearrangement of hepatocytes into tubular structures, also resembling bile ducts architecture (Butron Vila *et al*, 1984; Desmet, 1986; Nagore *et al*, 1989). The molecular mechanisms driving the emergence of liver cell rosettes and their role in the pathogenesis of liver diseases—including their putative contribution to the ductular reaction—are currently unknown.

The morphological alterations of liver tissue in disease can reflect alterations of bile flow. Multiple processes have been uncovered in the adaptive response of the liver to changes in bile flow and accumulation of bile acids. Most studied are the direct effects of bile acids on specific nuclear receptors (e.g. FXR) that modulate bile acid synthesis and transport (Sinal *et al*, 2000; Wagner *et al*, 2009; Fuchs & Trauner, 2022). Much less understood are the mechanical consequences of altered bile flow for liver tissue integrity. Bile canaliculi are dynamic tubules formed by the apical surface of hepatocytes which can expand or constrict, exploiting actomyosin‐mediated contractility in response to changes in bile acids homeostasis (Gupta *et al*, 2017; Meyer *et al*, 2020). Altered bile acid homeostasis impairs bile flow leading to the accumulation of bile inside the biliary tree, as, e.g. in obstructive cholestasis (Jansen *et al*, 2017; Chiang & Ferrell, 2018). Since bile acids act as osmolytes, such accumulation increases biliary pressure and hence alters the mechanical properties of the tissue. It remains unclear how these physical changes alter tissue structure in detail and whether this can affect hepatocyte fate and function.

By investigating the mechanisms underlying bile canaliculi formation during embryonic development, the existence of previously unrecognized sub‐cellular structures that connect the apical surfaces of two adjacent hepatocytes was described (Belicova *et al*, 2021). These apical connections appear as a characteristic pattern of F‐actin‐positive stripes. Due to their periodic pattern resembling the bulkheads of boats, they were termed apical bulkheads. Even though apical bulkheads are shield‐like protrusions traversing bile canaliculi, they do not close the lumen completely, hence, allowing continuous bile flow. Apical bulkheads are formed during embryogenesis, when hepatoblasts differentiate into hepatocytes but not during differentiation into bile duct cells. They are not an epiphenomenon but are required for the elongation of bile canaliculi. Genetic manipulations that cause the loss of apical bulkheads, such as silencing of the small GTPase Rab35 or Cdc42, also cause alterations in hepatocyte polarity and formation of epithelial cysts and tubules structurally similar to bile ducts (Belicova *et al*, 2021). Cell biological and biophysical analysis combined with mechanical modelling revealed that apical bulkheads are load‐bearing mechanical elements that stabilize the growing bile canaliculi in the embryonic liver and can sustain double the luminal pressure that these thin tubes can normally hold (Bebelman *et al*, 2023). Although apical bulkheads were identified in cultured hepatoblasts and developing liver, they are also present in adult liver tissue, suggesting that they may play a role in liver physiology (Belicova *et al*, 2021). These structures may correspond to the evaginations/outpouchings of the canalicular membrane described by Boyer and colleagues after bile acid infusion in rats (Nemchausky *et al*, 1977). Due to the complex morphology and small size, apical bulkheads are difficult to visualize, especially with light microscopy. Hence, their frequency, dimensions and dynamics in liver tissue are unknown at present.

Given their protective role during embryogenesis, we hypothesized that apical bulkheads might also play a role in the adult liver to maintain the structure of the bile canaliculi upon changes in bile flow and canalicular pressure. Furthermore, we wondered whether there is a relationship between the alterations in the bile canaliculi network and the appearance of liver cell rosettes upon impaired bile flow. We used high‐resolution microscopy from the ultrastructural to the network‐level, 3D reconstruction, mathematical pressure modelling and different *in vitro* and *in vivo* models to investigate the role of apical bulkheads in patients with primary sclerosing cholangitis (PSC) and the relevance of biliary pressure.

## Results

### Hepatocytes accumulate apical bulkheads in mouse models of impaired bile flow *in vivo*

**Figure 1:**
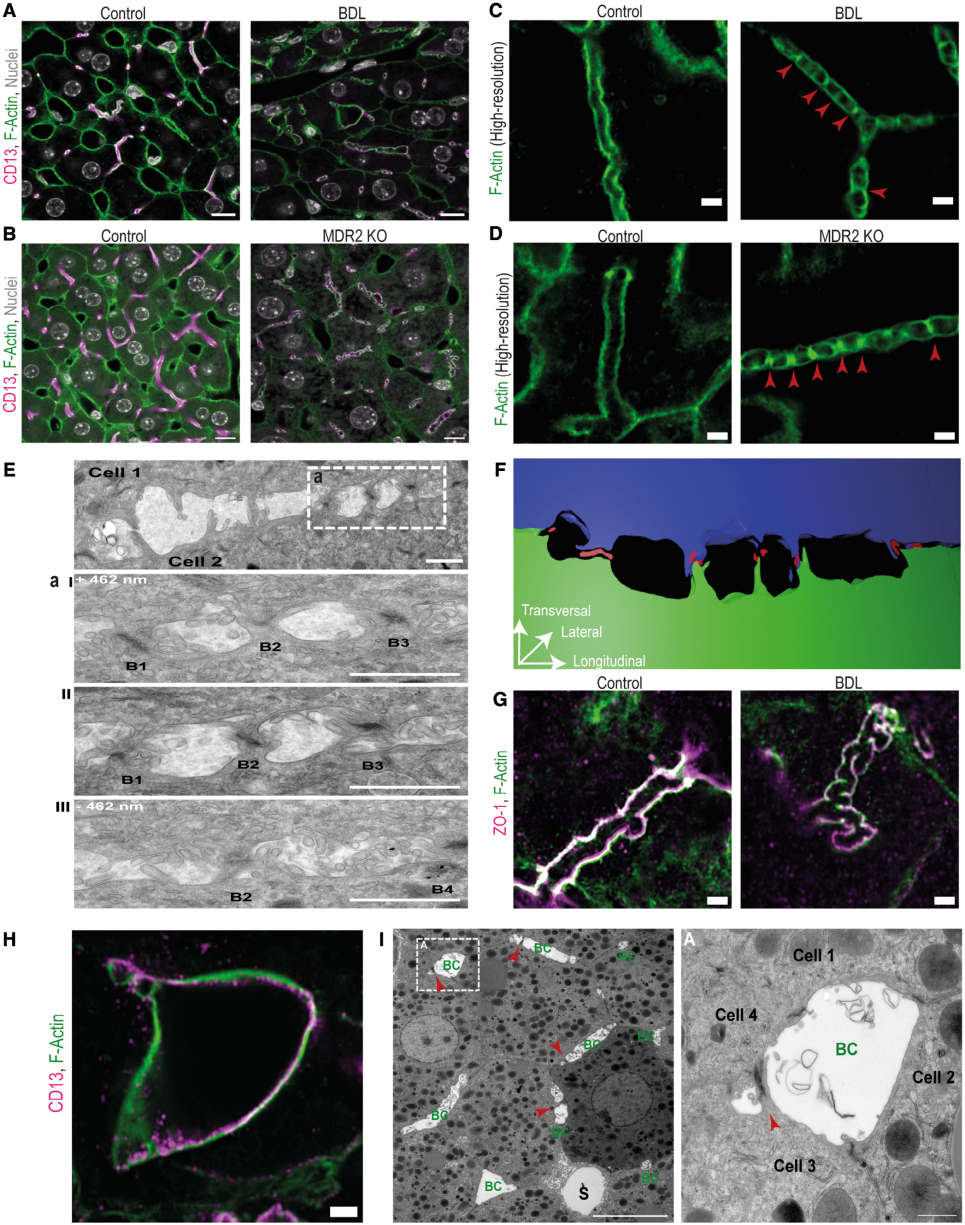
Hepatocytes accumulate apical bulkheads in mouse models of impaired bile flow *in vivo* A–DHepatocytes accumulate apical bulkheads upon bile duct ligation (BDL) and MDR2 KO. (A, B) Overview images of (A) murine liver tissue after control surgery or bile duct ligation (BDL) for 24 h (*N* = 3 biological replicates), or (B) control and MDR2 KO mice after 12 weeks (*N* = 3 biological replicates). Immunofluorescence for the apical membrane marker CD13 (magenta), F‐actin (green) and nuclei (grey). Representative images are shown. Scale bar 10 μm. (C, D) High‐resolution imaging of individual bile canaliculi in murine liver tissue (C) after control and 24 h BDL surgery, or (D) in control and MDR2 KO mice, stained for F‐actin (green). Representative images are shown. Scale bar 2 μm. Apical bulkheads are highlighted with red arrowheads.E–GApical bulkheads in the adult liver resemble those described in the embryonic liver. (E) Transmission electron microscopy (TEM) longitudinal 80‐nm‐thin serial section on liver tissue with BDL for 24 h shows the ultrastructure characteristic of apical bulkheads. Representative images are shown. Scale bar 1 μm. I–III represent consecutive sections with 460 nm distance showing four apical bulkheads (B1–B4) sealed by electron‐dense tight junctions. Scale bar 1 μm. (F) 3D reconstruction of the serial section TEM dataset in liver tissue after BDL (from 1E). The apical poles of two opposing hepatocytes are coloured in green and blue, whereas tight junctions are visualized in red. (G) High‐resolution imaging of individual bile canaliculi in liver tissue after control and BDL surgery for 24 h. Immunofluorescence of the tight junction protein ZO‐1 (magenta) and F‐actin (green) showing that in canaliculi of control livers ZO‐1 localizes only to the apical membrane (left panel). After BDL surgery, ZO‐1‐positive apical bulkheads become visible inside the lumen (right panel). Representative images are shown. Scale bar 2 μm.H, IHepatocytes form aberrantly dilated canaliculi formed by > 3 cells after BDL. (H) High‐resolution imaging of CD13 (magenta) and F‐actin (green) staining showing an individual aberrant bile canaliculus in the liver tissue after 24 h BDL. Scale bar 2 μm. (I) Transmission electron microscopy (TEM) in liver tissue after 24 h BDL. Scale bar 10 μm. Bile canaliculi (BC) can be severely dilated similar to the size of sinusoids (S) and are formed by multiple hepatocytes (see inset A, scale bar 1 μm). Apical bulkheads (marked with red arrowheads) are visible. Individual hepatocytes are part of a liver cell rosette with one part of their apical membrane while they form normal canaliculi with apical bulkheads at another pole (see inset A, cells 3 and 4). Source data are available online for this figure.

**Figure EV1:**
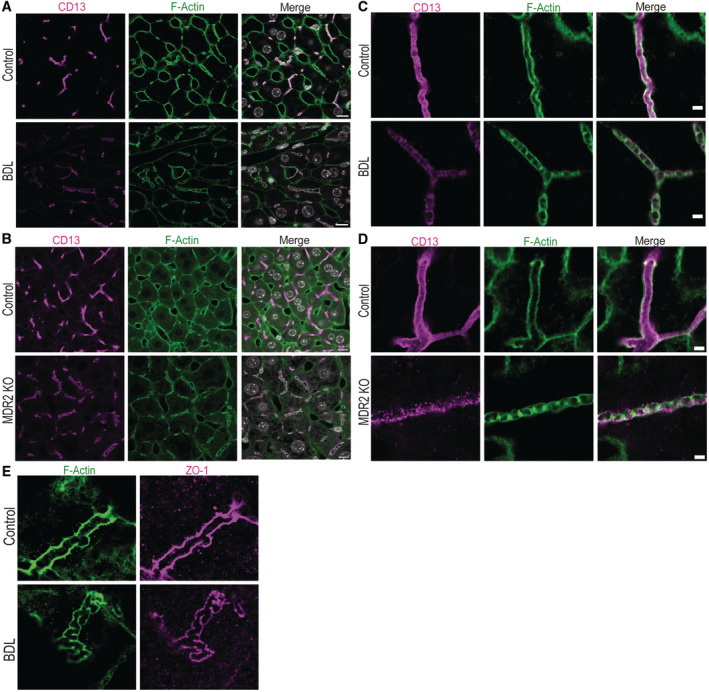
F‐Actin co‐localizes with the canalicular makers CD13 and ZO‐1 in bile canaliculi Overview images of murine liver tissue after control surgery or bile duct ligation (BDL) for 24 h (*N* = 3 biological replicates). Immunofluorescence from Fig 1A stained for CD13 (magenta), F‐actin (green) and nuclei (grey). Scale bar 10 μm.Overview images of murine liver tissue of control and MDR2 KO mice after 12 weeks (*N* = 3 biological replicates). Immunofluorescence from Fig 1C stained for CD13 (magenta), F‐actin (green) and nuclei (grey). Scale bar 10 μm.High‐resolution imaging of individual bile canaliculi in murine liver tissue after control and BDL surgery from Fig 1B. Immunofluorescence for CD13 (magenta) and F‐actin (green). Scale bar 2 μm.High‐resolution imaging of individual bile canaliculi in murine liver tissue of control and MDR2 KO mice after 12 weeks (N = 3 biological replicates) from Fig 1D. Immunofluorescence for CD13 (magenta) and F‐actin (green). Scale bar 2 μm.High‐resolution imaging of individual bile canaliculi in murine liver tissue after control and BDL surgery for 24 h from Fig 1G. Immunofluorescence for ZO‐1 (magenta) and F‐actin (green). Scale bar 2 μm.

As a first step, we aimed to investigate whether elevated canalicular bile pressure alters apical bulkheads and the bile canalicular network in the liver tissue of two murine mouse models *in vivo*. To gain first insights into the response of hepatocytes to elevated canalicular pressure, we took advantage of the mouse model of bile duct ligation (BDL) and MDR2 knockout (MDR2KO) mice, well‐established models for impaired bile flow (Fickert *et al*, 2002; Georgiev *et al*, 2008; Tag *et al*, 2015). Apical bulkheads were originally visualized by electron microscopy (EM; Belicova *et al*, 2021). By EM they are characterized as extensions of the apical plasma membrane of hepatocytes traversing the bile canaliculi sealed by electron‐dense tight junctions. Apical bulkheads were previously observed in healthy liver tissue (Belicova *et al*, 2021), but their exact abundance and extent to which they are detectable by light microscopy techniques are unknown at present. Our analysis revealed that apical bulkheads accumulate prominently after 24 h of BDL and in MDR2KO mice, visible as characteristic pattern of F‐actin‐positive membrane protrusions traversing the bile canaliculi lumen (Figs 1A–D and EV1A–D). The identity of bile canaliculi was unambiguously confirmed by co‐staining with CD13 (Figs 1A and B and EV1A–D), a marker exclusively localized to bile canaliculi (Röcken *et al*, 2005). We next set to confirm that the observed F‐actin‐positive membrane protrusions indeed correspond to the apical bulkheads described previously (Belicova *et al*, 2021). First, we verified their ultra‐structure with longitudinal 80‐nm‐thin serial sections using transmission electron microscopy (TEM) of individual bile canaliculi (Fig 1E). 3D reconstruction of the TEM serial sections showed that also upon BDL apical bulkheads do not separate the lumen into chambers allowing continuous bile flow (Fig 1F; Movie EV1). Second, we could show that the F‐actin stripes inside the bile canaliculi that appear upon BDL co‐localize with the tight junction protein ZO‐1 (Figs 1G and EV1E).

Upon BDL, but not in the control, there were few individual segments in the bile canaliculi network with hepatocytes lacking apical bulkheads and those forming aberrant, spherical bile canaliculi (Fig 1H). EM analysis showed that these bile canaliculi are not formed by hepatocyte doublets but by multiple cells and can reach sizes similar to sinusoids or bile ducts (Fig 1I). It also became apparent that a given hepatocyte can form a bile canaliculus with apical bulkheads with one neighbouring hepatocyte at one pole while forming an aberrant multicellular lumen devoid of apical bulkheads at another pole (Fig 1I). This suggests that the spherical bile canaliculi are not isolated but connected to the rest of the bile canaliculi network.

These results describe for the first time the accumulation of apical bulkheads in adult mouse livers upon different disease models characterized by impaired bile flow (BDL and MDR2KO). We confirmed the identity of the observed structures as apical bulkheads as originally described. These findings suggest (i) that hepatocytes can sense changes in pressure inside the bile canaliculi, and (ii) that they universally respond by accumulating apical bulkheads. Accumulation of apical bulkheads under different conditions is prominently visible throughout the tissue even without high‐resolution imaging. Also, it appears that the absence of apical bulkheads under these conditions is associated with hepatocytes rearranging their bile canaliculi from a tubular into spherical or cylindrical shape resembling bile duct or liver cell rosette morphology.

### Hepatocytes of PSC patients accumulate apical bulkheads and their absence correlates with the formation of aberrant liver cell rosettes.

We hypothesized that similar pathophysiological alterations as observed in the mouse models could occur in human liver diseases with impaired bile flow. To test this hypothesis, we analysed human liver tissue from patients with primary sclerosing cholangitis (PSC), a progressive liver disease with fibrosis and strictures of the bile ducts, causing bile flow obstruction and a severe cholestatic phenotype (Karlsen *et al*, 2017).

**Figure 2:**
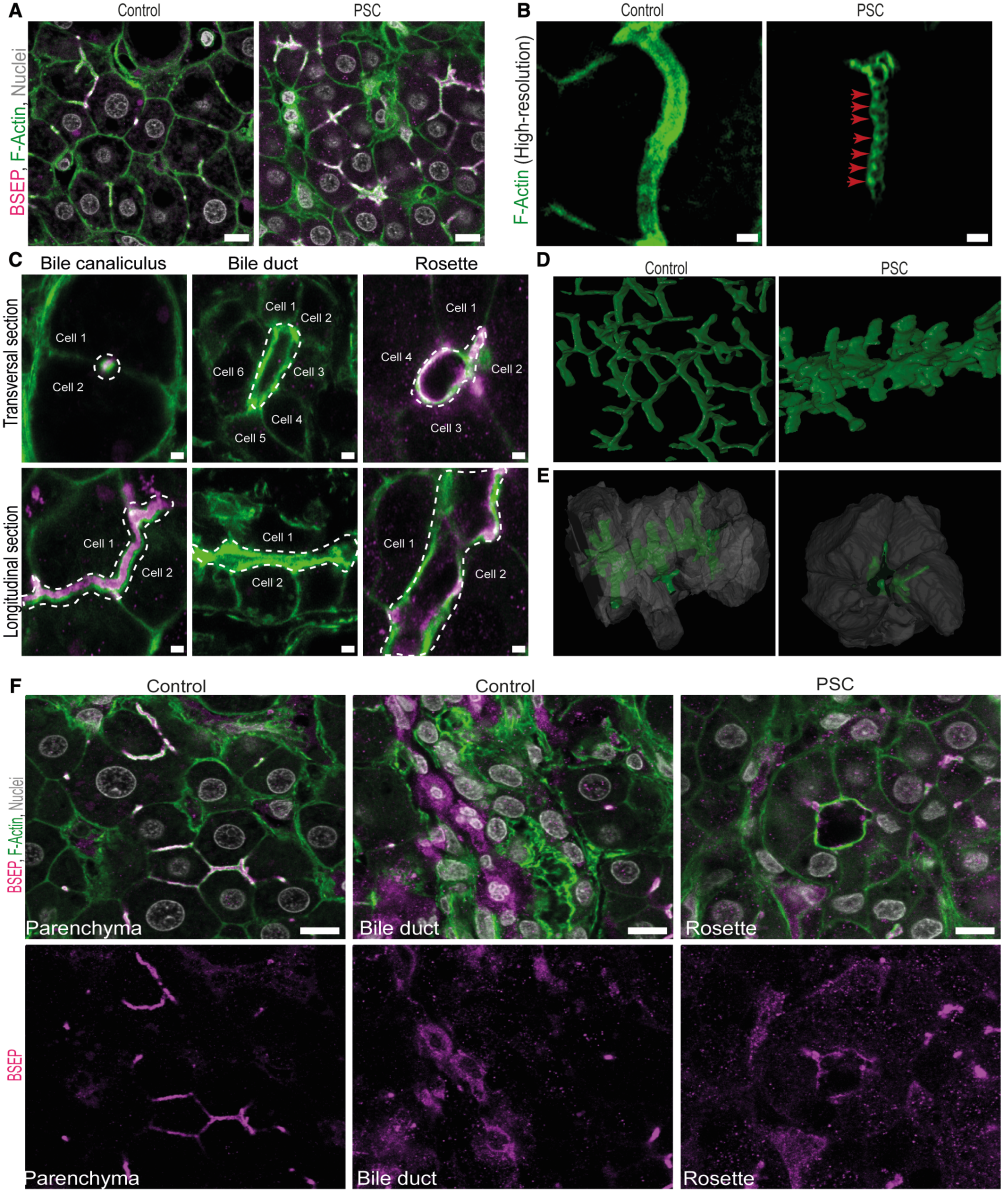
Hepatocytes of PSC patients accumulate apical bulkheads and their absence correlates with the formation of aberrant liver cell rosettes A, BHepatocytes accumulate apical bulkheads in the liver tissue of primary sclerosing cholangitis (PSC) patients (*N* = 3 control patients and *N* = 4 PSC patients). (A) Overview images in liver tissue of control and PSC patients. Immunofluoresence for the apical membrane marker BSEP (magenta), F‐actin (green) and nuclei (grey). Scale bar 10 μm. (B) High‐resolution imaging of F‐actin (green) showing individual bile canaliculi in control or PSC liver tissue. Red arrowheads mark apical bulkheads. Scale bar 2 μm.CDifferent lumen morphologies of a typical bile canaliculus (left column) and bile duct (middle column) in control patients and rosette (right column) in PSC patients. Immunofluorescence for BSEP (magenta) and F‐actin (green). Shows also differences dependent on the imaging plane, i.e. transversal (top row) or longitudinal (bottom row) cut of the lumen. White dashed lines mark the lumen. Scale bar 2 μm.D3D reconstruction of segments from the bile canaliculi network in control patients showing narrow canaliculi (left), and in PSC patients, showing that liver cell rosettes form segments of epithelial tubes connected to the BC network (right).E3D reconstruction of an epithelial tube in PSC patients showing hepatocytes (grey) surrounding an individual segment of canalicular lumen (green). On the right is a transversal cross‐section through this tube revealing the typical morphology of the liver cell rosettes.FLiver cell rosettes in PSC patients are formed by hepatocyte‐like cells (*N* = 3 control patients and *N* = 4 PSC patients). Immunofluoresence for BSEP (magenta), F‐actin (green) and nuclei (grey) in healthy parenchyma (left) and bile duct (middle) in control patients and liver cell rosettes in PSC patients (right). Scale bar 10 μm. Source data are available online for this figure.

**Figure EV2:**
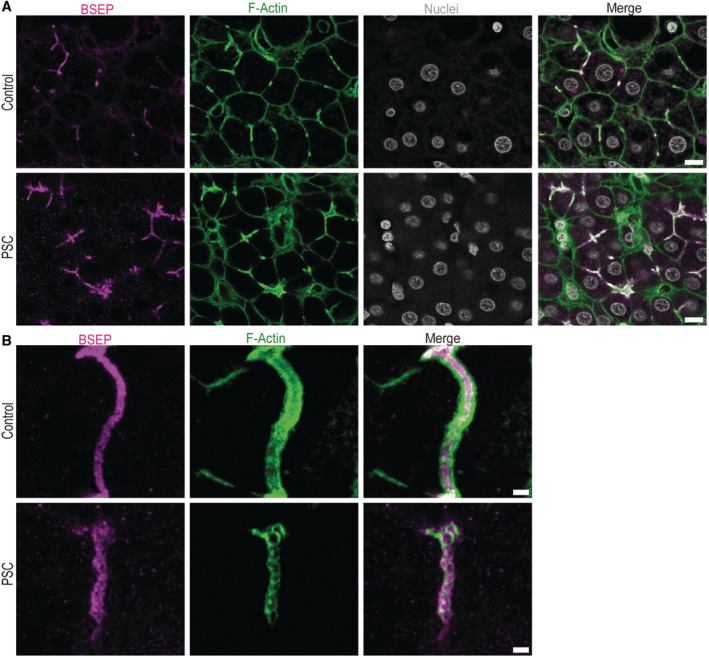
F‐Actin co‐localizes with the canalicular marker BSEP in bile canaliculi Overview images in liver tissue of human control and PSC patients from Fig 2A. Individual channels of immunofluoresence for the apical membrane marker BSEP (magenta), F‐actin (green) and nuclei (white). Scale bar 10 μm.High‐resolution imaging of individual bile canaliculi in control and PSC patients from Fig 2B. Immunofluorescence for BSEP (magenta) and F‐actin (green). Scale bar 2 μm.

Indeed, we found that hepatocytes in PSC patients markedly accumulate apical bulkheads (Figs 2A and B, and EV2A and B), similar to BDL and MDR2 KO mice. We confirmed the identity of bile canaliculi by co‐staining with the apical bile salt transporter BSEP (Fig EV2A and B). Remarkably, we found tissue areas with aberrant canaliculi that were formed by multiple hepatocytes arranged around a central lumen that was again devoid of apical bulkheads (Fig 2C, Rosette). These structures are in stark contrast to the normal bile canaliculi formed by two juxtaposed hepatocytes, and rather resemble the bile duct morphology (Fig 2C). Morphologically similar histopathological alterations were described in primary biliary cholangitis (PBC) (Nagore *et al*, 1989), focal nodular hyperplasia (Butron Vila *et al*, 1984) and chronic bile duct ligation in rats (Song *et al*, 1998). They were termed cholestatic liver cell rosettes to differentiate them from hepatitis liver cell rosettes (Nagore *et al*, 1989). The role of liver cell rosettes in ductular metaplasia/atypical ductular reaction has been suggested but was never demonstrated (Nagore *et al*, 1989; Song *et al*, 1998).

In the 3D reconstruction of the bile canaliculi network, it became apparent that the rosettes detected in PSC livers were transversal cross‐sections of segments of epithelial tubes formed by multiple cells, instead of forming narrow canaliculi (Fig 2D and E; Movies EV2 and EV3). Although these multicellular structures resemble the bile duct morphology and, thus, could be mistaken for bile ducts, they are formed by hepatocytes, as confirmed by a number of parameters such as presence of binucleated cells, position away from the portal vein, as well as molecular markers, e.g. co‐staining with BSEP (Fig 2F; although the signal is lower than in hepatocytes forming normal bile canaliculi, see below).

Our observations show that apical bulkheads also accumulate in human patients with primary sclerosing cholangitis. This finding is in line with the aforementioned results suggesting a common response of hepatocytes towards impaired bile flow. It turns out that under these conditions, the absence of apical bulkheads coincides with the formation of liver cell rosette structures that are formed by several (> 2) hepatocytes sharing the lumen circumference.

### The majority of liver cell rosettes in PSC patients do not acquire typical hepatobiliary cell markers

**Figure 3:**
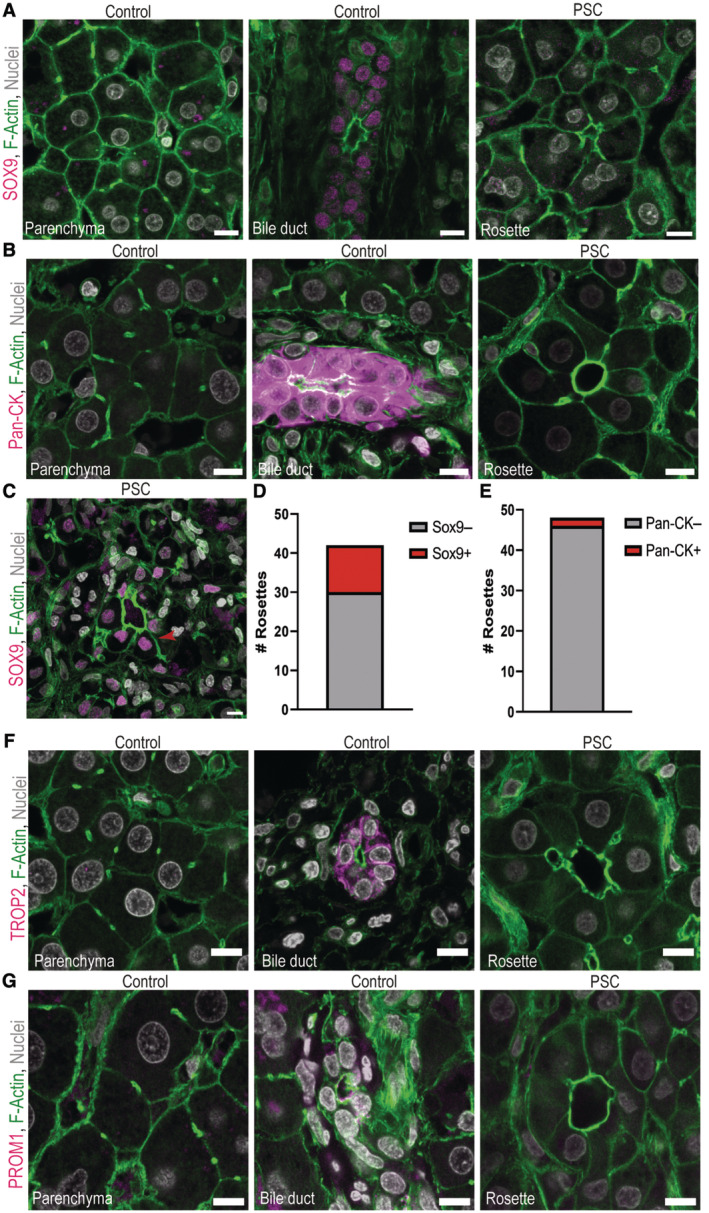
The majority of liver cell rosettes in PSC patients do not acquire typical hepatobiliary cell markers A–DLiver cell rosettes in PSC patients do not acquire other markers of bile duct cells or intermediate hepatobiliary cells. Immunofluoresence for different bile duct cell or intermediate hepatobiliary cell type marker (magenta), F‐actin (green) and nuclei (grey) in healthy parenchyma (left) and bile duct (middle) in control patients and liver cell rosettes (right) in PSC patients (*N* = 3 control patients and *N* = 4 PSC patients). Scale bar 10 μm. (A) SOX9, (B) Pan‐CK, (C) TROP2 and (D) CD133/PROM1. Representative images are shown.EIndividual liver cell rosettes were positive for the bile duct cell marker SOX9. Immunofluoresence for SOX9 (magenta), F‐actin (green) and nuclei (grey). Scale bar 10 μm. Arrowhead marks connection between rosette and bile canaliculi.FQuantification of SOX9‐positive rosettes in PSC patients (*N* = 4 PSC patients).GQuantification of pan‐CK‐positive rosettes in PSC patients (*N* = 3 PSC patients). Source data are available online for this figure.

Liver cell rosettes in PBC and chronic bile duct ligation have been suggested to represent a form of ductular reaction (Nagore *et al*, 1989; Song *et al*, 1998). Based on their shape, bi‐nucleated appearance and positive staining with BSEP at the apical membrane cells forming rosettes in PSC closely resemble hepatocytes. Nevertheless, the BSEP staining in cells forming rosettes is lower than in adjacent typical bile canaliculi (Fig 2F). This could be either because the BSEP signal is distributed over a bigger canalicular surface or because these hepatocytes produce less BSEP in a process of de‐/transdifferentiation. Since the rosettes in PSC resemble bile duct morphology, we asked whether they also acquire bile duct cell markers. The vast majority of liver cell rosettes in PSC patients were negative for SOX9 (Fig 3A) and pan‐cytokeratin (pan‐CK; Fig 3B). Strikingly, we still observed ~ 28% of rosettes with hepatocytes that acquired the bile duct cell transcription factor SOX9 (Fig 3C and D). However, only ~ 4% of the rosettes were positive for pan‐CK (Fig 3E). This is in contrast to the reactive bile ducts accumulating during the ductular reaction (Sato *et al*, 2019) and also the cholestatic liver cell rosettes described in PBC (Nagore *et al*, 1989) that express high levels of cytokeratin.

To further investigate the role of PSC liver cell rosettes in the ductular reaction, we also analysed the acquisition of TROP2 and CD133/PROM1. TROP2 and CD133/PROM1 are expressed in adult bile duct cells (Karbanová *et al*, 2008; Aizarani *et al*, 2019), but both are also associated with intermediate hepatobiliary cells (or oval cells) that can form reactive bile ducts in injured mouse livers (Okabe *et al*, 2009). However, none of the rosettes in any of the patients analysed were positive for TROP2 (Fig 3F) or CD133/PROM1 (Fig 3G). These results suggest that the hepatocytes forming rosettes only rarely acquire bile duct cell markers, and they do not fully convert into an intermediate hepatobiliary cell type. Therefore, although morphologically similar, the rosettes in PBC and PSC might be different. Yet, it is remarkable that a fraction of rosettes become Sox9 positive, although the functional relevance of this finding requires further investigation.

### Liver cell rosettes occur throughout the liver lobule and correlate with elevated canalicular pressure in PSC patients

**Figure 4:**
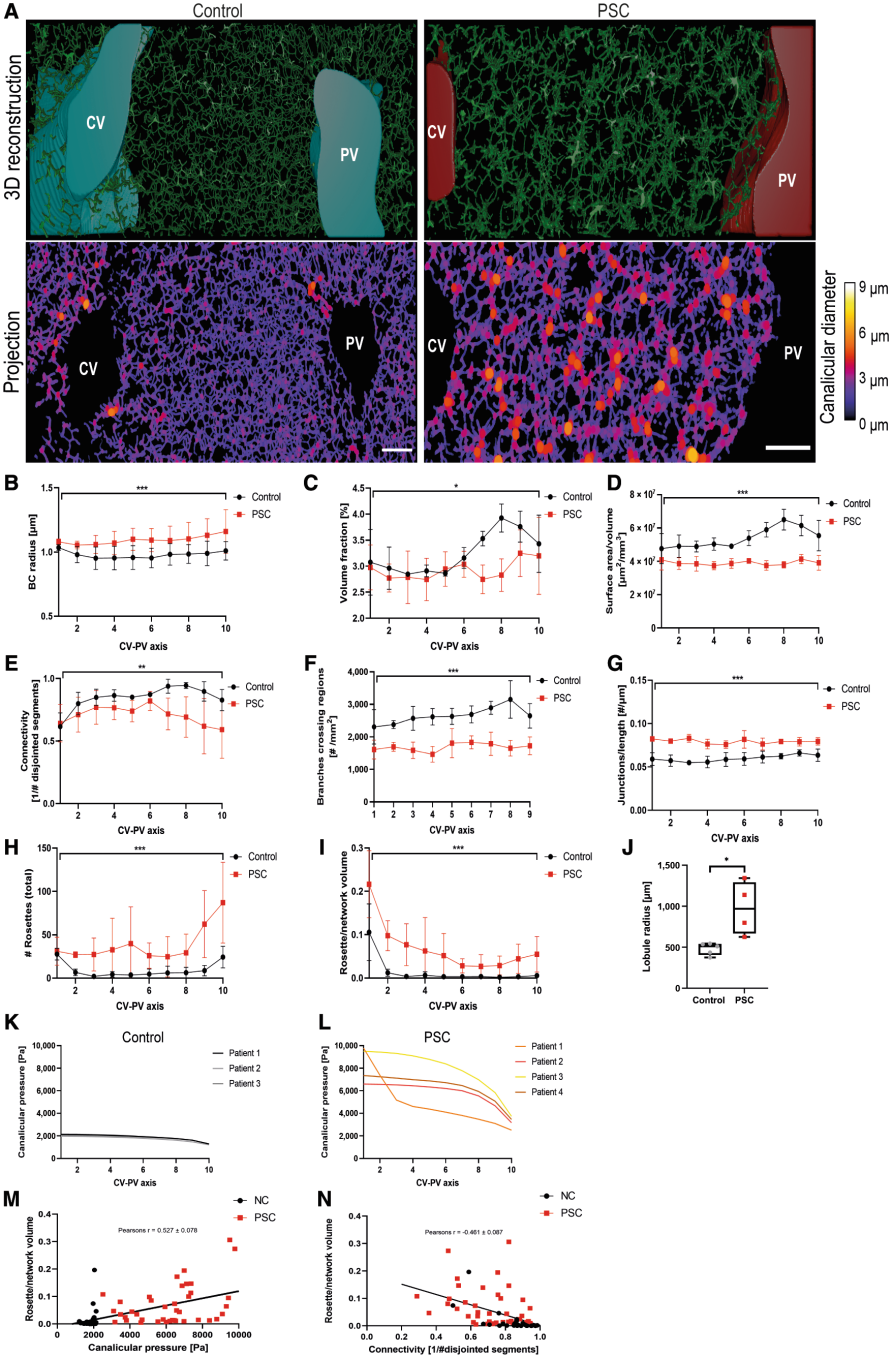
Liver cell rosettes occur throughout the liver lobule and correlate with elevated canalicular pressure in PSC patients A–I3D reconstruction of the bile canaliculi network reveals fundamental changes in the network architecture of PSC patients. (A) Top panel: 3D‐reconstructed bile canaliculi network across full central vein to portal vein (CV‐PV) axes in control (left) and PSC (right) patients. Bottom panel: Projection of the 3D reconstruction. Colours correspond to the mean bile canaliculi diameter in μm. Scale bar 100 μm. (B–I) Quantification of bile canaliculi network characteristics from the 3D reconstruction. Represented are mean values with standard deviation of control and PSC patients at different zones across the CV‐PV axis. 0 represents the zone surrounding the CV and 10 the area surrounding the PV. *N* = 3 with 1× axis/patient for control patients and *N* = 4 with 3× axis/patient for PSC patients. Paired *t*‐test *P < 0.05, **P < 0.01, ***P < 0.001. (B) bile canaliculi radius [μm], (C) volume fraction [%], (D) surface area/volume (μm^2^/mm^3^), (E) connectivity (1/# disjointed bile canaliculi), (F) branches crossing regions [#/mm^2^], (G) junctions/length [#/μm], (H) # rosettes and (I) ratio of rosettes/network volume.JIncreased lobule size in PSC patients. Quantification of the mean lobule radius [μm] in liver tissue of control (*N* = 5) and PSC patients (*N* = 4). Unpaired *t*‐test *P < 0.05. Boxplot shows the 25^th^ and 75^th^ percentiles and the central band shows the median value. Whiskers extend to the min and max values.K, LMathematical model predicts elevated canalicular pressure in PSC patients. Represented is the median canalicular pressure [Pa] across the CV‐PV axis for individual patients. 0 represents the zone surrounding the CV and 10 the area surrounding the PV. *N* = 3 with 1× axis/patient for control and *N* = 4 with 3× axis/patient for PSC patients. (K) Control patients and (L) PSC patients.MThe fraction of canaliculi network occupied by liver cell rosettes positively correlates with canalicular pressure. Pearson's correlation analysis between the rosette/network volume and the predicted canalicular pressure [Pa] in each zone across the CV‐PV axis of all patients analysed (*N* = 3 for control and *N* = 4 for PSC patients) (*r* = 0.527).NThe fraction of canaliculi network occupied by liver cell rosettes negatively correlates with canaliculi network connectivity. Pearson's correlation analysis between the rosette/network volume and canaliculi network connectivity [1/#disjointed segments] in each zone across the CV‐PV axis of all patients analysed (*N* = 3 for control and *N* = 4 for PSC) (*r* = −0.461). Source data are available online for this figure.

The identification of rosette‐like structure formed by hepatocytes was based on high‐resolution imaging, thus limiting the analysis to a small view field. To gain a broader and quantitative view of the changes, we analysed the architecture of the bile canaliculi network across the central vein (CV) to portal vein (PV) axis using deep imaging and 3D reconstruction (Morales‐Navarrete *et al*, 2015; Segovia‐Miranda *et al*, 2019). The magnitude of the bile canaliculi network expansion can be appreciated from the heatmap where colours correspond to bile canaliculi diameter (Fig 4A; Movies EV4 (Control) and EV5 (PSC)). This analysis revealed that rosettes appear throughout the CV‐PV axis and are not directly connected to pre‐existing bile ducts. This is different from the cholestatic liver cell rosettes described in PBC (Nagore *et al*, 1989) or a typical ductular reaction (Gouw *et al*, 2011), which are directly connected to the bile ducts.

Analysis of full CV‐PV axes in PSC patients showed an increase in the mean bile canaliculi radius of around 11.6% (Fig 4B). Dilation of the bile canaliculi is a known feature of cholestasis (Jansen *et al*, 2017). Interestingly, despite an increase in mean bile canaliculi radius, the overall mean network volume decreased by around 9.9%, which is especially pronounced in the PV zone (Fig 4C). Consistent with this, we measured an overall decrease in various parameters, such as 27.7% of the surface area/volume (Fig 4D), 16.5% of connectivity (Fig 4E) and 36.5% of the number of branches crossing zones (Fig 4F) in PSC patients. The number of junctions/length (Fig 4G) was overall increased by about 32.5%.

We additionally aimed to quantify the rosettes across the CV‐PV axis (Fig 4H). We defined rosettes with a stringent cut‐off of > 6 μm bile canaliculi diameter. Because the rosettes vary in size, we estimated their volume and normalized it to the overall bile canaliculi network volume (Fig 4I). This ratio showed that, even though there were more rosettes in PV zones, compared to the network volume, they occupied more of the network in the CV zones. We also found a significant increase in median lobule radius from 512 μm in NC patients to 968 μm in PSC patients (Fig 4J).

Impaired bile flow is a characteristic feature of primary sclerosing cholangitis (Dyson *et al*, 2018). Since apical bulkheads are load‐bearing mechanical elements that raise the resistance of the bile canaliculi against increased canalicular pressure (Bebelman *et al*, 2023), we aimed to investigate whether the described rosettes in PSC are also associated with increased canalicular pressure. Because biliary pressure inside the bile canaliculi cannot be measured directly, we used a computational biliary pressure model (Meyer *et al*, 2017; Segovia‐Miranda *et al*, 2019). In order to predict the effect of the network geometry on biliary pressure changes in the bile canaliculi network of PSC patients, we kept parameters of bile load, bile viscosity and bile flow through the bile ducts similar to control conditions (see Materials and Methods). In control patients, the median biliary pressure inside the bile canaliculi network across the CV‐PV axis is predicted to be 1,922 Pa. The network has a one‐way architecture with a decrease in pressure from pericentral to periportal areas that enforces bile flow opposite to blood flow. Thus, there is a median elevation of 754 Pa pericentral over periportal pressure (Fig 4K). In contrast, for patients with PSC, the model predicted an increase in the median canalicular pressure across the CV‐PV axis to 6,536 Pa, with a median elevation of 5,112 Pa pericentral over periportal pressure (Fig 4L).

Although bile canalicular pressure cannot be measured directly, there are multiple manometric studies in the common bile duct of humans and mice. In the absence of insulation (like valves or sphincters), the pressures inside the bile canaliculi have to be equal or higher than the pressures measured inside the common bile ducts. These studies show direct experimental evidence of a baseline common bile duct pressure in humans between 500 and 1,500 Pa (Csendes *et al*, 1979; Wong *et al*, 1980; Beltrán & Beltrán, 2021). In patients with gallstones, this pressure is elevated in the range of 2,000 Pa but can reach also values of 5,000 Pa. Based on these values, we conclude that the canalicular pressures predicted in our model are physiological and in line with prior measurements.

We suggested correlation between the reorganization of the BC network with the formation of rosettes and increased biliary pressure in patients with PSC. Therefore, we plotted for each position on the CV‐PV axis the rosette/network volume, i.e. the network volume that is occupied by rosettes (identified in Fig 4I), against the predicted canalicular pressure (identified in Fig 4K and L). We performed a Pearson's correlation analysis and found a positive correlation (Fig 4M, *r* = 0.527). In contrast, there is a negative correlation between the rosette/network volume and the bile canaliculi network connectivity (identified in Fig 4E) (Fig 4N, *r* = −0.461).

In summary, these results highlight hitherto unrecognized structural changes in the bile canaliculi network and, more importantly, suggest that with increasing canalicular pressure, the liver cell rosettes occupy more volume of the canalicular network and reduce the overall network connectivity.

### Liver cell rosettes occur already in early‐stage PSC patients

**Figure 5:**
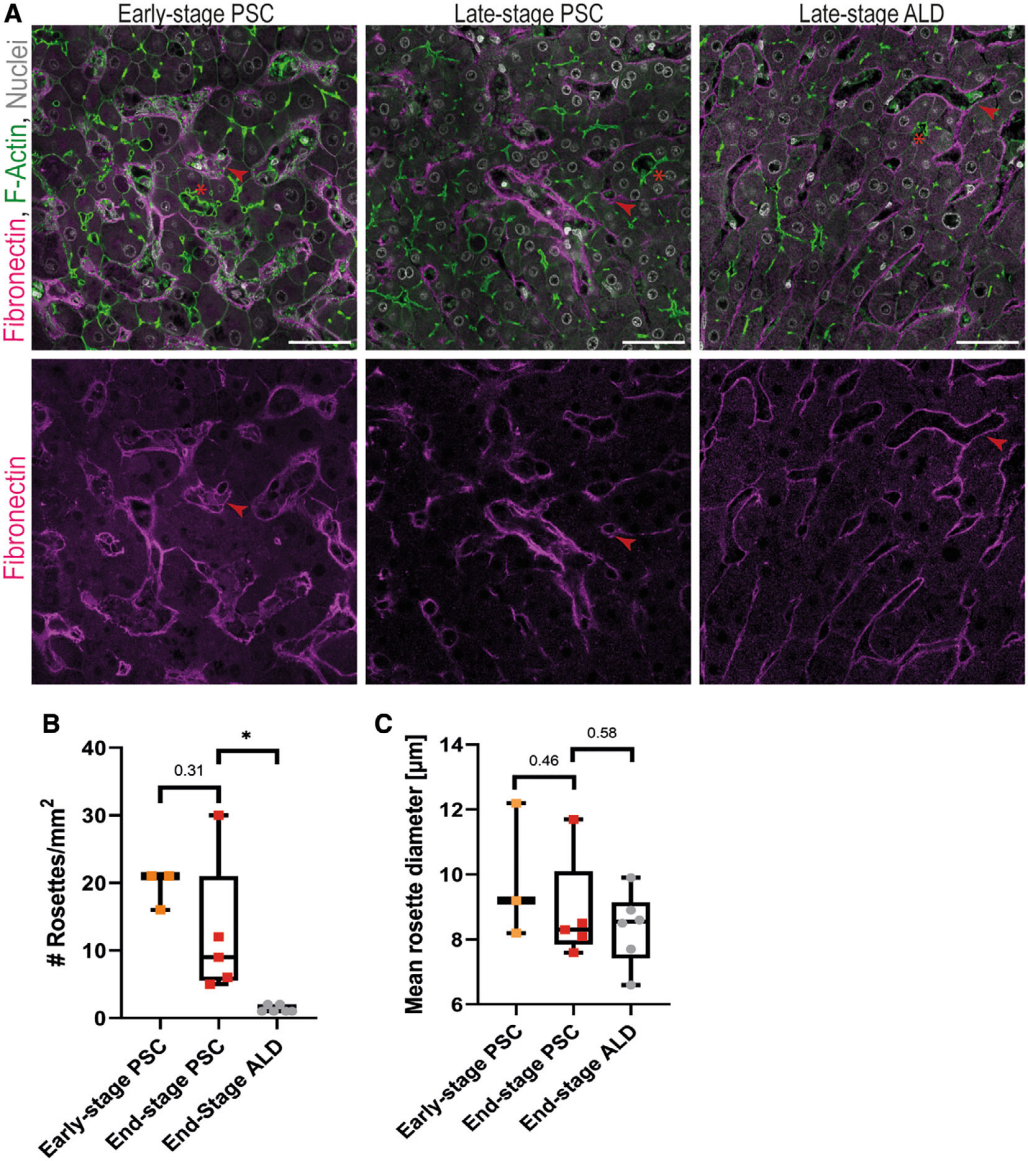
Liver cell rosettes occur already in early‐stage PSC patients Liver cell rosettes occur in early‐ and late‐stage PSC patients and in end‐stage alcoholic liver disease (ALD) patients. Immunofluorescence of fibronectin (magenta), F‐actin (green) and nuclei (grey) in early‐stage PSC (*N* = 3), late‐stage PSC (*N* = 5) and end‐stage ALD (*N* = 5) patients showing liver cell rosettes. Representative images are shown. Scale bar 50 μm. Examples of rosettes are labelled with a red asterisk and examples of sinusoids are labelled with a red arrowhead.Similar numbers of liver cell rosettes in early‐stage and end‐stage PSC patients. Quantification of the total number of rosettes in early‐stage PSC (*N* = 3), late‐stage PSC (*N* = 5) and end‐stage ALD (*N* = 5) patients. Unpaired *t*‐test *P < 0.05.Similar mean diameter of analysed liver cell rosettes in different liver diseases. Quantification of the mean rosette diameter in early‐stage PSC (*N* = 3), late‐stage PSC (*N* = 5) and end‐stage ALD (*N* = 5). Unpaired *t*‐test. Data information: Boxplot shows the 25^th^ and 75^th^ percentiles and the central band shows the median value. Whiskers extend to the min and max values. Source data are available online for this figure.

Based on the correlation between rosette formation and elevated canalicular pressure in PSC, we aimed to investigate the formation of rosettes during disease progression and their use as putative histopathological marker. We analysed liver cell rosettes in tissues from early‐ and end‐stage PSC patients from a different patient cohort than for the analysis in Fig 4. The analysis was performed in 2D on single tissue sections with a total area of ~ 3.2 mm^2^ per patient, similar to what is performed in standard histological practice. Rosettes were again defined as canaliculi with diameter > 6 μm formed by > 2 hepatocytes sharing the same lumen. Interestingly, there was neither significant difference in the total number of rosettes nor the mean rosette diameter between early‐ and late‐stage PSC patients (Fig 5A–C). The severe degree of fibrosis in late‐stage PSC patients reduces the overall area of parenchyma and explains the slight decrease in number of rosettes/$mm^{2}$. To determine whether liver cell rosettes are a common histopathological alteration in different end‐stage liver diseases, we further analysed tissue from patients with alcoholic liver disease (ALD). ALD patients can present different signs of cholestasis. However, there is no clear link between impaired bile flow and ALD pathology (Trinchet *et al*, 1994; Tung & Carithers, 1999). Our analysis shows the abundance of liver cell rosettes in all analysed ALD patients, although significantly less than in end‐stage PSC patients (Fig 5B). Interestingly, the size of the quantified rosettes was similar between the diseases (Fig 5C).

In summary, these results suggest that liver cell rosettes are a distinct tissue feature that is pronounced already in early‐stage PSC and can be detected with common histological techniques. We propose them as histopathological features for PSC.

### Primary hepatocytes and organoids *in vitro* form rosette‐like bile canaliculi upon bile acid treatment

Our results are consistent with apical bulkheads accumulating as a response to elevated lumina pressure and the formation of aberrantly expanded lumina in the liver cell rosettes as a consequence of their loss. To test this hypothesis and determine whether elevated canalicular pressure is indeed causal for rosette formation, we directly examined the response of hepatocytes to canalicular pressure using primary mouse hepatocytes in collagen sandwich culture and 3D hepatocyte organoids.

**Figure 6:**
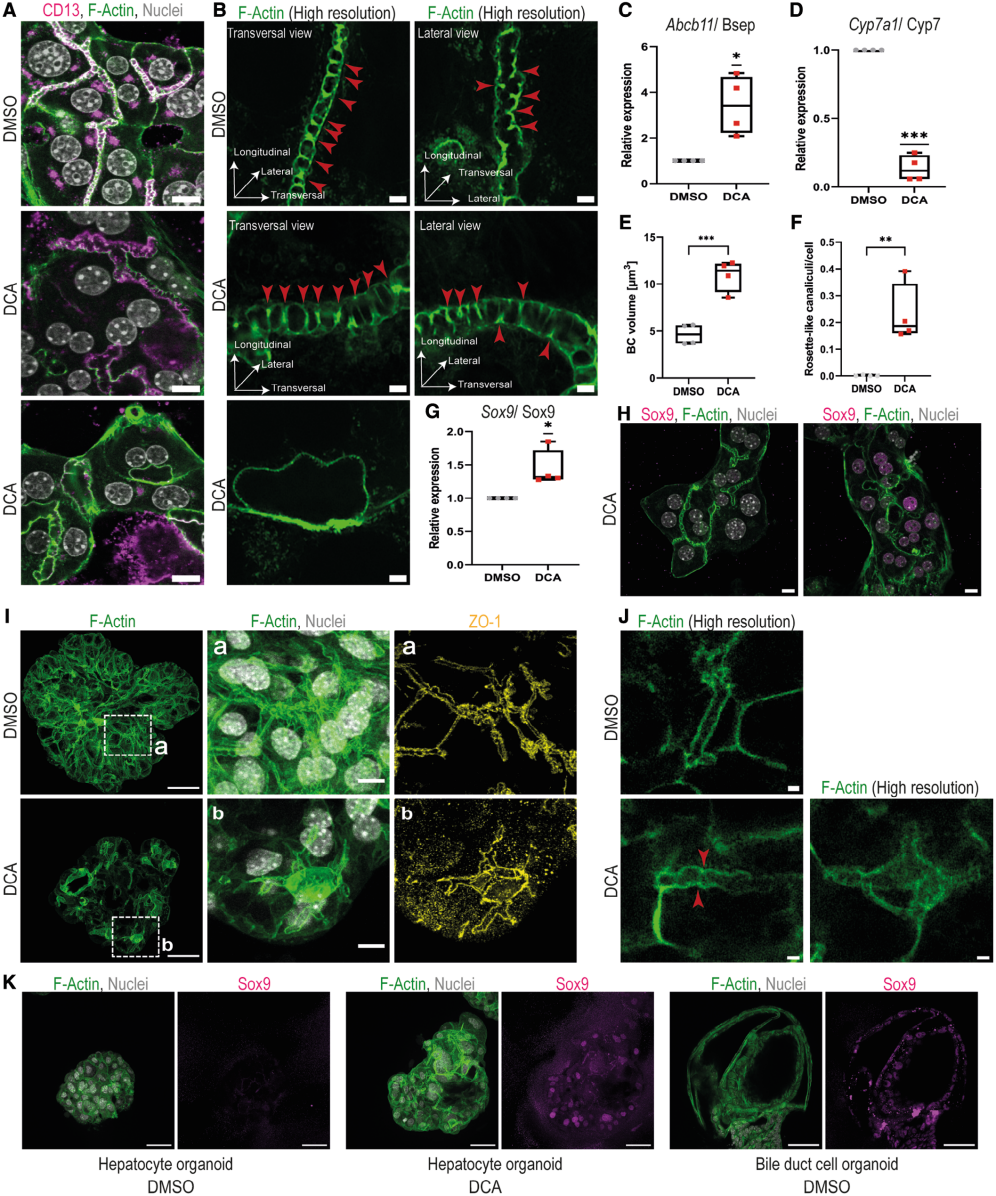
Primary hepatocytes and organoids *in vitro* form rosette‐like bile canaliculi upon bile acid treatment ADifferent responses of the bile canaliculi towards increased pressure. Primary hepatocytes treated with DMSO or 200 μM DCA for 16 h and stained for F‐actin (green) and nuclei (white). Top panel: DMSO‐treated control, middle panel: 200 μM DCA showing canalicular dilation and bottom panel: 200 μM DCA showing aberrant, rosette‐like bile canaliculi without apical bulkheads. Representative images are shown. Scale bar 10 μm.BHigh‐resolution microscopy images of individual bile canaliculi stained for F‐actin (green) in primary hepatocytes treated with DMSO or 200 μM DCA. Red arrows indicate apical bulkheads. Representative images are shown. Scale bar 2 μm.C, DDCA treatment induces a cholestatic response in primary hepatocytes. Gene expression analysis of genes involved in bile acid metabolism and transport in primary hepatocytes treated with DMSO or 200 μM DCA for 16 h. Represented are mean Cq values normalized to GAPDH expression (*N* = 4 biological replicates). One sample *t*‐test *P < 0.05, ***P < 0.001. (C) *Abcb11*/Bsep and (D) *Cyp7a1*/Cyp7.EDCA treatment induces dilation of bile canaliculi in primary hepatocytes. Quantification of the mean bile canaliculi volume in μm^3^ of primary hepatocyte treated with DMSO or 200 μM DCA for 16 h (*N* = 4 biological replicates). Unpaired *t*‐test ***P < 0.001.FInduction of rosette‐like bile canaliculi in primary hepatocytes after DCA treatment. Quantification of the mean number of rosette‐like bile canaliculi per hepatocyte after treatment with DMSO or 200 μM DCA for 16 h (*N* = 4 biological replicates). Unpaired *t*‐test **P < 0.01.GIncreased expression of the bile duct cell transcription factor Sox9 in primary hepatocytes after DCA treatment. Gene expression analysis of Sox9/Sox9 in primary hepatocytes treated with DMSO or 200 μM DCA. Represented are mean Cq values normalized to GAPDH expression and whiskers show min and max values (*N* = 4 biological replicates). One‐sample *t*‐test *P < 0.05.HDCA‐treated primary hepatocytes with rosette‐like canaliculi become positive for the bile duct cell transcription factor Sox9. Immunofluorescence for Sox9 (magenta), F‐actin (green) and nuclei (white) in primary hepatocytes treated with 200 μM. Representative images are shown. Scale bar 10 μm.I, JDCA treatment in 3D hepatocyte organoids also induces canalicular dilation and rosette‐like canaliculi and Sox9 expression. Hepatocyte organoids were treated with DMSO or 200 μM DCA for 24 h (*N* = 3 biological replicates). (I) Maximum projection of full organoids stained for F‐actin (green). Scale bar 50 μm. Inset A and B. Maximum projection of zoomed‐in parts of the bile canaliculi network in DMSO‐treated (A) and DCA‐treated (B) hepatocyte organoids stained with F‐actin (green), nuclei (white) and the tight junction protein ZO‐1 (yellow). Scale bar 10 μm. (J) High‐resolution microscopy of individual bile canaliculi in DMSO‐treated and DCA‐treated hepatocyte organoids stained for F‐actin (green). Red arrows indicate apical bulkheads. Scale bar 2 μm.KMaximum projection of F‐actin (green) and nuclei (white), and the bile duct cell marker Sox9 (magenta) in DMSO‐treated and DCA‐treated hepatocyte organoids (*N* = 3 biological replicates). Untreated bile duct cell organoids serve as positive control. Scale bar 50 μm. Data information: Boxplots show the 25^th^ and 75^th^ percentiles and the central band shows the median value. Whiskers extend to the min and max values.

**Figure EV3:**
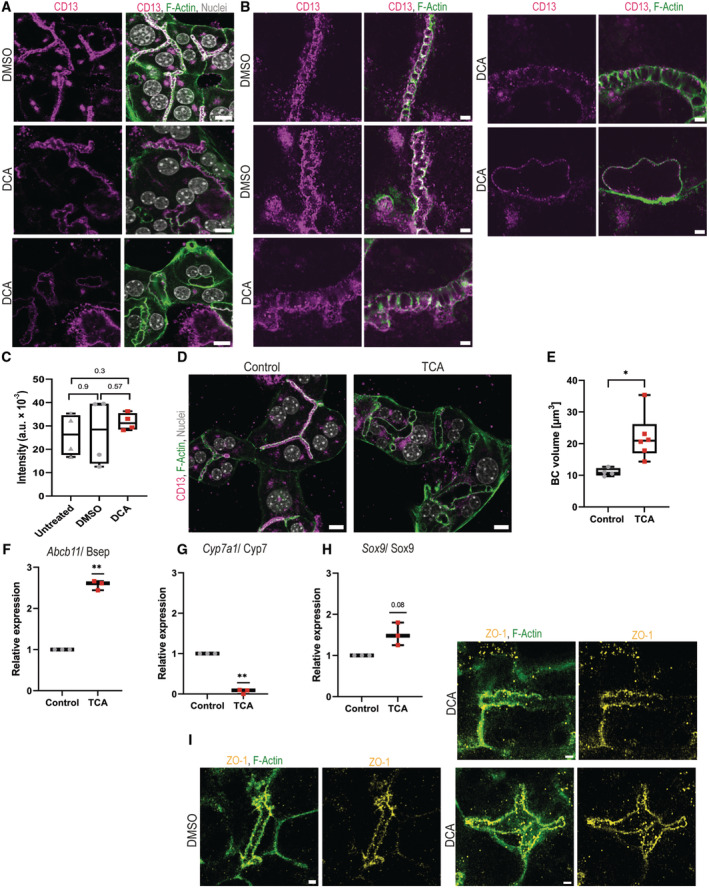
*In vitro* systems upon DCA and TCA treatments APrimary hepatocytes treated with DMSO or 200 μM DCA for 16 h from Fig 6A. Individual channels of immunofluorescence for CD13 (magenta), F‐actin (green) and nuclei (grey). Scale bar 10 μm.BHigh‐resolution microscopy images of individual bile canaliculi in primary hepatocytes treated with DMSO or 200 μM DCA from Fig 6B. Immunofluorescence for CD13 (magenta) and F‐actin (green). Scale bar 2 μm.CDCA treatment does not affect primary hepatocyte viability. Untreated, DMSO‐ and DCA‐treated primary hepatocytes after 16 h treatment showed no changes in fluorescence intensity of alamarBlue cell viability reagent indicating no changes in cell viability (*N* = 4 biological replicates). Unpaired *t*‐test.DPrimary hepatocytes treated with water or 50 μM TCA for 16 h. Individual channels of immunofluorescence for CD13 (magenta), F‐actin (green) and nuclei (grey). Scale bar 10 μm.EQuantification of the mean bile canaliculi volume in μm^3^ of primary hepatocyte treated with water and 50 μM TCA (*N* = 2 biological replicates, technical replicates shown). Unpaired *t*‐test *P < 0.05.F–HGene expression analysis of (F) *Abcb11*/BSEP, (G) *Cyp7a1*/Cyp7 and (H) *Sox9*/Sox9 in primary hepatocytes treated with water or 50 μM DCA. Represented are mean Cq values normalized to GAPDH expression and whiskers show min and max values (*N* = 3 biological replicates). One‐sample *t*‐test **P < 0.01.IHepatocyte organoids from Fig 6J with immunofluorescence for ZO‐1 (yellow) and F‐actin (green). Scalebar 2 μm. Data information: Boxplots show the 25^th^ and 75^th^ percentiles and the central band shows the median value. Whiskers extend to the min and max values.

Bile canaliculi are formed by the apical plasma membranes of typically only two juxtaposed hepatocytes surrounded by a dense actin cortex that can be visualized by immunofluorescence microscopy (Figs 6A and EV3A). In primary hepatocytes cultured in collagen sandwich culture, the absence of biliary drainage leads bile acids to accumulate over time, increasing the canalicular bile pressure already in the untreated state. Under these conditions, apical bulkheads are prominent and can be visualized as periodic F‐actin stripes inside the bile canaliculi lumen using high‐resolution confocal microscopy (Figs 6B and EV3B) (Zeigerer *et al*, 2017; Belicova *et al*, 2021). Depending on the bile canaliculi orientation with respect to the focal plane, the F‐actin cortex of the apical bulkheads appears to cross the lumen completely (transversal view) or only partially (lateral view).

Next, we aimed to mimic *in vitro* the effects on bile canalicular pressure observed *in vivo*. To increase the canalicular pressure beyond the levels of the cellular system, we treated the hepatocytes with the bile acid deoxycholic acid (DCA). The choice of this particular bile acid is justified by the fact that hydrophobic bile acids such as DCA are known to have more choleretic effects than primary conjugated bile acids (Tavoloni *et al*, 1985). We applied DCA at a concentration of 200 μM, within the range of total bile acids accumulating in the serum of mice after bile duct ligation (Zhang *et al*, 2012). Since bile acids can be cytotoxic and induce apoptosis at higher doses, we took care to exclude severe cell death upon 16 h DCA treatment using a cell viability assay (Fig EV3C). The induction of cholestasis was verified by the gene expression changes in two well‐established cholestasis markers: *Abcb11*/Bsep (Fig 6C), the main apical bile acid exporter, and *Cyp7a1*/Cyp7, a rate‐limiting enzyme in bile acid synthesis (Fig 6D).

DCA treatment led to bile canaliculi dilation and volume increase (Fig 6E). Apical bulkheads were maintained by most hepatocytes (Fig 6A and B, middle panel). However, a fraction of hepatocytes was devoid of apical bulkheads and, interestingly, formed rosette‐like bile canaliculi with a spherical shape of up to 10 μm diameter and were often lined by multiple hepatocytes (Fig 6A and B, bottom panel, Fig 6F). We asked whether the hepatocytes forming rosette‐like bile canaliculi also acquired Sox9 gene expression, similar to a small fraction of rosettes in PSC patients (Fig 3C and D). In DCA‐treated hepatocytes, we detected an overall upregulation of the bile duct cell transcription factor Sox9 (Fig 6G). Interestingly, Sox9 expression was confined to hepatocytes devoid of apical bulkheads and lining the aberrant large bile canaliculi, whereas hepatocytes forming typical bile canaliculi with apical bulkheads were negative (Fig 6H).

To exclude that bile canalicular dilation is unique to one bile acid, we additionally treated primary hepatocytes with the sodium‐conjugated bile acid taurocholic acid (TCA). TCA is particularly relevant because it is detected at high concentration in the serum of PSC patients (Trottier *et al*, 2012). Similar to DCA, treatment with TCA also induces dilation of the bile canaliculi (Fig EV3D and E) and gene expression changes in cholestatic markers (Fig EV3F and G). The gene expression of Sox9 also tended towards an increase but was not significantly changed (Fig EV3H).

To rule out the possibility that the morphological and transcriptional alterations of hepatocytes observed upon DCA treatment are unique to the 2D *in vitro* culture conditions, we performed a similar analysis on hepatocyte organoids where hepatocytes grow bile canaliculi in 3D. In control organoids, hepatocytes formed narrow and well‐connected bile canaliculi (Fig 6I). Interestingly, in such arrangement of bile canaliculi, the apical bulkheads were less prominent than in the 2D *in vitro* culture (Fig 6J). In contrast, upon DCA treatment, the bile canaliculi in the hepatocyte organoids became dilated and the majority accumulated prominent apical bulkheads (Figs 6I and J, bottom panel and EV3I). Similar to the 2D sandwich culture, DCA treatment induced several rosette‐like bile canaliculi lacking apical bulkheads, similar to the rosettes seen in PSC patients. These lumina also reached a diameter of up to 10 μm, were surrounded by multiple cells and remained well connected with the rest of the network (Fig 6I and J). Furthermore, DCA‐treated hepatocyte organoids also acquired the bile duct cell marker Sox9 with similar levels as control‐treated bile duct cell organoids (Fig 6K).

These results show that bile acid treatment causes dilation of the bile canaliculi formed by hepatocytes that accumulate apical bulkheads. However, hepatocytes that lack apical bulkheads in this dilated stage form rosette‐like structures instead of usual bile canaliculi.

### Elevated pressure directly drives canalicular dilation and loss of apical bulkhead with rosette‐like bile canaliculi morphology

**Figure 7:**
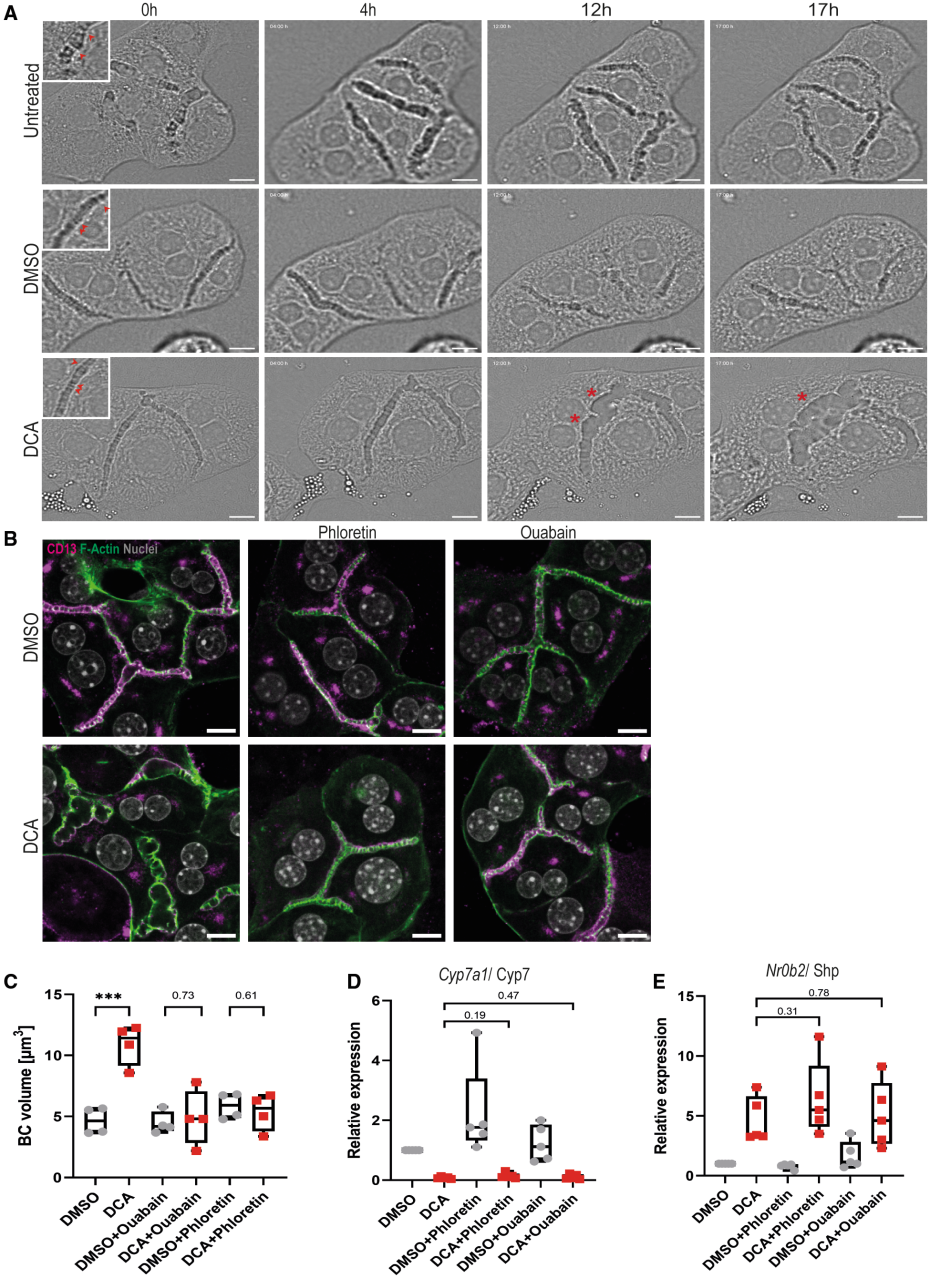
Elevated pressure directly drives canalicular dilation and loss of apical bulkhead with rosette‐like bile canaliculi morphology ARosette‐like canaliculi with reduced number of apical bulkheads can emerge from usual canaliculi with accumulated apical bulkheads. Time‐lapse imaging with brightfield phase contrast of primary hepatocytes either untreated (top row), DMSO treated (middle row) or DCA treated (bottom row). An image was acquired every 15 min. Representative images are shown (*N* = 3 biological replicates). Inserts show magnification of bile canaliculi with red arrows pointing towards apical bulkheads. Red stars mark areas in between apical bulkheads that markedly bulge out. Scale bar 10 μm.BPharmacological inhibition of canalicular water influx can prevent canalicular dilation upon DCA treatment. Confocal microscopy of CD13 (magenta), F‐actin (green) and nuclei (white) in primary hepatocytes treated with DMSO and 200 μM DCA with 200 μM phloretin and 1 μM ouabain for 16 h. Scale bar 10 μm. Representative images are shown.CQuantification of the mean bile canaliculi volume in μm^3^ of primary hepatocyte treated with DMSO and 200 μM DCA with 200 μM phloretin and 1 μM ouabain for 16 h (*N* = 4 biological replicates). Unpaired *t*‐test ***P < 0.001.D, EPharmacological inhibitors do not affect key bile acid signalling pathways. Gene expression analysis of different genes involved in bile acid signalling in primary hepatocytes treated with DMSO or 200 μM DCA with 200 μM phloretin and 1 μM ouabain for 16 h. Represented are mean Cq values normalized to GAPDH expression and whiskers show min and max values (*N* = 5 biological replicates). Unpaired *t*‐test. (D) *Cyp7a1*/Cyp7 and (E) *Nr0b2*/Shp. Data information: Boxplots show the 25^th^ and 75^th^ percentiles and the central band shows the median value. Whiskers extend to the min and max values.

Our results suggest that in a state of increased intraluminal pressure, the absence of apical bulkheads is directly linked to the formation of rosette‐like bile canaliculi. It is unclear whether these emerge directly from hepatocytes unable to assemble apical bulkheads or if these were first assembled and subsequently disrupted. Time‐lapse imaging of untreated and DMSO‐treated primary hepatocytes showed that bile canaliculi and apical bulkheads are dynamic and remodel over time (Fig 7A; Movies EV6 (Untreated control), EV7 (DMSO‐treated) and EV8 (DCA‐treated)), consistent with previous reports (Phillips *et al*, 1982). Similarly, apical bulkheads appeared to be dynamic and constantly remodelled (Fig 7A, top and middle panel). Already after 1 h of DCA treatment, bile canaliculi were dilated and apical bulkheads were well visible as stripes inside the lumen. Bile canaliculi remodelling was pronounced upon DCA treatment where most bile canaliculi performed repeated cycles of swelling, vesicle budding and collapsing. As swelling progressed, bile canaliculi became spherical, and the apical bulkheads first protruded less deep into the lumen until they eventually disappeared and formed large, rosette‐like canaliculi (Fig 7A, bottom panel). We conclude that the rosette‐like bile canaliculi emerge from hepatocytes that subsequently lose apical bulkheads.

Because lumens are balanced by intraluminal pressure and the actomyosin cortex strength canalicular dilation can only occur when the intraluminal pressure exceeds the actomysoin cortex strength. Thus, dilation is caused either by an increase in intraluminal pressure or weakening of the actomyosin cortex. In other words, either there is a direct increase in pressure or the actomyosin cortex is indirectly weakened via bile acid signalling. To distinguish between these two possibilities, we pharmacologically inhibited the inflow of water into bile canaliculi using phloretin and ouabain to prevent an increase in canalicular pressure in the presence of DCA. Phloretin is an inhibitor of aquaporin water channels that has previously been shown to affect bile canaliculi volume upon cholestasis (Huebert *et al*, 2002). Ouabain is a well‐studied inhibitor of Na+/K+‐ATPase, preventing bile salt‐independent bile flow (Hardison & Wood, 1978). In primary hepatocytes, treatment with 200 μM phloretin or 1 μM ouabain to counteract the DCA‐induced increase in canalicular bile pressure rescued the dilation of bile canaliculi and the periodic stripe pattern of apical bulkheads (Fig 7B and C), averting the formation of aberrant, spherical bile canaliculi. The inhibitors did not affect bile acid‐induced downregulation of *Cyp7a1* (Fig 7D) or *Shp* mRNA (Fig 7E), one of the key targets of activated FXR receptor. Hence, inhibiting apical water influx with phloretin and ouabain in the presence of DCA prevents dilation because it prevents an increase in intraluminal pressure although the osmotic gradient and the bile acid signalling effects are still present.

We conclude that apical bulkheads disassemble upon severe swelling of bile canaliculi into a rosette‐like shape driven primarily by elevated canalicular pressure.

## Discussion

Accumulation of bile inside the biliary tree is a common feature of impaired bile flow. The resulting increase in biliary pressure can have important functional consequences for liver physiology and disease. During liver development, hepatocytes assemble transversal connections, termed apical bulkheads, to enforce the elongation of the bile canaliculi (Belicova *et al*, 2021). Here, we could demonstrate that the accumulation of apical bulkheads is also a common response of adult hepatocytes to increased biliary pressure in various liver conditions. This response is consistent with the necessity to stabilize the bile canaliculi structure and preserve the blood–bile barrier. Through combination of *in vitro* studies on primary hepatocytes and organoids and mathematical modelling, it turns out that the loss of apical bulkheads in conditions of impaired bile flow and high intraluminal pressure leads to the formation of aberrant canaliculi in the shape of liver cell rosettes.

The morphological changes detected in the liver tissue in our study are the manifestation of the changes in luminal pressure. Bile canaliculi are formed by the juxtaposition of the apical surfaces of two adjacent hepatocytes (Motta & Fumagalli, 1975). In epithelial cells, the actomyosin cortex underlying the apical surface is under tension and drives morphogenetic events (van Loon *et al*, 2020). It can also respond to mechanical stimuli, such as variations in apical lumen pressure. In the case of the hepatocytes, this implies that bile canaliculi are dynamic structures that can respond to changes in bile pressure under homeostatic and pathological conditions. Several observations support this statement. For example, actomyosin contractility contributes to bile flow in addition to the osmotic pressure of bile acids (Watanabe *et al*, 1991; Meyer *et al*, 2017). In liver cholestasis, the actomyosin system responds to the increased biliary pressure by contracting to induce the formation of bile‐regurgitative vesicles, as a rapid homeostatic mechanism (Gupta *et al*, 2017). The exact pressure inside bile canaliculi in the liver cannot be assessed experimentally and thus studies on the mechanism of bile canalicular pressure are sparse. Lumina, like bile canaliculi, are balanced by the intraluminal pressure and the actomyosin cortex strength. The pressure is built by water influx into the lumen due to an osmotic gradient, which is maintained by osmolyte transporters. Increasing osmolyte concentrations in the bile canaliculi drive an increase in the intraluminal pressure. When this exceeds the actomyosin cortex strength, dilation occurs. Thus, dilation can be caused either by weakening of the actomyosin cortex or by increased intraluminal pressure. Other compensating mechanisms like bile leakage, bile regurgitation or altered bile transporters do play a role, but if dilation occurs, the intraluminal pressure nevertheless has exceeded the actomyosin cortex strength. We inhibited apical water influx with ouabain and phloretin in the presence of DCA to block the increase in intraluminal pressure in the presence of a high osmotic gradient. The fact that this prevents dilation shows that dilation occurs due to an increase in intraluminal pressure and not because of a weaker actomyosin cortex, e.g. through bile acid signalling. Also, it is well‐established that in cholestasis, the canalicular actomyosin cortex is strengthened (Gupta *et al*, 2017) and also DCA treatments raise phosphorylation of myosin light‐chain levels *in vitro* (Meyer *et al*, 2020). Thus, dilation of bile canaliculi under cholestatic conditions is a useful approximation for increased intraluminal pressure. The accumulation of apical bulkheads coincides with the increases in hydrodynamic pressure and canalicular dilation. Apical bulkheads are load‐bearing structures that increase the ability of bile canaliculi to withstand increasing intraluminal pressure (Bebelman *et al*, 2023). Similar to the role of apical bulkheads in the embryonic liver (Belicova *et al*, 2021; Bebelman *et al*, 2023), we interpret the induction of the apical bulkheads as a response to protect the structure of the bile canaliculi upon non‐physiological increase in luminal pressure. This strongly suggests that hepatocytes have a mechanism to directly or indirectly sense canalicular pressure elevation and mount a specific response of the apical cortex.

A remarkable observation was that some hepatocytes forming rosettes acquired the bile duct cell/progenitor marker SOX9. However, these cells did not convert into bile duct cells or an intermediate hepatobiliary cell type. The molecular mechanisms underlying their biogenesis and their functional relevance will have to be investigated in the future. It has been suggested that the cholestatic liver cell rosettes in PBC and chronic bile duct ligation are part of the ductular metaplasia (Nagore *et al*, 1989; Song *et al*, 1998), which is a form of the ductular reaction seen in many liver diseases (Sato *et al*, 2019). These reactive bile ducts can be formed by an intermediate hepatobiliary cell type that is thought to aid liver regeneration (Song *et al*, 1998; Roskams *et al*, 2004; Sato *et al*, 2019). Despite great interest, the emergence of these intermediate hepatobiliary cells is incompletely understood (Gouw *et al*, 2011; Sato *et al*, 2019). Morphologically, they are characterized by a rosette‐like arrangement similar to bile duct cells that are also directly connected to pre‐existing bile ducts and are positive for cytokeratin 7 (Lenzi *et al*, 1992). The liver cell rosettes described here in PSC are different from that, i.e. they are not reactive bile ducts. (i) They form throughout the bile canaliculi network and are not directly connected to the bile ducts. (ii) They are negative for pan‐cytokeratin or the hepatobiliary intermediate cell markers TROP2 or CD133/PROM1. It is unlikely that these rosettes mature into bile ducts as in the ductular reaction because ductular reactions that invade the parenchyma are only described in patients with hepatocellular damage and not biliary disorders (Clerbaux *et al*, 2019). This implies that, although morphologically similar, the cell rosettes in PSC are different from those previously described in other liver diseases.

PSC is a heterogeneous disease difficult to diagnose and currently without therapeutic options to cure or prevent disease onset. Diagnosis is mainly based on elevated serum markers and magnetic resonance cholangiopancreatography (MRCP) detecting characteristic strictures of the bile ducts (Chazouilleres *et al*, 2022). However, both methods are ineffective in detecting early disease stages or intrahepatic PSC where strictures are not visible with MRCP. In such cases, liver biopsies are indicated even though histology has traditionally served a limited role in the diagnosis of PSC patients due to patchy disease affection and lack of specific features (Ponsioen *et al*, 2021). Our results suggest that liver cell rosettes are an early histological feature in PSC. Further investigations of the utility of these findings in clinical practice are needed but the morphological alterations described here could well contribute to novel means of securing disease diagnosis in early stages, prior to the establishment of irreversible fibrosis.

It is striking that liver cell rosettes occur in various disease aetiologies like PBC (Nagore *et al*, 1989), ALD and PSC. Similar bile canalicular dilations and tubular arrangements of hepatocytes occur in focal nodular hyperplasia (FNH; Butron Vila *et al*, 1984) and is also a histopathological feature in hepatocellular carcinoma (HCC) of the pseudoglandular/pseudoacinar type (Brunt, 2012; Schlageter *et al*, 2014). Increased canalicular pressure could be a common cause of rosette formation in various diseases, a possibility that requires further investigation. Since most liver diseases are accompanied by fibrosis, which alters the mechanical properties of the tissue, it would be interesting to assess how this affects hepatocyte polarity and rosette formation.

With our analysis, we provide first insights into the pathogenesis underlying the appearance of liver cell rosettes in PSC and potentially also other liver diseases. First, we noticed that the lumina enclosed in the liver rosettes invariably lack apical bulkheads. The *in vitro* studies suggest that this may be due to the inability of hepatocytes to form or keep the apical bulkheads when the luminal pressure exceeds a threshold value. This interpretation is consistent with the finding that genetic perturbations that cause the loss of apical bulkheads both in cultured hepatoblasts *in vitro* and in developing liver *in vivo* lead to the formation of epithelial‐like tubular structures that resemble the morphology of bile ducts (Belicova *et al*, 2021). Second, our analysis suggests that these pressure‐driven alterations of the hepatocyte apical membrane might affect gene transcription.

Our observations reporting the induction of apical bulkheads upon impaired bile flow and that their absence under these conditions leads to the formation of liver cell rosettes point at the necessity to elucidate the underlying molecular mechanisms and their contributions to disease. The fact that hepatocytes can dynamically respond to alterations in bile pressure raises several questions, which could not be addressed yet in this study. What is the molecular nature of the mechanosensing signalling pathway of hepatocytes that responds to biliary pressure? To what extent does elevated canalicular pressure contribute to disease progression compared to bile acid signalling or toxicity? Given the similarity of bile canaliculi alterations in ALD and PSC, we suspect that the changes in hepatocyte polarity and rosette formation are manifestations of a common mechanism relevant to different liver diseases with elevated biliary pressure. Elucidating the underlying molecular mechanisms is a prerequisite to developing novel therapeutic options.

## Materials and Methods

### Reagents and Tools table

**Table:** 

Primers	Primer sequence (5′–>3′)	
*Abcb11*/Bsep forward	TCTGACTCAGTGATTCTTCGCA	
*Abcb11*/Bsep reverse	CCCATAAACATCAGCCAGTTGT	
*Cyp7a1*/Cyp7 forward	CACCATTCCTGCAACCTTCTGG	
*Cyp7a1*/Cyp7 reverse	ATGGCATTCCCTCCAGAGCTGA	
*Nr02b*/Shp forward	CCTCTTCAACCCAGATGTGCC	
*Nr02b*/Shp reverse	ACCAGGGCTCCAAGACTTCA	
*Sox9*/Sox9 forward	AGTACCCGCATCTGCACAAC	
*Sox9*/Sox9 reverse	TACTTGTAATCGGGGTGGTCT	

	**Source**	**Catalog number**
**Primary antibodies**		
Rabbit anti BSEP	Sigma	HPA019035
Mouse anti CD13	Santa Cruz	sc‐136484
Rat anti CD13	Novus	NB100‐64843
Goat anti DPPIV	R&D	AF1180
Rabbit anti Fibronectin	Lifespan Biosciences	LS‐B2318
Rabbit anti GS	Sigma	G2781
Rabbit anti Pan‐CK	Dako	Z0622
Mouse anti PROM1/CD133	MyBioSource	MBS415235
Rabbit anti SOX9	Abcam	ab185966
Rabbit anti TROP2	Abcam	ab214488
Rabbit anti ZO‐1	Invitrogen	40‐2200
**Secondary antibodies**		
DAPI 1 mg/ml	Sigma	D8417
Donley anti mouse Alexa Fluor 647	Invitrogen	A31571
Donkey anti rabbit Alexa Fluor 647	Invitrogen	A31573
Donkey anti rat CF 568	Biotium	20092
Phalloidin Alexa Fluor 488	Invitrogen	A12379

### Methods and Protocols

#### Animals

Animal experiments were performed on 8‐ to 12‐week‐old, male C57BL/6JOlaHsd (Envigo) mice. MDR2 KO and FVB/N background mice were purchased (The Jackson laboratory, Stock‐Nr. JAX 002539) and male animals at 12 weeks old were used. All experiments were performed in accordance with German animal welfare legislation in pathogen‐free conditions in the animal facility of the Max Planck Institute of Molecular Cell Biology and Genetics (MPI‐CBG), Dresden, Germany. Mice were maintained in a conventional barrier animal facility with a climate‐controlled environment on a 12‐h light/12‐h dark cycle and fed ad libitum with regular rodent chow. Protocols were approved by the Institutional Animal Welfare Officer (Tierschutzbeauftragter), and necessary licenses were obtained from the regional Ethical Commission for Animal Experimentation of Dresden, Germany (Tierversuchskommission, Landesdirektion Dresden), with reference numbers TVV49/2017 and TVV15/2018.

#### Bile duct ligation

Bile duct ligation was performed as described before (Tag *et al*, 2015). In brief, the abdomen was opened with midline laparotomy. The common bile duct was exposed and ligated with two surgical knots. In sham‐operated animal, the common bile duct was only exposed but not ligated. Animals were sacrificed 24 h after surgery.

#### Tissue collection and fixation

For RNA isolation and cryosections, a tissue piece of anaesthetized mice was collected and immediately frozen in liquid nitrogen and stored at −80°C.

For immunofluorescence, anaesthetized mice were perfused transcardially with 4% PFA/0.1% Tween‐20/PBS. The tissue was post‐fixed in 4% PFA/0.1% Tween‐20/PBS overnight at 4°C and neutralized in 50 mM Na4Cl/PBS for 24 h at 4°C. Tissue was stored in PBS at 4°C.

For electron microscopy, anaesthetized mice were perfused transcardially with 4% PFA/PBS, pH 7.4. The caudal lobe was resected into small cubes with a scalpel and post‐fixed in 1% GA/200 mM HEPES, pH 7.4, for 24 h at room temperature. Tissue was stored in 1% GA/200 mM HEPES, pH 7.4, at 4°C.

#### Primary hepatocyte isolation and culture

Cells were isolated as described previously (Zeigerer *et al*, 2017). In brief, primary hepatocytes were isolated from C57BL/6JOlaHsd 8‐ to 12‐week‐old male mice via collagenase perfusion. Cells were seeded with a density of 200,000 cells per well in collagen‐coated 24‐well plates. For immunofluorescence, the wells contained collagen‐coated glass cover slips. Cells were kept in Williams E medium with 10% FBS, 100 nM dexamethasone and penicillin/streptomycin at 37°C with 5% CO2. Three hours after cell seeding, cultures were coated with a second collagen layer. In every experiment, the hepatocytes were treated on day 4 after seeding with medium containing 200 μM DCA in DMSO or only DMSO and 200 μM phloretin or 1 μM ouabain for 16 h.

#### AlamarBlue cell viability assay

For the cell viability assay, cells were cultured and treated as described above. After the treatment, the culture medium of the hepatocytes was refreshed including 10% of alamarBlue Cell Viability Reagent (Thermo). Cells were incubated for 2 h at 37°C with 5% CO_2_. The medium was distributed into 96‐well plates and fluorescence was measured in a plate reader (PerkinElmer EnVision) according to the manufacturer's instructions. Background fluorescence was subtracted from 10% alamarBlue in medium incubated in wells without cells. Each biological replicate represents data from an individual mouse. For each replicate, mean values of four wells (technical replicates) are reported.

#### Immunofluorescence and confocal imaging of primary hepatocytes

Cells were fixed with 4% PFA, permeabilized with 0.1% Triton X‐100 and blocked in 10% horse serum. For Sox9 antibody staining, permeabilization was performed with 0.5% Triton X‐100. Cells were incubated with primary antibodies at 4°C overnight in 5% horse serum. To enable antibody penetration, small holes were inserted into the top layer collagen. Cells were extensively washed with 300 mM NaCl/0.1% Tween/10 mM Tris–HCl. Secondary antibodies were incubated at 4°C overnight. Thereafter, cells were washed and mounted onto glass slides with Mowiol. Imaging was performed with a Zeiss LSM 880 microscope using a Zeiss LD LCI Plan‐Apochromat 63×/1.2 DIC immersion‐corrected objective. For high‐resolution imaging, we used airyscan imaging of stacks in super‐resolution mode with 0.07 μm pixel size and 100 μm z‐step size. Airyscan stacks were processed with automated strength in 3D mode.

#### Live imaging of primary hepatocytes

Cells were seeded in 24‐well plates with glass bottom (MatTek) and cultured and treated as described above. On day 4, the culture cells were transferred to a Zeiss Celldiscoverer 7 microscope that was preheated to 37°C with 5% CO_2_. Cells were imaged with a Zeiss 50× water‐immersion objective with brightfield phase contrast. Imaging was started ~5 min after the treatment. Individual regions were imaged every 15 min.

#### Hepatocyte organoid culture

Primary hepatocytes were isolated as described above. A total of 5,000 viable hepatocytes were plated in 20 μl Matrigel (BD Bioscience) droplet and left for 1 h in incubator at 37°C with 5% CO_2_. After Matrigel polymerization, expansion media (Hu *et al*, 2018) were added. Expansion media were supplemented with 30% Wnt3a conditioned media for the establishment of primary cultures. Every 7–10 of culture, the organoids were removed from the Matrigel, mechanically dissociated with glass Pasteur pipettes and transferred to fresh Matrigel in a 1:3 split ratio.

Hepatocyte organoids were grown for ~1 month before performing the experiments. Seven days before DCA treatment, expansion media were changed to Williams E medium with 10% FBS, 100 nM dexamethasone and penicillin/streptomycin. For DCA treatment, organoids were treated with Williams E medium containing 200 μM DCA in DMSO or only DMSO for 24 h.

#### Immunofluorescence and confocal imaging of hepatocyte organoids

Organoids were removed from Matrigel by incubating in Cell Recovery Solution (Corning) for 10 min on ice. To remove remnants of Matrigel, organoids were washed carefully with cold PBS several times so as not to disrupt their 3D structure. Subsequently, organoids were fixed for 30 min on ice with 3% PFA. After fixation, organoids were washed several times with PBS. Fixed organoids were stored at 4°C until further use. Organoids were blocked with 1% bovine serum albumin (BSA) in PBS for 1 h at RT. Primary antibodies were incubated for 24 h at 4°C in 0.1% Triton X‐100 and 0.02% BSA in PBS. The secondary antibodies were added at a concentration of 1:1,000 overnight at 4°C. Nuclei were stained with DAPI (Invitrogen) at a dilution of 1:1,000 and F‐actin at a dilution of 1:200. Organoids were cleared with fructose–glycerol solution, 60% (vol/vol) glycerol and 2.5 M fructose (Dekkers *et al*, 2019). Imaging was performed with a Zeiss LSM 880 inverted microscope using a Zeiss LD LCI Plan‐Apochromat 40×/1.2 DIC immersion corrected objective. For high‐resolution imaging, we used airyscan imaging with 0.07 μm pixel size and 100 μm z‐step size in super‐resolution mode. Airyscan stacks were processed with automated strength in 3D mode.

#### RNA isolation from primary hepatocytes

RNA was isolated with TRIzol as described previously (Rio *et al*, 2010). For each biological replicate (individual mice), we performed three technical replicates. We pooled 3–4 wells per technical replicate and condition, lysed with TRIzol reagent (Invitrogen) and chloroform. After heavy centrifugation, the aqueous phase was collected and mixed 1:1 with 100% EtOH. The solution was purified with RNeasy mini kit (Qiagen). RNA concentration was measured with Nanodrop.

#### Bile canaliculi segmentation in primary hepatocytes

The bile canaliculi were reconstructed from high‐resolution (voxel size: 0.26 × 0.26 × 0.3 μm) fluorescent image stacks (~3 μm depth) of fixed primary hepatocytes stained with specific antibodies and small fluorescent molecules. Each replicate represents primary hepatocytes from an individual mouse. For each replicate, images of three wells and three to five images per well were acquired. The segmentation was performed using a FIJI (Schindelin *et al*, 2012) script (see Appendix). Briefly, saturating pixels were removed through simple thresholding. Next, simple thresholding was applied to the channel signals. Then, the channels were segmented independently with the default ImageJ method.

**Figure EV4:**
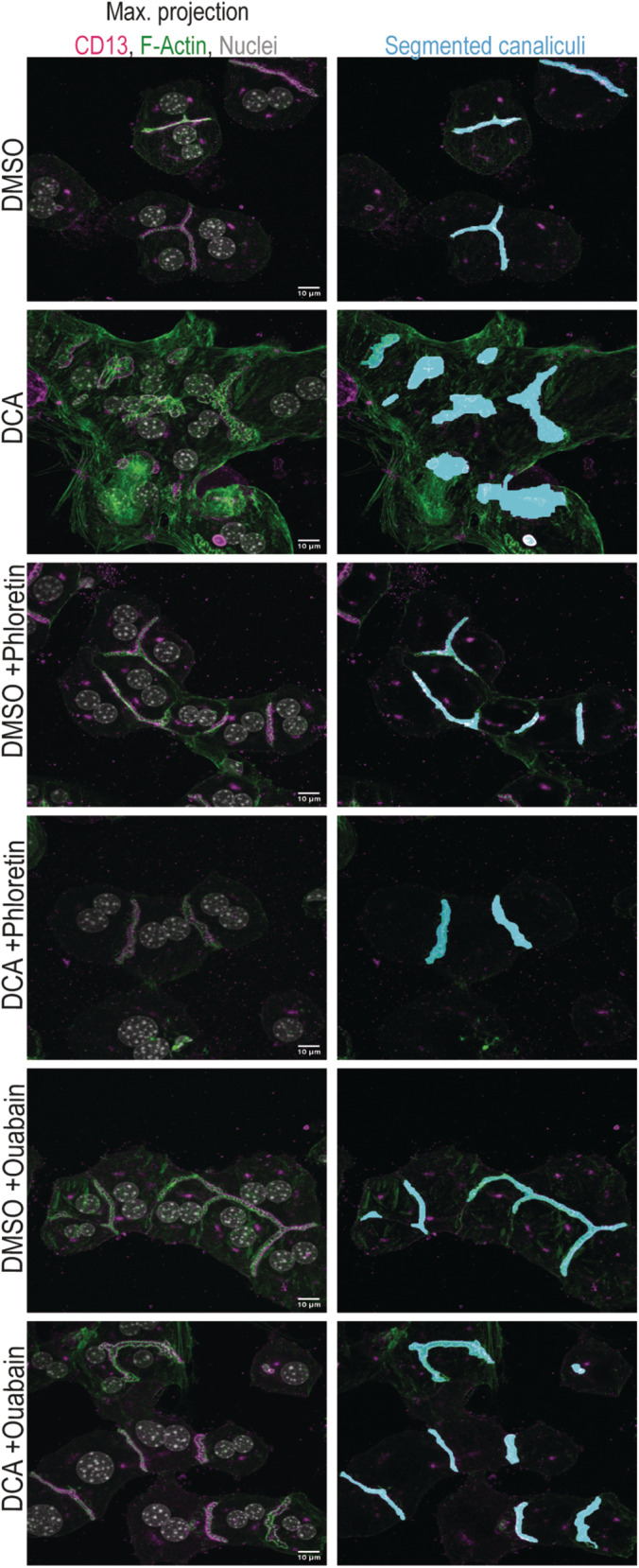
Representative images of the bile canaliculi segmentation pipeline used in Figs 6E and 7C Images are acquired as z‐stack and a maximum projection of all channels is created (first column). After the image processing pipeline, the segmented canaliculi are depicted in cyan. Representative images are shown, partially from Fig 7B. Scale bar 10 μm.

For the DCA experiment, segmentation was performed on CD13 and F‐actin staining with phalloidin. Here, a different thresholding method for DMSO and DCA conditions had to be used. For CD13, the Huang method was used (Huang & Wang, 1995), and for Phalloidin, the percentile method was used (Doyle, 1962). For all conditions, a series of closing and filling holes was then applied to the intersection of the segmented images. The result was finally used for quantifications with MorpholibJ (Legland *et al*, 2016). Examples of this segmentation pipeline can be found in Fig EV4A.

For the TCA experiment, segmentation was performed on DPPIV, CD13 and F‐actin staining with phalloidin. Here, the same thresholding method for control and TCA conditions could be used. For thresholding of DPPIV and phalloidin, the default method was used, and for CD13, the percentile method was used.

Rosette‐like canaliculi were quantified manually and defined as canaliculi with a diameter of ≥6 μm at the largest point and a reduced number of apical bulkheads.

#### Immunofluorescence and confocal imaging of murine and human liver tissue

Tissue was cut into 50 um slices, permeabilized in 0.5% Triton X‐100/PBS, quenched with 10 mM Na4Cl/PBS and blocked with 0.2% gelatin/300 mM NaCl/0.3% Triton X‐100/PBS. Primary antibodies were incubated at 4°C overnight and washed with 0.3% Triton X‐100/PBS. Secondary antibodies, phalloidin and DAPI, were incubated at room temperature for 1 h. Tissue was mounted on glass slides using Mowiol (Calbiochem). For Sox9 antibody staining, permeabilization was performed with 1% Triton X‐100/PBS.

ZO‐1 immunofluorescence was performed on cryosections. Snap‐frozen tissue was embedded into Tissue‐Tek O.C.T. (Sakura) and cut into 16‐μm‐thick sections. Methanol fixation was performed at −20°C for 10 min. Tissue was quenched with 10 mM NH4Cl/PBS, permeabilized with 0.5% Triton X‐100/ and blocked with 0.2% gelatin/300 mM NaCl/0.3% Triton X‐100/PBS. Primary antibody was applied for 2 h at room temperature and washed with 0.3% Triton X‐100/PBS. Secondary antibody, phalloidin and DAPI were added for 1 h at room temperature. Tissue was mounted with Mowiol (Calbiochem) on glass slides.

Imaging was performed with a Zeiss LSM 880 microscope using a Zeiss LD LCI Plan‐Apochromat 63×/1.2 DIC immersion‐corrected objective. For airyscan, imaging of stacks super‐resolution mode was applied and images were processed with automated strength in 3D mode.

#### RNA isolation from tissue

RNA from tissue was isolated using RNeasy mini kit (Qiagen) following the manufacturer's instructions.

#### Quantitative real‐time PCR

cDNA was produced using ProtoScript II First Strand cDNA Synthesis Kit (Biolabs) following the manufacturer's instructions. qPCR was performed using SYBR Green Mix (Thermo Scientific) according to the manufacturer's instructions. Used primers can be found in the Reagents and Tools Table. Reactions were run on Roche LightCycler using the following condition: 95°C for 15 min, 40 cycles of 95°C for 15 s, 60°C for 15 s and 72°C for 15 s. The quantification cycle (Cq) was extracted and the relative expression of each gene was calculated using the comparative Cq method normalized to GAPDH expression. Each replicate represents primary hepatocytes/tissue from an individual mouse.

#### Transmission EM and 3D reconstruction

For resin embedding, samples were post‐fixed with 1% osmiumtetroxide prepared in 1.5% potassium ferricyanide/dH2O for 1 h on ice and contrasted *en bloc* with 2% aqueous uranyl acetate for 2 h at room temperature. Dehydrating was performed with an ethanol series 50–70–80–90–96–100–100%, each for minimum of 15 min, followed by acetone, 2 × 30 min. Tissue was progressively infiltrated with EPON resin and polymerized at 70°C for 2 days.

Serial 80‐nm‐thin sections were cut using a Leica UC7 ultramicrotome deposited on formvar‐coated, slot, copper grids and contrasted with saturated aqueous uranyl acetate for 10 min, followed by 0.403% lead nitrate for 3 min. Sections were imaged in a Tecnai G2 Spirit BioTWIN transmission electron microscope (FEI, now Thermo Fisher Scientific), operated at 80 kV and equipped with a LaB6 filament, an Eagle 4 k × 4 k CCD camera and a TIA software (both FEI, now Thermo Fischer Scientific).

Z‐stack of images of serial sections were aligned using a TrackEM2 plugin in Fiji (Cardona *et al*, 2012). Apical membranes and tight junctions (without microvilli) were manually segmented using MotionTracking (http://motiontracking.mpi‐cbg.de). The reconstruction was animated in a 3D model using Blender.

#### Human liver samples

##### Cohort 1

Human liver samples from the cohort 1 were used for qualitative imaging and 3D reconstruction (Fig 4). Control samples were collected from patients with non‐hepatobiliary malignancy where an intraoperative liver biopsy was indicated but without steatosis, inflammation, ballooning or fibrosis. PSC samples were from patients undergoing liver transplantation. All samples were derived from liver resections or explants and standardized histopathologic assessments were performed by pathologists. All human liver tissue was immersion fixed in 4% PFA. Consent was approved by institutional review boards, namely by the ethics committee of the medical faculty at Universität Kiel (D425/07, A111/99).

The baseline characteristics for the cohort 1 are the following: For the control patients, three of three patients are female with a mean age of 64 years (± 15 years). For the PSC patients, two of four are female with a mean age of 47 years (± 17 years).

##### Cohort 2

Human liver samples from the cohort 2 were used for qualitative imaging and qualitative rosette analysis (Fig 5). All early‐ and late‐stage PSC and ALD, samples were obtained from liver resections/explants and standardized histopathologic assessments were performed by pathologists. PSC patients were classified into early‐ and late‐stage PSC patients based on the pathologist's description in the routine histology report from the explant liver. Late‐stage PSC samples had severe fibrotic/cirrhotic whereas early‐stage PSC samples had inflammation and only mild fibrosis. All human liver tissue was immersion fixed in 4% PFA. Consent was approved by the regional committees for medical and health research ethics of South‐East Norway (2012–286).

The baseline characteristics are the following: For early‐stage PSC patients, two of three patients are male with a mean age of 58 years (± 9 years). For late‐stage PSC patients, four of six patients are male; for two patients the gender was not reported. The average age is 47 years (± 13 years). For the end‐stage ALD patients, five of six patients are male with an average age of 57 years (± 8 years).

#### Deep imaging and 3D reconstruction

Deep imaging and 3D reconstruction were performed as described elsewhere (Morales‐Navarrete *et al*, 2015; Segovia‐Miranda *et al*, 2019). In brief, 100 μm tissue slices were cut on a vibratome, antigen retrieval was performed with citrate buffer, pH 6.0 at 80°C, and permabilization was done with 0.5% Triton X‐100/PBS. Primary antibody and secondary antibody with DAPI were incubated in 0.2% gelatin/ 300 mM NaCl/ 0.3% Triton X‐100/PBS for 2 days at room temperature and washed with 0.3% Triton X‐100/PBS. Optical clearing was done with fructose and mounted in seeDB on glass slides.

Liver samples were imaged with 0.3 μm voxel size on Zeiss LSM 780 NLO multiphoton laser‐scanning microscope using a Zeiss LD LCI Plan‐Apochromat 63×/1.2 DIC immersion corrected objective.

The BC network was imaged with CD13 immunostaining. CV areas were labelled with gluthamine synthase (GS) immunostaining. To image entire CV‐PV axes, tiles of 2 × 2 or 3 × 1 image stacks were stitched using the image stitching plug‐in of ImageJ/FIJI. For control livers one CV‐PV axis per patient was imaged and for PSC livers three different CV‐PV axis were imaged per patient. 3D reconstruction was performed using MotionTracking (http://motiontracking.mpi‐cbg.de). Thresholds were individually adjusted based on staining intensity. BC triangle mesh was selected by inflation 10. Central lines were calculated in ImageJ/FIJI and imported into MotionTracking. Fake end branches were removed using minimum end‐branch length of 10 μm. CV‐PV axis and chi map were identified and created based on GS and DAPI. Statistics were calculated with a max radius of 5 μm.

#### Rosette quantification from 3D reconstruction

Rosettes were defined as bile canaliculi segments with diameter > 6 μm. The volume of the rosettes was estimated by

$$V_{\text{canaliculi}}=\sum_{j=3}^{8}\frac{4}{3}\pi {\left(j+j_{\mathrm{off}}\right)}^{3}N_{j}$$

Where *N*_*j*_ is the number of rosettes with a radius between *j* μm and (*j* + 1) μm given for *j* = 3, 4, …, 8 μm. Here, *j*_*off*_ = 0.2 μm and reflects the fact that the rosettes with a radius between *j* μm and (*j* + 1) μm are obviously on average larger than *j* μm. The mean number of rosettes in NC and PSC patients across the CV‐PV axis are reported with standard deviation.

The total canaliculi network volume occupied by rosettes was estimated by dividing the rosette volume by the total canaliculi network volume in each area across the CV‐PV axis. Mean values for NC and PSC patients across the CV‐PV axis are reported with standard deviation.

#### Rosette quantification on tissue sections

Rosettes were manually quantified and defined as bile canaliculi segments with a diameter of > 6 μm and formed by > 2 hepatocytes. A rosette was counted Sox9+ positive if one or more hepatocytes had a Sox9 nuclear intensity of > 200 (value based on Sox9 intensity in bile duct cell nuclei). A rosette was counted pan‐CK+ if one or more hepatocytes had a cellular pan‐CK intensity of > 1,000 (value based on cellular pan‐CK intensity in bile duct cells).

#### Lobule radius measurement

Lobule measurement was performed as described in Segovia‐Miranda *et al* (2019). In brief, the tissue was processed, stained for the central vein hepatocyte marker glutamine synthetase, phalloidin and DAPI and imaged as in “Immunofluorescence and confocal imaging of murine and human liver tissue.” The full tissue pieces were imaged with a Zeiss 20× Plan Apochromat with 1.4 μm pixel size. CV‐PV axes were identified and measured manually using following criteria: (i) Each fully visible CV‐PV axis was measured, and (ii) measurements were made from the closest edges of the veins. A median lobule radius was calculated for each patient; mean lobule radius is reported for NC and PSC overall. We analysed in total *N* = 5 control patients and *N* = 4 PSC patients.

#### Statistical analysis

For the spatially resolved quantifications, one CV‐PV axis of the mean values with standard deviation of three individual NC patients and three CV‐PV axes of four individual PSC patients were plotted. Statistical analysis was performed using a paired, two‐tailed *t*‐test. For other quantifications, an unpaired, two‐tailed *t*‐test was performed. Simple linear regression analysis was performed using GraphPad Prism.

#### Canalicular bile pressure model

We consider the 3D liver lobule to be of cylindrical shape with radial symmetry and radius *L*. For each PSC patients, the radius *L* was specifically measured, whereas for the NC patients, the median value of several NC patients was used (see “Lobule radius measurements”). Let us denote *ρ*, the radial variable. Following our derivation of the biliary pressure model (Segovia‐Miranda *et al*, 2019), we account for mass conservation for water and osmolytes along with Darcy's Law and obtain nonlinear coupled first‐order differential equations for the radial bulk velocity *w*, the canaliculi pressure *p* and the osmolyte concentration *c*:

$${\left(\mathrm{\rho w}\right)}^{\prime }/\rho =\mathrm{\kappa A}\left(\rho \right)\left[\mathrm{RT}\left(c-c_{0}\right)-p\right]$$

$${\left(\mathrm{\rho cw}\right)}^{\prime }/\rho =g\left(\rho \right)$$

$$p^{\prime }=-K\left(\rho \right)w$$

Here, *A* denotes the density of apical surface, *κ* the water permeability of the membrane, *R* the gas constant, *c*_0_ the osmolyte concentration of the hepatocytes and *g* the secretion rate of osmolytes.

The boundary conditions are $w\left(\rho_{0}\right)=0$ and $p\left(L\right)=p_{L}$, where *ρ*_0_ is the radius of the central vein and *p*_*L*_ is the hydrostatic pressure of the bile duct. The canaliculus velocity can be obtained from the bulk velocity by the formula $w_{c}=w/\varepsilon_{\mathrm{BC}}$, where $\varepsilon_{\mathrm{BC}}$ is the volume fraction of the canaliculi.

The equation for the concentration can be readily integrated as follows:

$$\mathrm{\rho cw}=\int_{\rho_{0}}^{\rho }\tilde{\rho }g\left(\overset{}{\rho }\right)d\tilde{\rho }.$$

Let us denote the integral expression by *G*. Elimination of the concentration in the equation for *w* yields

$$w^{\prime }=\mathrm{\kappa A}\left[\mathrm{RT}\left(G/\left(\mathrm{\rho w}\right)-c_{0}\right)-p\right]-w/\rho .$$

The equations for *w* and *p* are then solved using a shooting method.

The proportionality constant *K* in Darcy's Law is determined from porous media theory as follows

$$K\left(\rho \right)=\frac{8\mu \tau^{2}}{\varepsilon_{\mathrm{BC}}\left(\rho \right)r_{\mathrm{BC}}{\left(\rho \right)}^{2}f\left(\rho \right)}.$$

Here, *μ* = 9.2 × 10^−4^ Pa s is the bile viscosity, *τ* is the tortuosity of the canaliculi, $r_{\mathrm{BC}}\left(\rho \right)$ is the effective canaliculus radius and *f* is a function that represents the focusing of the bile canaliculi towards the periportal field and *κ* = 4.7 × 10^−10^ m/(Pa s) and *c*_*0*_ = 300 mmol/l, all identical as in Segovia‐Miranda *et al*, 2019 (Segovia‐Miranda *et al*, 2019).

For the normal control cases, the same parameter values were used as described before (Segovia‐Miranda *et al*, 2019). It is to be noted that there is no fitting involved and all parameters are determined either from literature values or here quantified from microscopy images.

For the PSC cases, the portal boundary condition for the pressure *ρ*(*L*) was chosen to be 2,000 Pa (as opposed to 1,000 Pa in the normal control case) to reflect the fact that strictures in the bile duct build up pressure along the bile duct and likely lead to a larger pressure at the liver lobule. Furthermore, differently from the normal control cases, we did not perform tortuosity measurements or free lumen measurements for the PSC cases but assumed a canaliculi tortuosity of 1.8 and a free lumen ratio of 0.28 which are close to the normal control measurements.

Our source code for computing the pressure profiles from the above model can be found at https://github.com/MichaelKuecken/bileflow.

## Author contributions

**Carlotta Mayer:** Conceptualization; resources; data curation; formal analysis; validation; investigation; visualization; methodology; writing – original draft; project administration; writing – review and editing. **Sophie Nehring:** Investigation; methodology. **Michael Kücken:** Investigation; methodology; writing – review and editing. **Urska Repnik:** Formal analysis; investigation; methodology; writing – review and editing. **Sarah Seifert:** Investigation; methodology. **Aleksandra Sljukic:** Investigation; methodology; writing – review and editing. **Julien Delpierre:** Software; methodology. **Hernán Morales‐Navarrete:** Software; methodology; writing – review and editing. **Sebastian Hinz:** Resources. **Mario Brosch:** Resources. **Brian Chung:** Resources; writing – review and editing. **Tom Karlsen:** Resources; writing – review and editing. **Meritxell Huch:** Methodology; writing – review and editing. **Yannis Kalaidzidis:** Conceptualization; software; investigation; methodology; writing – review and editing. **Lutz Brusch:** Software; investigation; methodology; writing – review and editing. **Jochen Hampe:** Conceptualization; funding acquisition; writing – review and editing. **Clemens Schafmayer:** Resources. **Marino Zerial:** Conceptualization; resources; supervision; funding acquisition; writing – original draft; writing – review and editing.

## Disclosure and competing interests statement

The authors declare that they have no conflict of interest.

## Supporting information



Appendix

Expanded View Figures PDF

Movie EV1

Movie EV2

Movie EV3

Movie EV4

Movie EV5

Movie EV6

Movie EV7

Movie EV8

PDF+

Source Data for Figure 1

Source Data for Figure 2

Source Data for Figure 3

Source Data for Figure 4

Source Data for Figure 5

## Data Availability

Source data are available at BioImage Archive with the accession number S‐BIAD720. The bile flow model is accessible at https://github.com/MichaelKuecken/bileflow.
