# Supplementary material for: Apical bulkheads accumulate as adaptive response to impaired bile flow in liver disease
Source: EMBO Rep. 2023 Jul 31;24(9):e57181. doi: 10.15252/embr.202357181 (PMC10481669; doi:10.15252/embr.202357181)
Supplement: Supplementary file 1 — Appendix [file EMBR-24-e57181-s014.pdf]

Appendix

Table of content

Fiji script for bile canaliculi segmentation 2

Fiji script for bile canaliculi segmentation

```
# @ String (visibility = MESSAGE, 
value="<html>##############################################################
############################<br/>__________________________<b> Folder 
location and files specifications
</b>________________________________<br/>##################################
########################################################</html>") docmsg1
# @ File    (label = "Input directory", style = "directory") srcFile
# @ File    (label = "Output directory", style = "directory") dstFile
# @ String  (label = "File extension", value=".tif") ext
# @ Boolean (label = "Open with bioformat" , value=True) bifoboo
# @ Boolean (label = "Convert stack to hyperstack" , value=True) convboo
# @ Integer (label = "Nb of Channels (if convertion is needed)", value="4")
nch
# @ Integer (label = "Nb of Z Slices (if convertion is needed)", 
value="16") nsl
# @ String (label = "Voxel size x/y (in µm)", value="0.3") xysiz
# @ String (label = "Voxel size z (in µm)", value="0.3") zsiz
# @ String (visibility = MESSAGE, 
value="<html>##############################################################
############################<br/>________________________________<b> 
Segmentation parameters
</b>___________________________________<br/>###############################
###########################################################</html>") 
docmsg2
# @ String (visibility = MESSAGE, value="<html><b>Remove saturating 
pixels</b></html>") docmsga
# @ String  (label = "List of channels to clean (format: 2,3 / to skip: 
0)", value="2,3") dirtlist
# @ Integer (label = "Maximum intensity (per pixel value, for cleaning)", 
style="slider", min=0, max=65500, stepSize=1000, value=50000) dirtthre
# @ String (visibility = MESSAGE, value="<html><b>Apical 
Surfaces</b></html>") docmsgz
# @ Integer (label = "Apical Surface Marker Channel (0 if none)", 
value="2") chcd
# @ Integer (label = "Apical Surface signal smoothing (in pixels for median 
smooth)", style="slider", min=0, max=10, stepSize=1, value=2) cdsmo
# @ String (label = "Apical Surface segmentation method", choices = 
{"Default", "Huang", "Huang2", "Intermodes", "IsoData", "Li", "MaxEntropy", 
"Mean", "MinError(I)", "Minimum", "Moments", "Otsu", "Percentile", 
"RenyiEntropy", "Shanbhag", "Triangle", "Yen"}, style="listBox") CD13met
# @ Integer (label = "Minimal size for surface seeds (in voxels)", 
style="slider", min=0, max=2000, stepSize=50, value=50) siz0
# @ String (visibility = MESSAGE, value="<html><b>Tight 
Junctions</b></html>") docmsge
# @ Integer (label = "Junctions Marker Channel (0 if none)", value="3") 
chju
# @ Integer (label = "Junctions Marker signal smoothing (in pixels for 
median smooth)", style="slider", min=0, max=10, stepSize=1, value=2) jusmo
# @ String (label = "Junctions segmentation method", choices = {"Default", 
"Huang", "Huang2", "Intermodes", "IsoData", "Li", "MaxEntropy", "Mean", 
"MinError(I)", "Minimum", "Moments", "Otsu", "Percentile", "RenyiEntropy", 
"Shanbhag", "Triangle", "Yen"}, style="listBox") Zomet
# @ Integer (label = "Minimal size for junction seeds (in voxels)", 
style="slider", min=0, max=2000, stepSize=50, value=50) siz2
# @ String (visibility = MESSAGE, value="<html><b>Actin</b></html>") 
docmsgr
# @ Integer (label = "Phalloidin Channel (0 if none)", value="1") chph
# @ Integer (label = "Phalloidin signal smoothing (in pixels for median 
smooth)", style="slider", min=0, max=10, stepSize=1, value=5) phsmo
```

```
# @ String (label = "Phalloidin segmentation method", choices = {"Default",
"Huang", "Huang2", "Intermodes", "IsoData", "Li", "MaxEntropy", "Mean",
"MinError(I)", "Minimum", "Moments", "Otsu", "Percentile", "RenyiEntropy",
"Shanbhag", "Triangle", "Yen"}, style="listBox") Phalomet
# @ String (visibility = MESSAGE, value="<html><b>Lumina</b></html>")
docmsgt
# @ Integer (label = "Minimal size for Lumina (in voxels)", style="slider",
min=0, max=2000, stepSize=50, value=200) siz1
# @ Integer (label = "Maximum diameter for Lumina (in µm)", style="slider",
min=0, max=100, stepSize=10, value=30) scadia
# @ Boolean (label = "Remove Lumina on borders" , value=True) borderboo
# @ Boolean (label = "Fill Holes" , value=True) fillboo
# @ Integer (label = "Closing cycles", style="slider", min=0, max=50,
stepSize=1, value=10) cloc
# @ String (visibility = MESSAGE,
value="<html>############################################################
###########################<br/>_______________________________________<b>
Output
</b>__________________________________________<br/>#####################
###########################################################</html>
") docmsg3
# @ Boolean (label = "MorpholibJ" , value=True) morphoboo
# @ Boolean (label = "Local thickness" , value=True) localboo
# @ Boolean (label = "Skeleton analysis" , value=True) squeboo
# @ Boolean (label = "Export binary mask of lumina" , value=True) Binboo
# @ Boolean (label = "MaxProjection output" , value=True) Maxboo

import os
from ij import IJ, ImagePlus, ImageStack
from ij.plugin import ImageCalculator
from ij import WindowManager
from ij import process as proc
import datetime as dt

ic = ImageCalculator()
w = WindowManager

####Interpret user input
dirtboo = True
if dirtlist == '0':
	dirtboo = False
dirtlist = list(dirtlist.split(','))
dirtlist = [int(a) for a in dirtlist]

borde= ''
if borderboo:
	borde = 'exclude_objects_on_edges'

cdboo=True
if chcd == 0:
	cdboo=False

juboo=True
if chju == 0:
	juboo=False

phboo=True
if chph == 0:
	phboo=False

rgsta = "slices"
if Maxboo:
```

```
        rgsta = ''
######

srcDir = srcFile.getAbsolutePath()
dstDir = dstFile.getAbsolutePath()
files = [a for a in os.listdir(srcDir) if a.endswith(ext)]

def run():
        for fil in files:
                if bifoboo:
                        IJ.run("Bio-Formats Importer",
"open="+os.path.join(srcDir, fil) +" autoscale color_mode=Default
view=Hyperstack stack_order=XYCZT")
                        process(fil, nch, nsl)
                if not bifoboo:
                        IJ.open(os.path.join(srcDir, fil))
                        process(fil, nch, nsl)
        logger()

##########################Helper Functions
def sourcimage(nch, nsl):
        imp = IJ.getImage()
        if convboo:
                nsli = imp.getNSlices()
                nfra = nsl
                if nsl != nsli and nsl>1 :
                        IJ.run("Stack to
Hyperstack...","order=xyczt(default) channels="+str(nch)+"
slices="+str(nsl)+" frames=1 display=Color")
                imp = IJ.getImage()
        if not convboo:
                nfra = imp.getNSlices()
                nch = imp.getNChannels()
        calib = imp.getCalibration()
        calib.setUnit("um")
        nfra = imp.getDimensions()[3]
        IJ.run("Properties...", "channels="+str(nch)+" slices="+str(nfra)+"
frames=1 pixel_width="+xysiz+" pixel_height="+xysiz+" voxel_depth="+zsiz)
        imp = IJ.getImage()
        imp.setTitle("source")

#Get Maximal pixel value and associated slice from a stack
def getmxint(imp, nsl):
        slt = 0
        intt = 0
        for i in range(1,nsl+1):
                IJ.setSlice(i)
                ip = imp.getProcessor().convertToFloat()
                pixels = ip.getPixels()
                mx = max(pixels)
                if mx > intt:
                        intt = mx
                        slt = i
        return intt, slt

#Remove saturating pixels and surrounding area
def RemoveDirt(cibl, cha, sortie, nch, nsl):
        IJ.selectWindow(cibl)
        if dirtboo:
                nampara = ''
                for q in range(nch):
                        l = q+1
```

```
                        nampara = nampara+'c'+str(l)+'=Masked'+str(l)+' '
                        if l in cha:
                                IJ.selectWindow(cibl)
                                IJ.run("Duplicate...", "duplicate
channels="+str(l))
                                imp = IJ.getImage()
                                imp.setTitle("Source"+str(l))
                                imp = IJ.getImage()
                                IJ.run("Duplicate...", "duplicate
channels="+str(l))
                                imp = IJ.getImage()
                                imp.setTitle("Msk")
                                imp = IJ.getImage()
                                intt, slt = getmxint(imp, nsl)
                                if intt < dirtthre:
                                        imp.setTitle("Masked"+str(l))
                                        IJ.selectWindow("Source"+str(l))
                                        clos()
                                        continue
                                IJ.setSlice(slt)
                                IJ.setThreshold(dirtthre, 65535)
                                IJ.run("Convert to Mask", "method=Default
background=Dark")
                                for a in range(5):
                                        IJ.run("Dilate (3D)", "iso=255")
                                IJ.setThreshold(0, 1)
                                IJ.run("Convert to Mask", "method=Default
background=Dark")
                                imp = IJ.getImage()
                                IJ.run(imp, "Divide...", "value=255.000
stack")
                                imstk2 = w.getImage("Source"+str(l))
                                imstk1 = w.getImage("Msk")
                                imp3 = ic.run("multiply stack", imstk2,
imstk1)
                                IJ.selectWindow("Source"+str(l))
                                imp = IJ.getImage()
                                imp.setTitle("Masked"+str(l))
                                IJ.selectWindow("Msk")
                                clos()
                        else:
                                IJ.selectWindow(cibl)
                                IJ.run("Duplicate...", "duplicate
channels="+str(l))
                                imp = IJ.getImage()
                                imp.setTitle("Masked"+str(l))

                IJ.run("Merge Channels...", nampara+"create")
                imp = IJ.getImage()
                imp.setTitle(sortie)

        if not dirtboo:
                IJ.selectWindow(cibl)
                imp = IJ.getImage()
                imp.setTitle(sortie)

#Close modified images without saving
def clos():
        imp = IJ.getImage()
        imp.changes = False
        imp.close()
```

```
#Basic segmentation
def SimpleSeg(cibl, chan, rad, sortie, method):
        IJ.selectWindow(cibl)
        IJ.run("Duplicate...", "duplicate channels="+chan)
        IJ.run ("Median...", "radius="+rad+" stack")
        imp = IJ.getImage()
        imp.setTitle(sortie)
        IJ.run("Auto Threshold", "method="+method+" ignore_black
ignore_white white stack use_stack_histogram")

#Save the results and clear table
def saveres(fil, nam, boole):
        out = os.path.join(dstDir, fil[:-4])
        tmp = "-"+nam+".csv"
        IJ.saveAs("Results", out+tmp)
        if boole:
                IJ.renameResults(fil[:-4]+tmp, "Results")
        IJ.run("Clear Results", "")
        print("Saved: "+fil[:-4]+tmp)
        return out

#Merge Images for visualisation
def vis(imli, colli, segch, Sortie, mx):
        a=0
        tmpli=""
        ac=''
        for im in imli:
                IJ.selectWindow(im)
                IJ.run("Duplicate...", "duplicate")
                imp = IJ.getImage()
                IJ.run("16-bit")
                IJ.run(colli[a])
                a = a+1
                imp = IJ.getImage()
                imp.setTitle("co"+str(a))
                q = "c"+str(a)+"=co"+str(a)+" "
                tmpli= tmpli+q
                ac=ac+'1'
        IJ.run("Merge Channels...", tmpli+"create keep")
        if mx:
                IJ.run("Z Project...", "projection=[Max Intensity]")
        for i in segch:
                imp = IJ.getImage()
                imp.setC(i)
                IJ.setMinAndMax(0, 1)
        imp = IJ.getImage()
        imp.setActiveChannels(ac)
        imp = IJ.getImage()
        imp.setTitle(Sortie)
        a=1
        for im in imli:
                IJ.selectWindow("co"+str(a))
                clos()
                a=a+1

def addupndown(imag, nsl):
        IJ.selectWindow(imag)
        IJ.setSlice(nsl)
        IJ.run("Add Slice", "add=slice")
        IJ.setSlice(1)
        IJ.run("Add Slice", "add=slice prepend")
```

```
#Remove background based on gaussian blur
def pretreat(cibl, chan, sortie):
#background
	bck = "BckMask"+str(chan)
	fore = "ForMask"+str(chan)
	IJ.selectWindow(cibl)
	IJ.run("Duplicate...", "duplicate channels="+chan)
	imp = IJ.getImage()
	imp.setTitle(bck)
	IJ.run("Gaussian Blur...", "sigma=4 scaled stack"),
#Foreground
	IJ.selectWindow(cibl)
	IJ.run("Duplicate...", "duplicate channels="+chan)
	imp = IJ.getImage()
	imp.setTitle(fore)
	IJ.run ("Median...", "radius=3 stack")
#Pre-treatment
	imstk2 = w.getImage(bck)
	imstk1 = w.getImage(fore)
	imp3 = ic.run("substract stack", imstk1, imstk2)
	imp = IJ.getImage()
	imp.setTitle(sortie)
	IJ.selectWindow(bck)
	clos()

#Generate a logfile
def logger():
	now = str(dt.date.today())+' at '+str((dt.datetime.utcnow() +
dt.timedelta(hours=1)).time())
	clocboo = True
	if cloc == 0:
		clocboo = False

	logstext='Logs from the analysis done the '+now+'''
	\nList of operations:\n  Dirt Removal:  '''+str(dirtboo)+'\n
Apical surface segmentation:  '+str(cdboo)+'\n  Phalloidin segmentation:
'+str(phboo)+'\n  Junctions segmentation:  '+str(juboo)+'\n  Closing:
'+str(clocboo)+'''
	\nOutput provided:\n  MorpholibJ:  '''+str(morphoboo)+'\n
LocalThickness:  '+str(localboo)+'\n  Skeleton Analysis:
'+str(squeboo)+'\n  Binary mask of lumina:  '+str(Binboo)+'\n
MaxProjection for the output:  '+str(Maxboo)+'''
	\nParameters:\n  List of channels to clean:  '''+str(dirtlist)+'\n
Upper threshold for cleaning:  '+str(dirtthre)+'\n\n  Apical surface marker
channel:  '+str(chcd)+'\n  Apical Surface signal smoothing:
'+str(cdsmo)+'\n  Apical surface segmentation method:  '+CD13met+'\n
Minimal size for surface seeds:  '+str(siz0)+'''
	\n  Junctions marker channel:  '''+str(chju)+'\n  Junctions marker
signal smoothing:  '+str(jusmo)+'\n  Junctions segmentation method:
'+Zomet+'\n  Minimal size for junctions seeds:  '+str(siz2)+'''
	\n  Phalloidin channel:  '''+str(chph)+'\n  Phalloidin signal
smoothing:  '+str(phsmo)+'\n  Phalloidin segmentation method:
'+Phalomet+'''
	\n  Minimal size for Lumina:  '''+str(siz1)+'\n  Maximum diameter
for Lumina:  '+str(scadia)+'\n  Remove Lumina on borders:
'+str(borderboo)+'\n  Closing cycles:  '+str(cloc)+'''
	\nFile parameters:\n  Voxel size x/y:  '''+xysiz+'\n  Voxel size z:
'+zsiz+'\n  File extension:  '+ext+'\n  Bioformat:  '+str(bifoboo)+'\n
Convertion to Hyperstack:  '+str(convboo)+'\n  Number of Channels:
'+str(nch)+'\n  Number of Z slices:  '+str(nsl)+'''
	\nInput Directory:  '''+str(srcDir)+'\nOutput Directory:
'+str(dstDir)+'\n\nFiles Processed:'
```

```
        for a in files:
                logstext = logstext+'\n  '+str(a)

        with open(os.path.join(dstDir, 'Logs.txt'), 'w') as a:
                a.write(logstext)

####################Main###############
def process(fil, nch, nsl):

        sourcimage(nch, nsl)
        IJ.selectWindow('source')
        imp = IJ.getImage()
        nch = imp.getNChannels()
        nsl = imp.getNSlices()

        #Remove saturating pixels
        RemoveDirt('source', dirtlist, "clean", nch, nsl)

        #Segmentation on CD13 and Phalloidin
        if cdboo:
                SimpleSeg("clean", str(chcd), str(cdsmo), "CD13seg",
CD13met)
        if phboo:
                SimpleSeg("clean", str(chph), str(phsmo), "Phalloseg",
Phalomet)

        #Get the overlap between segmented images
        if cdboo and phboo:
                IJ.selectWindow("CD13seg")
                IJ.run("Duplicate...", "duplicate")
                imstk1 = w.getImage("CD13seg")
                imstk2 = w.getImage("Phalloseg")
                imp3 = ic.run("AND stack", imstk1, imstk2)
                IJ.selectWindow("CD13seg")
        imp = IJ.getImage()
        imp.setTitle("Seg0")

        #First estimation of canaliculi
        addupndown("Seg0", nsl)
        IJ.run("3D OC Options", "surface nb_of_surf._voxels centroid
centre_of_mass bounding_box dots_size=5 font_size=10
store_results_within_a_table_named_after_the_image_(macro_friendly)")
        IJ.run("3D Objects Counter", "threshold=1 slice=1
min.="+str(siz0)+" max.=999999999 "+borde+" objects statistics")
        IJ.renameResults("Statistics for Seg0", "Results")
        IJ.run("Clear Results", "")
        imp = IJ.getImage()
        imp.setTitle("Estim")

        IJ.selectWindow("Seg0")
        clos()
        IJ.selectWindow("Estim")

        #Connect and fill
        IJ.run("Fill Holes (Binary/Gray)")
        IJ.setThreshold(1, 255)
        IJ.run("Convert to Mask", "method=Default background=Default")
        for i in range(cloc):
                IJ.run("Dilate", "stack")
        if fillboo:
                IJ.run("Fill Holes", "stack")
```

```
        for i in range(cloc):
                IJ.run("Erode", "stack")
        if fillboo:
                IJ.run("3D Fill Holes")
        for i in range(cloc):
                IJ.run("Open", "stack")
        imp = IJ.getImage()
        imp.setTitle("Lumina0")

        IJ.run("3D Objects Counter", "threshold=1 slice=1
min.="+str(siz1)+" max.=999999999 "+borde+" objects statistics")
        IJ.renameResults("Statistics for Lumina0", "Results")
        IJ.run("Clear Results", "")
        imp = IJ.getImage()
        imp.setTitle("Lumina0obj")

        IJ.selectWindow("Estim")
        clos()
        IJ.selectWindow("Lumina0")
        clos()
        IJ.selectWindow("Lumina0obj")

        #Get junctions as seed and sort lumina
        if juboo:
                pretreat("clean", str(chju), "PreTreatedJunctions")
                SimpleSeg("PreTreatedJunctions", '1', str(jusmo), "ZO1seg",
Zomet)
                addupndown("ZO1seg", nsl)
                IJ.run("3D Objects Counter", "threshold=8 slice=8
min.="+str(siz2)+" max.=999999999 objects")
                IJ.setThreshold(1, 255)
                IJ.run("Convert to Mask", "method=Default background=Dark")
                IJ.run("Close-", "stack")
                imp = IJ.getImage()
                imp.setTitle("ZO1seeds")
                IJ.run("Morphological Reconstruction 3D", "marker=ZO1seeds
mask=Lumina0obj type=[By Dilation] connectivity=6")

        #Get local thickness
        IJ.setThreshold(1, 65535)
        IJ.run("Convert to Mask", "method=Default background=Default")
        imp = IJ.getImage()
        imp.setTitle("LuminaMsk")

        locatic = ''
        if localboo:
                IJ.run("Conversions...", " ")
                IJ.run("Local Thickness (complete process)", "threshold=1")
                IJ.setMinAndMax(0, scadia)
                imp = IJ.getImage()
                imp.setTitle("LocalThick")
                IJ.run("Duplicate...", "duplicate")
                IJ.run("16-bit")
                locatic='redirect_to=LocalThick-1'

        #Measure and save
        IJ.run("3D OC Options", "surface nb_of_obj._voxels
nb_of_surf._voxels integrated_density mean_gray_value std_dev_gray_value
median_gray_value minimum_gray_value maximum_gray_value centroid
centre_of_mass bounding_box dots_size=5 font_size=10
store_results_within_a_table_named_after_the_image_(macro_friendly)
"+locatic)
```

```
        IJ.selectWindow("LuminaMsk")
        IJ.run("16-bit")
        IJ.run("3D Objects Counter", "threshold=1 slice=1
min.="+str(siz1)+" max.=10048576 "+borde+" objects statistics")
        out = saveres(fil, 'ObjectCounter', True)
        imp = IJ.getImage()
        nfra = imp.getNSlices()
        imp.setTitle("Luminab")
        IJ.run("Duplicate...", "duplicate range=2-"+str(nfra-1))
        imp = IJ.getImage()
        imp.setTitle("Lumina")

        if localboo:
                IJ.selectWindow("LocalThick-1")
                clos()
        IJ.selectWindow("Luminab")
        clos()
        IJ.selectWindow("LuminaMsk")
        clos()
        IJ.selectWindow("Lumina")

        if morphoboo:
                IJ.run("Analyze Regions 3D", "voxel_count volume
surface_area mean_breadth sphericity euler_number bounding_box centroid
equivalent_ellipsoid ellipsoid_elongations max._inscribed
surface_area_method=[Crofton (13 dirs.)] euler_connectivity=6")
                out = saveres(fil, 'Morpho', True)

        if squeboo:
                IJ.selectWindow("Lumina")
                IJ.run("Duplicate...", "duplicate")
                IJ.run("Skeletonize (2D/3D)")
                if localboo:
                        imp = IJ.getImage()
                        imp.setTitle("Lumina-1")
                        imp = IJ.getImage()
                        IJ.run(imp, "Divide...", "value=255.000 stack")
                        IJ.selectWindow("LocalThick")
                        IJ.run("Duplicate...", "duplicate range=2-
"+str(nfra-1))
                        imp = IJ.getImage()
                        IJ.run("8-bit")
                        imstk1 = w.getImage("Lumina-1")
                        imstk2 = w.getImage("LocalThick-1")
                        imp3 = ic.run("Multiply stack", imstk1, imstk2)
                        IJ.selectWindow("Lumina-1")
                IJ.run("Analyze Skeleton (2D/3D)", "prune=none show
display")
                IJ.selectWindow("Results")
                out = saveres(fil, 'Skeleton', False)
                IJ.selectWindow("Branch information")
                out = saveres(fil, 'Branches', True)
                IJ.selectWindow('Tagged skeleton')
                if localboo:
                        IJ.selectWindow("Lumina-1")
                        IJ.setMinAndMax(0, scadia)
                IJ.run("RGB Color")
                if Maxboo:
                        IJ.run("Z Project...", "projection=[Max Intensity]")
                IJ.saveAs("tiff",  out+"-Skeleton.tif")
                clos()
                #IJ.selectWindow("Lumina-1")
```

```
            #clos()
            IJ.selectWindow("Lumina-1-labeled-skeletons")
            clos()

    #Save segmentation result
    if localboo:
            IJ.selectWindow("LocalThick")
            IJ.run("RGB Color")
            if Maxboo:
                    IJ.run("Z Project...", "projection=[Max Intensity]")
            IJ.saveAs("tiff",  out+"-LocalThickness.tif")

    if cdboo and phboo:
            a=['CD13seg-1', 'Phalloseg', 'Lumina']
            b=["Cyan","Blue","Magenta"]
            c=[1,2,3]
            vis(a, b, c, "seg", Maxboo)
            IJ.run("RGB Color", rgsta)
            IJ.saveAs("tiff",  out+"-ApicalActinandLumina.tif")
            clos()

    #Save Tight junctions lumina sorting
    if juboo:
            IJ.selectWindow("ZO1seeds")
            IJ.run("Duplicate...", "duplicate range=2-"+str(nfra-1))
            IJ.selectWindow('Lumina0obj')
            IJ.run("Duplicate...", "duplicate range=2-"+str(nfra-1))

            a=['ZO1seeds-1', 'Lumina0obj-1', 'Lumina']
            b=["Yellow","Magenta","Cyan"]
            c=[1,2,3]
            vis(a, b, c, "lum", Maxboo)
            IJ.run("RGB Color", rgsta)
            IJ.saveAs("tiff",  out+"-JunctionsSorting.tif")
            clos()

    #Save overlay for validation
    if cdboo and phboo:
            IJ.selectWindow('clean')
            IJ.run("Duplicate...", "duplicate channels="+str(chcd))
            imp = IJ.getImage()
            imp.setTitle("CD13")
            IJ.selectWindow('clean')
            IJ.run("Duplicate...", "duplicate channels="+str(chph))
            imp = IJ.getImage()
            imp.setTitle("Phallo")

            a=['CD13', 'Phallo', 'Lumina']
            b=["Magenta","Green","Cyan"]
            c=[3]
            if juboo:
                    IJ.selectWindow('clean')
                    IJ.run("Duplicate...", "duplicate
channels="+str(chju))
                    imp = IJ.getImage()
                    imp.setTitle("ZO1")
                    a.append("ZO1")
                    b.append("Yellow")

            vis(a, b, c, "Over", Maxboo)
            IJ.run("RGB Color", rgsta)
            IJ.saveAs("tiff",  out+"-OriginalImageAndLumina.tif")
```

```
                clos()

        if cdboo and not phboo:
                IJ.selectWindow('clean')
                IJ.run("Duplicate...", "duplicate channels="+str(chcd))
                imp = IJ.getImage()
                imp.setTitle("CD13")
                a=['CD13', 'Lumina']
                b=["Magenta", "Cyan"]
                c=[2]
                if juboo:
                        IJ.selectWindow('clean')
                        IJ.run("Duplicate...", "duplicate
channels="+str(chju))
                        imp = IJ.getImage()
                        imp.setTitle("ZO1")
                        a.append("ZO1")
                        b.append("Yellow")

                vis(a, b, c, "Over", Maxboo)
                IJ.run("RGB Color", rgsta)
                IJ.saveAs("tiff",  out+"-OriginalImageAndLumina.tif")
                clos()

        if phboo and not cdboo:
                IJ.selectWindow('clean')
                IJ.run("Duplicate...", "duplicate channels="+str(chph))
                imp = IJ.getImage()
                imp.setTitle("Phallo")
                a=['Phallo', 'Lumina']
                b=["Green", "Cyan"]
                c=[2]
                if juboo:
                        IJ.selectWindow('clean')
                        IJ.run("Duplicate...", "duplicate
channels="+str(chju))
                        imp = IJ.getImage()
                        imp.setTitle("ZO1")
                        a.append("ZO1")
                        b.append("Yellow")
                vis(a, b, c, "Over", Maxboo)
                IJ.run("RGB Color", rgsta)
                IJ.saveAs("tiff",  out+"-OriginalImageAndLumina.tif")
                clos()

        if Binboo:
                IJ.selectWindow('Lumina')
                IJ.run("Duplicate...", "duplicate")
                IJ.saveAs("tiff",  out+"-Binary.tif")
                clos()

        IJ.run("Close All", "")

run()
```
