# Supplementary material for: Apical bulkheads accumulate as adaptive response to impaired bile flow in liver disease
Source: EMBO Rep. 2023 Jul 31;24(9):e57181. doi: 10.15252/embr.202357181 (PMC10481669; doi:10.15252/embr.202357181)
Supplement: Supplementary file 2 — Expanded View Figures PDF [file EMBR-24-e57181-s010.pdf]

Expanded View Figures

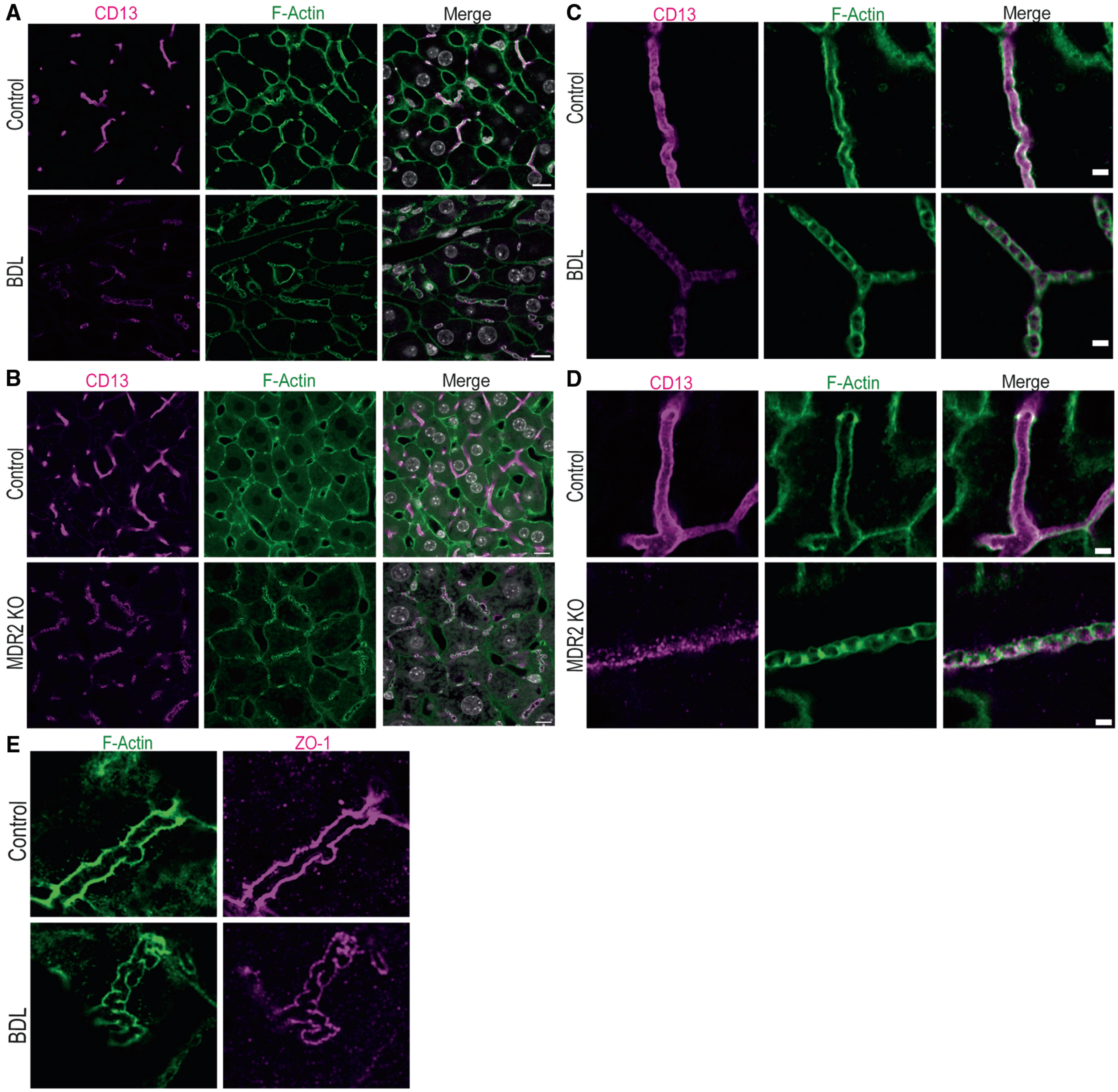

Figure EV1.

Figure EV1. F-Actin co-localizes with the canalicular makers CD13 and ZO-1 in bile canaliculi.

A Overview images of murine liver tissue after control surgery or bile duct ligation (BDL) for 24 h ($N = 3$ biological replicates). Immunofluorescence from Fig 1A stained for CD13 (magenta), F-actin (green) and nuclei (grey). Scale bar 10 μm.

B Overview images of murine liver tissue of control and MDR2 KO mice after 12 weeks ($N = 3$ biological replicates). Immunofluorescence from Fig 1C stained for CD13 (magenta), F-actin (green) and nuclei (grey). Scale bar 10 μm.

C High-resolution imaging of individual bile canaliculi in murine liver tissue after control and BDL surgery from Fig 1B. Immunofluorescence for CD13 (magenta) and F-actin (green). Scale bar 2 μm.

D High-resolution imaging of individual bile canaliculi in murine liver tissue of control and MDR2 KO mice after 12 weeks (N = 3 biological replicates) from Fig 1D. Immunofluorescence for CD13 (magenta) and F-actin (green). Scale bar 2 μm.

E High-resolution imaging of individual bile canaliculi in murine liver tissue after control and BDL surgery for 24 h from Fig 1G. Immunofluorescence for ZO-1 (magenta) and F-actin (green). Scale bar 2 μm.

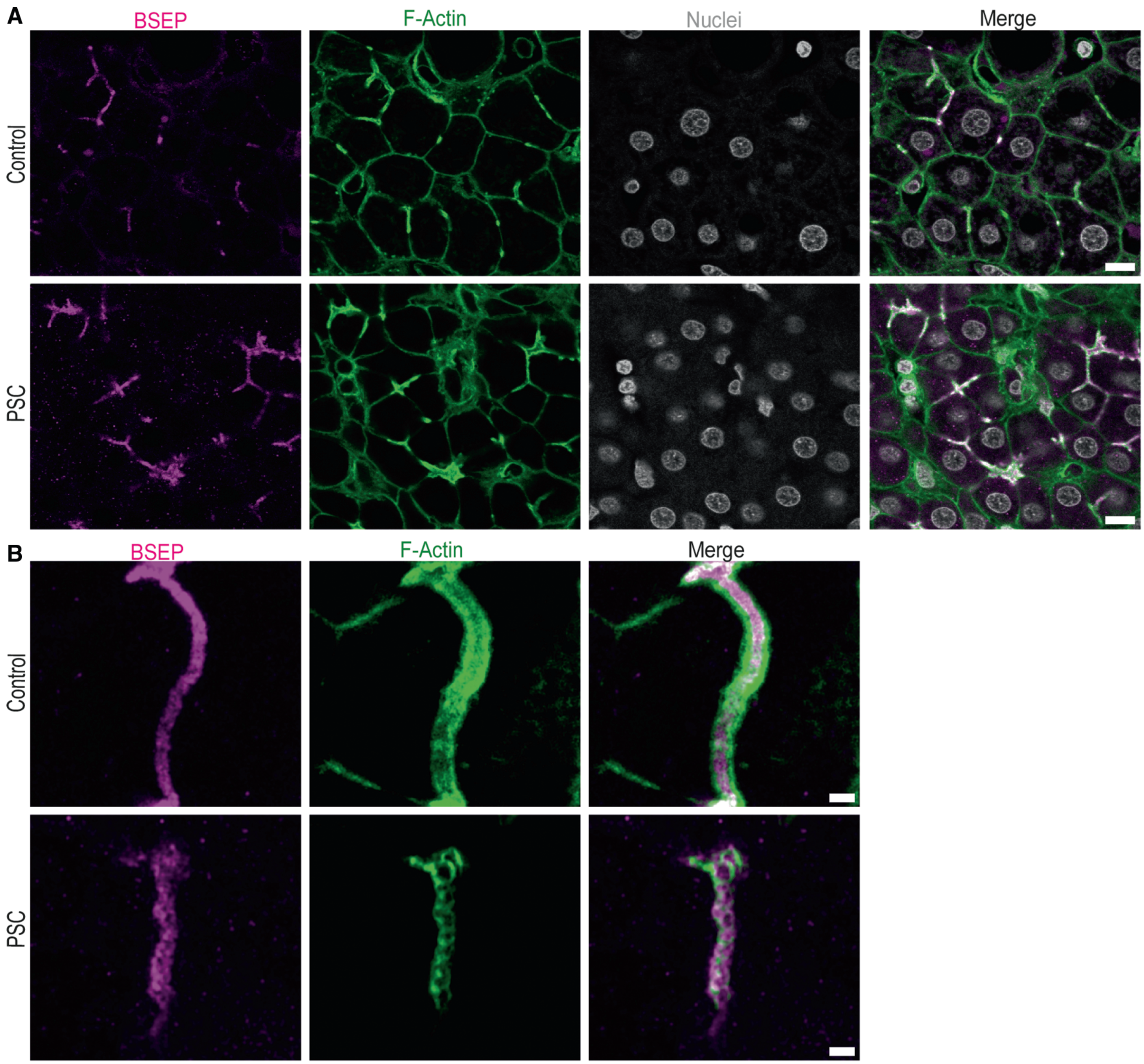

Figure EV2.

© 2023 The Authors

Figure EV2. F-Actin co-localizes with the canalicular marker BSEP in bile canaliculi.

A Overview images in liver tissue of human control and PSC patients from Fig 2A. Individual channels of immunofluoresence for the apical membrane marker BSEP (magenta), F-actin (green) and nuclei (white). Scale bar 10 μm.

B High-resolution imaging of individual bile canaliculi in control and PSC patients from Fig 2B. Immunofluorescence for BSEP (magenta) and F-actin (green). Scale bar 2 μm.

Figure EV3. *In vitro* systems upon DCA and TCA treatments.

A Primary hepatocytes treated with DMSO or 200 μM DCA for 16 h from Fig 6A. Individual channels of immunofluorescence for CD13 (magenta), F-actin (green) and nuclei (grey). Scale bar 10 μm.

B High-resolution microscopy images of individual bile canaliculi in primary hepatocytes treated with DMSO or 200 μM DCA from Fig 6B. Immunofluorescence for CD13 (magenta) and F-actin (green). Scale bar 2 μm.

C DCA treatment does not affect primary hepatocyte viability. Untreated, DMSO- and DCA-treated primary hepatocytes after 16 h treatment showed no changes in fluorescence intensity of alamarBlue cell viability reagent indicating no changes in cell viability ($N = 4$ biological replicates). Unpaired *t*-test.

D Primary hepatocytes treated with water or 50 μM TCA for 16 h. Individual channels of immunofluorescence for CD13 (magenta), F-actin (green) and nuclei (grey). Scale bar 10 μm.

E Quantification of the mean bile canaliculi volume in μm^3 of primary hepatocyte treated with water and 50 μM TCA ($N = 2$ biological replicates, technical replicates shown). Unpaired *t*-test $*P < 0.05$.

F–H Gene expression analysis of (F) *Abcb11*/BSEP, (G) *Cyp7a1*/Cyp7 and (H) *Sox9*/Sox9 in primary hepatocytes treated with water or 50 μM DCA. Represented are mean Cq values normalized to GAPDH expression and whiskers show min and max values ($N = 3$ biological replicates). One-sample *t*-test $**P < 0.01$.

I Hepatocyte organoids from Fig 6J with immunofluorescence for ZO-1 (yellow) and F-actin (green). Scalebar 2 μm.

Data information: Boxplots show the 25th and 75th percentiles and the central band shows the median value. Whiskers extend to the min and max values.

© 2023 The Authors

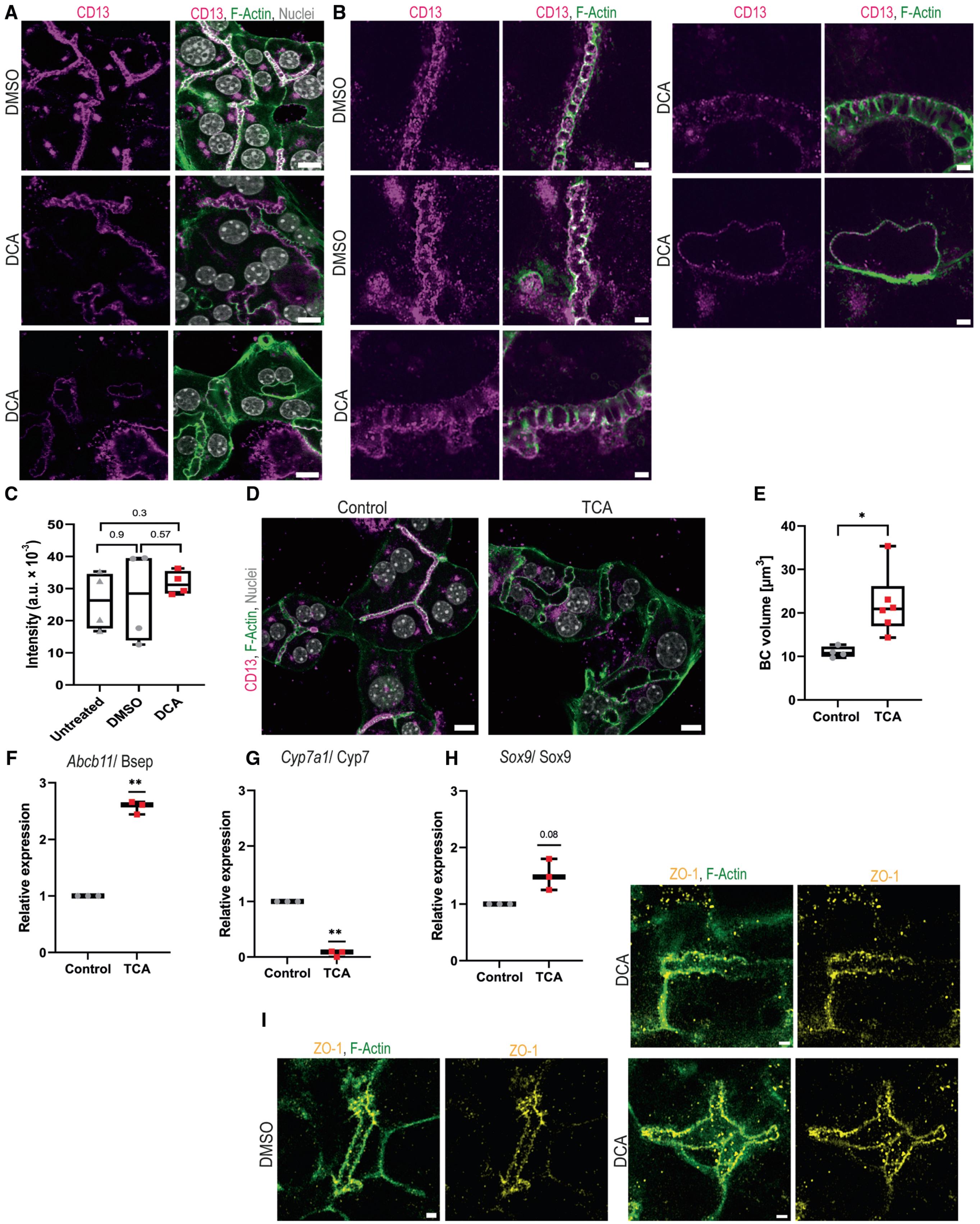

Figure EV3.

 © 2023 The Authors

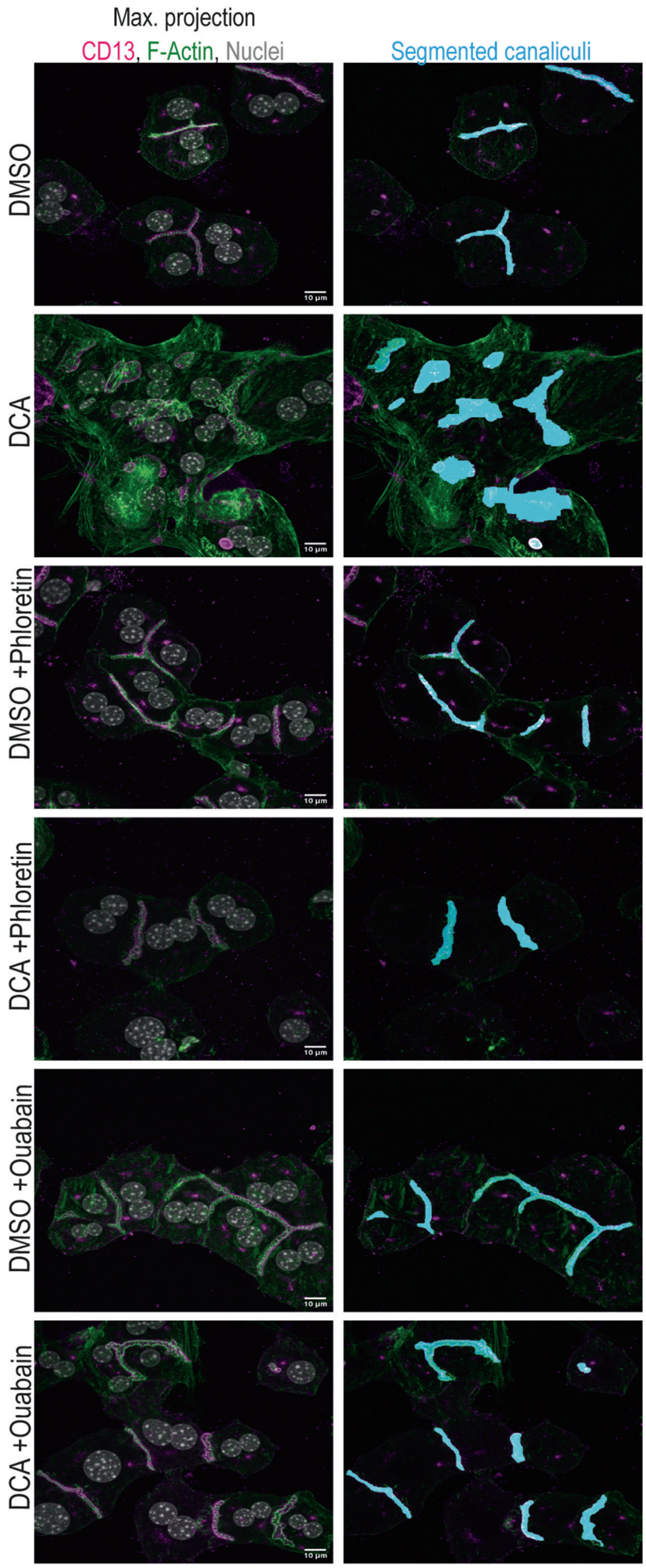

Figure EV4. Representative images of the bile canaliculi segmentation pipeline used in Figs 6E and 7C.

Images are acquired as z-stack and a maximum projection of all channels is created (first column). After the image processing pipeline, the segmented canaliculi are depicted in cyan. Representative images are shown, partially from Fig 7B. Scale bar 10 µm.

© 2023 The Authors
