# Supplementary figures and images for: Apical bulkheads accumulate as adaptive response to impaired bile flow in liver disease
Source: EMBO Rep. 2023 Jul 31;24(9):e57181. doi: 10.15252/embr.202357181 (PMC10481669; doi:10.15252/embr.202357181)

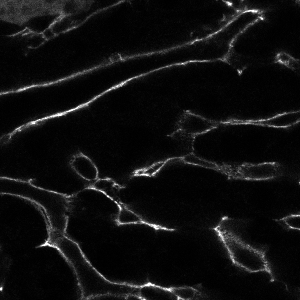

Supplement: Supplementary file 12 — Source Data for Figure 1 [file EMBR-24-e57181-s008.zip › Figure_1/1A/BDL.tif]

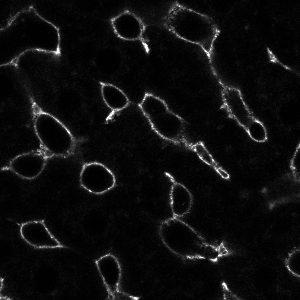

Supplement: Supplementary file 12 — Source Data for Figure 1 [file EMBR-24-e57181-s008.zip › Figure_1/1A/Control.tif]

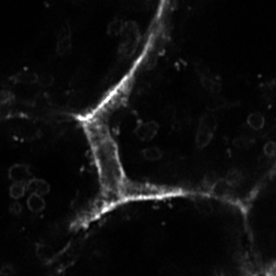

Supplement: Supplementary file 12 — Source Data for Figure 1 [file EMBR-24-e57181-s008.zip › Figure_1/1B/IF_50um_#18-1_Fibronectin_647_CD13_568_Phall_488_DAPI_Sham_PV_BC_2_Airyscan_Processing-1.tif]

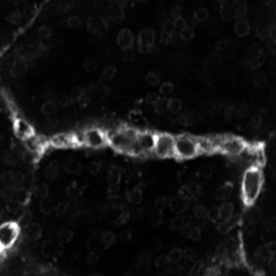

Supplement: Supplementary file 12 — Source Data for Figure 1 [file EMBR-24-e57181-s008.zip › Figure_1/1B/IF_50um_#18-2_Fibronectin_647_CD13_568_Phall_488_DAPI_BDL24h_PV_BC_1_Airyscan_Processing_Airyscan_Processing-1.tif]

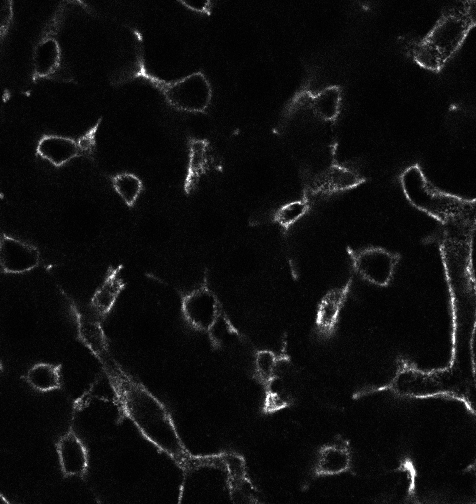

Supplement: Supplementary file 12 — Source Data for Figure 1 [file EMBR-24-e57181-s008.zip › Figure_1/1C/1_Control_647_Fibronectin_568_CD13_488_Phall_DAPI_60x_PV_1-1.tif]

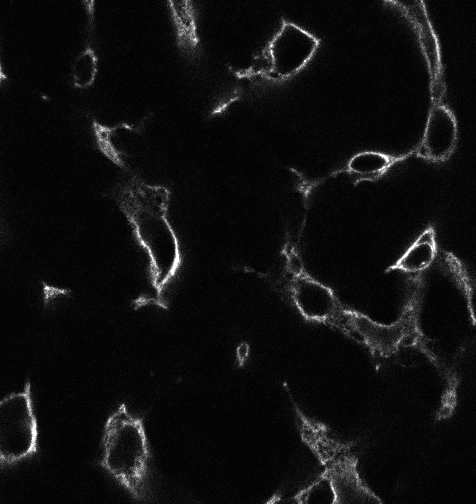

Supplement: Supplementary file 12 — Source Data for Figure 1 [file EMBR-24-e57181-s008.zip › Figure_1/1C/5_MDR2KO_647_Fibronectin_568_CD13_488_Phall_DAPI_60x_CV_1-1.tif]

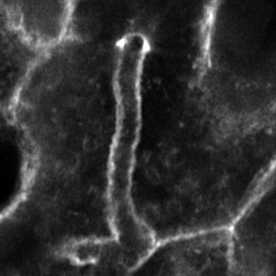

Supplement: Supplementary file 12 — Source Data for Figure 1 [file EMBR-24-e57181-s008.zip › Figure_1/1D/1_WT_A651_647_Fibronectin_568_CD13_Phall_DAPI_60x_BC_Airyscan_Processing-1.tif]

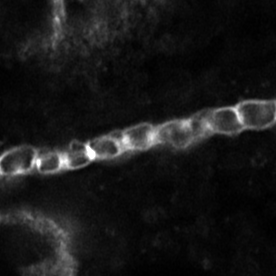

Supplement: Supplementary file 12 — Source Data for Figure 1 [file EMBR-24-e57181-s008.zip › Figure_1/1D/4_MDR2--_A994_647_Fibronectin_568_CD13_Phall_DAPI_60x_BC_Airyscan_Processing-1.tif]

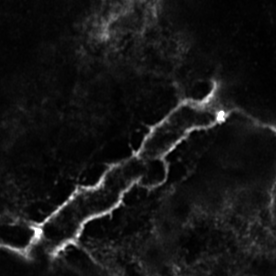

Supplement: Supplementary file 12 — Source Data for Figure 1 [file EMBR-24-e57181-s008.zip › Figure_1/1G/IF_Cryo_#4-1_ZO-1_Phall_488_DAPI_sham1d_63x_tile_1_BC_CV_1_Airyscan_Processing-1.tif]

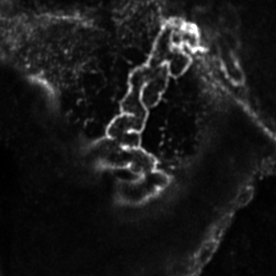

Supplement: Supplementary file 12 — Source Data for Figure 1 [file EMBR-24-e57181-s008.zip › Figure_1/1G/IF_Cryo_#4-2_ZO-1_Phall_488_DAPI_Bdl1d_63x_tile_2_BC_CV_2_Airyscan_Processing-1.tif]

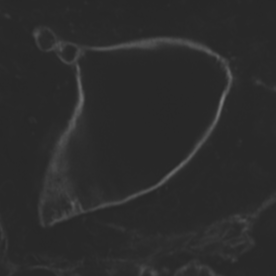

Supplement: Supplementary file 12 — Source Data for Figure 1 [file EMBR-24-e57181-s008.zip › Figure_1/1H/IF_50um_#21-1_Fibronectin_647_CD13_568_Phall_488_DAPI_BDL_Cysts_Airy_1_Airyscan_Processing-1.tif]

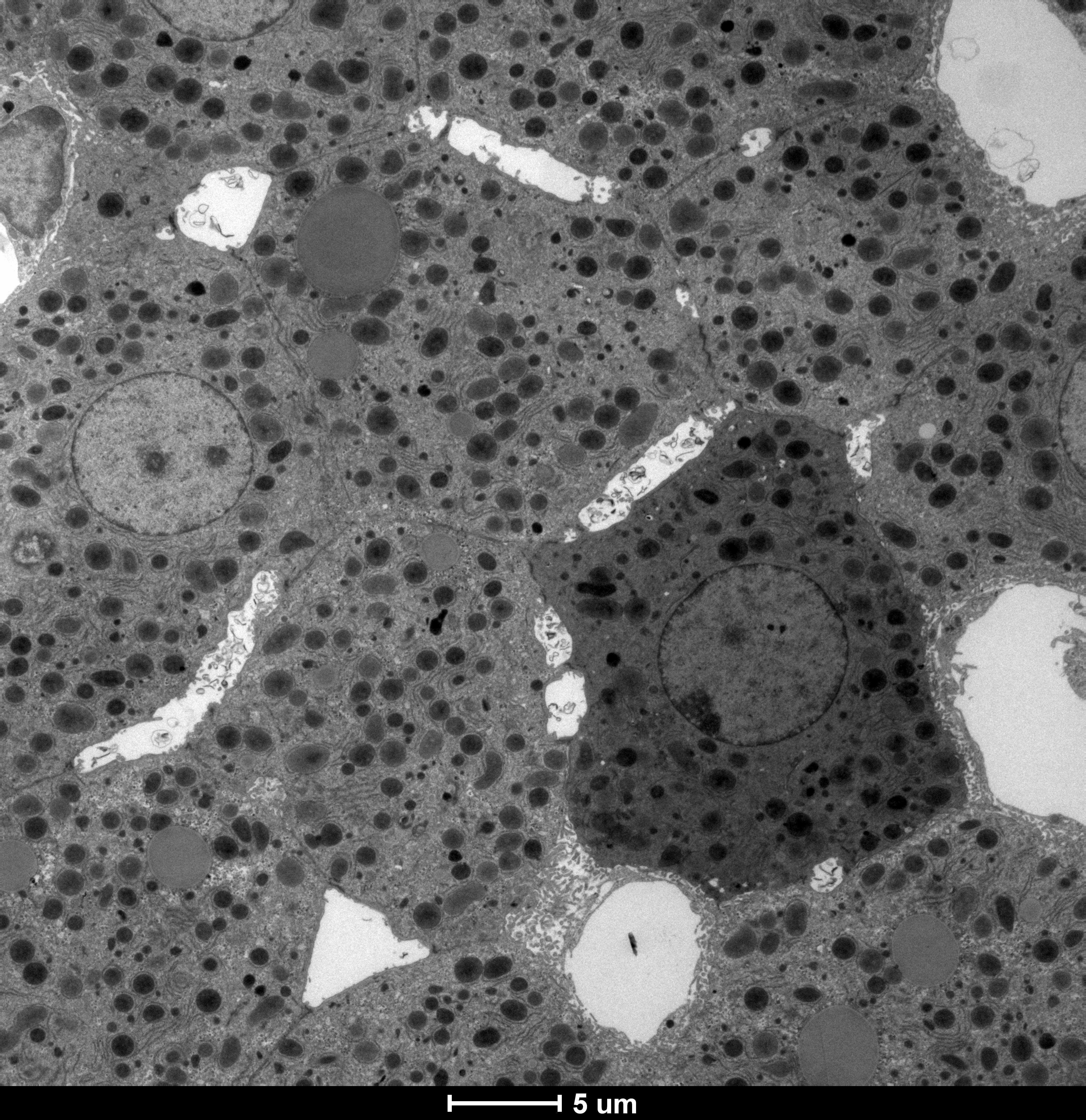

Supplement: Supplementary file 12 — Source Data for Figure 1 [file EMBR-24-e57181-s008.zip › Figure_1/1I/gb31-Q2_e2021-10-19_3d1_Zerial-Carlotta-Liver-BDL_i2022-05-11_0041.tif]

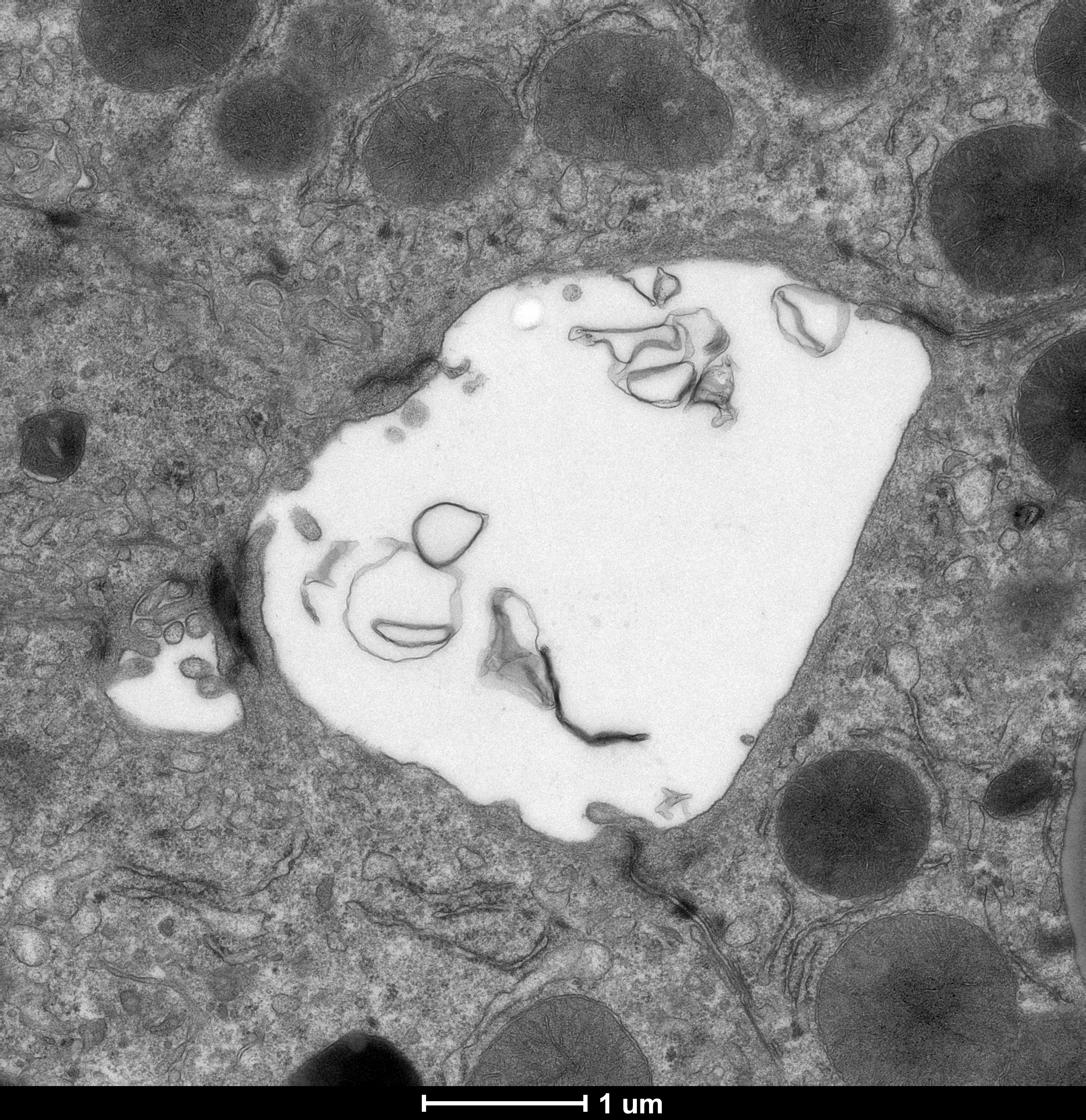

Supplement: Supplementary file 12 — Source Data for Figure 1 [file EMBR-24-e57181-s008.zip › Figure_1/1I/gb31-Q2_e2021-10-19_3d1_Zerial-Carlotta-Liver-BDL_i2022-05-11_0050.tif]

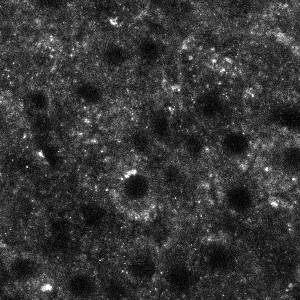

Supplement: Supplementary file 13 — Source Data for Figure 2 [file EMBR-24-e57181-s004.zip › Figure_2/2A/IF_50um_#4-2_Fibronectin_647_BSEP_568_Phall_488_DAPI_PSCpatient_PV_zoom_stack_2-1.tif]

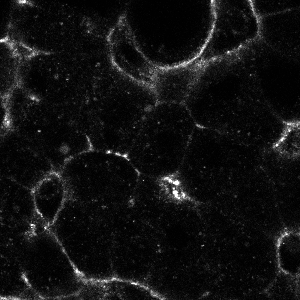

Supplement: Supplementary file 13 — Source Data for Figure 2 [file EMBR-24-e57181-s004.zip › Figure_2/2A/IF_50um_#4-7_Fibronectin_647_BSEP_568_Phall_488_DAPI_Controlpatient_PV_zoom_stack_1-1.tif]

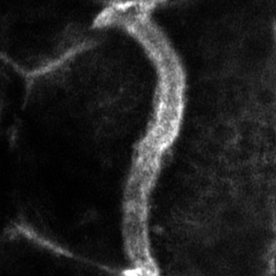

Supplement: Supplementary file 13 — Source Data for Figure 2 [file EMBR-24-e57181-s004.zip › Figure_2/2B/1_Control_647_BSEP_Phall_DAPI_60x_BC_1_Airyscan_Processing-1.tif]

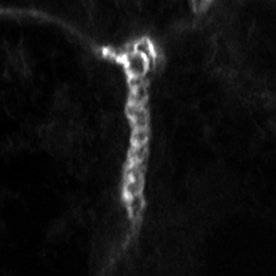

Supplement: Supplementary file 13 — Source Data for Figure 2 [file EMBR-24-e57181-s004.zip › Figure_2/2B/1_PSC_647_BSEP_Phall_DAPI_60x_BC3_Airyscan_Processing-1.tif]

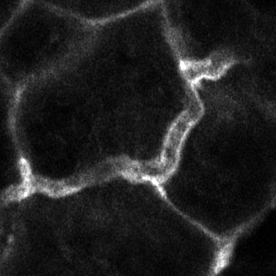

Supplement: Supplementary file 13 — Source Data for Figure 2 [file EMBR-24-e57181-s004.zip › Figure_2/2C/1_Control_647_BSEP_Phall_DAPI_60x_BC_Longt_Airy_Airyscan_Processing.tif]

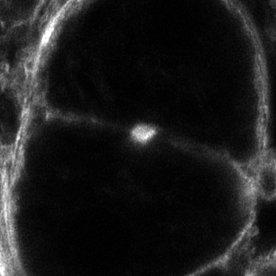

Supplement: Supplementary file 13 — Source Data for Figure 2 [file EMBR-24-e57181-s004.zip › Figure_2/2C/1_Control_647_BSEP_Phall_DAPI_60x_BC_Transv_Airy_Airyscan_Processing.tif]

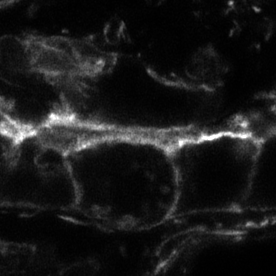

Supplement: Supplementary file 13 — Source Data for Figure 2 [file EMBR-24-e57181-s004.zip › Figure_2/2C/1_Control_647_BSEP_Phall_DAPI_60x_BD_Longt_Airy_Airyscan_Processing.tif]

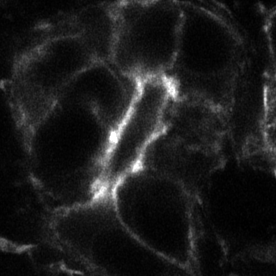

Supplement: Supplementary file 13 — Source Data for Figure 2 [file EMBR-24-e57181-s004.zip › Figure_2/2C/1_Control_647_BSEP_Phall_DAPI_60x_BD_Transv_Airy_Airyscan_Processing.tif]

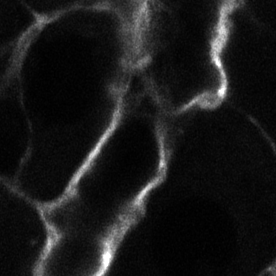

Supplement: Supplementary file 13 — Source Data for Figure 2 [file EMBR-24-e57181-s004.zip › Figure_2/2C/1_PSC_647_BSEP_Phall_DAPI_60x_Rosette_Longt_Airy_Airyscan_Processing.tif]

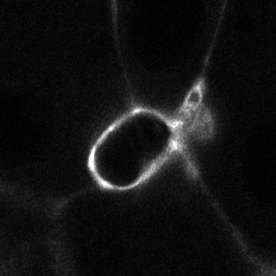

Supplement: Supplementary file 13 — Source Data for Figure 2 [file EMBR-24-e57181-s004.zip › Figure_2/2C/1_PSC_647_BSEP_Phall_DAPI_60x_Rosette_Transv_AIry_2_Airyscan_Processing.tif]

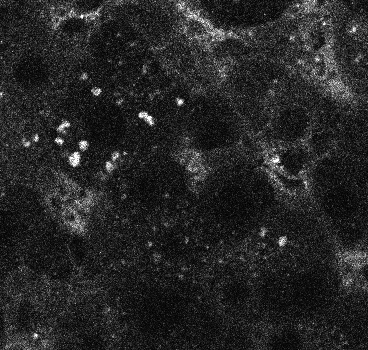

Supplement: Supplementary file 13 — Source Data for Figure 2 [file EMBR-24-e57181-s004.zip › Figure_2/2F/1_Control_Prom1_647_BSEP_555_Phall_488_DAPI_60x_F0.6_2_bsep.tif]

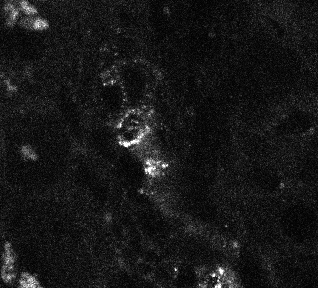

Supplement: Supplementary file 13 — Source Data for Figure 2 [file EMBR-24-e57181-s004.zip › Figure_2/2F/1_Control_Prom1_647_BSEP_555_Phall_488_DAPI_60x_F0.6_3-1.tif]

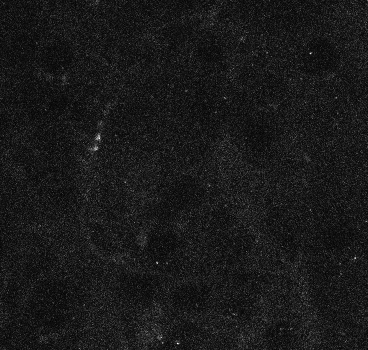

Supplement: Supplementary file 13 — Source Data for Figure 2 [file EMBR-24-e57181-s004.zip › Figure_2/2F/2_PSC_Prom1_647_BSEP_555_Phall_488_DAPI_60x_F0.6_5_bsep.tif]

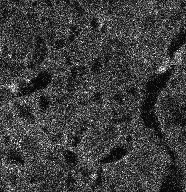

Supplement: Supplementary file 14 — Source Data for Figure 3 [file EMBR-24-e57181-s011.zip › Figure_3/3A/IF_50um_#21-6_Sox9_647_Phall_488_DAPI_PSC_parchy_1_stack-1.tif]

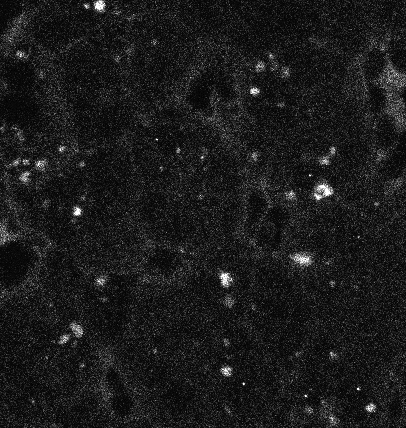

Supplement: Supplementary file 14 — Source Data for Figure 3 [file EMBR-24-e57181-s011.zip › Figure_3/3A/IF_50um_#21-7_Sox9_647_Phall_488_DAPI_Control_parchy_2-1.tif]

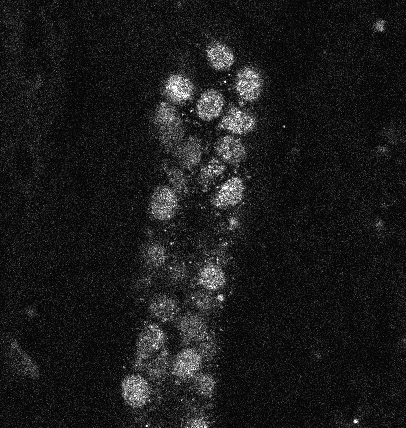

Supplement: Supplementary file 14 — Source Data for Figure 3 [file EMBR-24-e57181-s011.zip › Figure_3/3A/IF_50um_#21-7_Sox9_647_Phall_488_DAPI_Control_PV_2-1.tif]

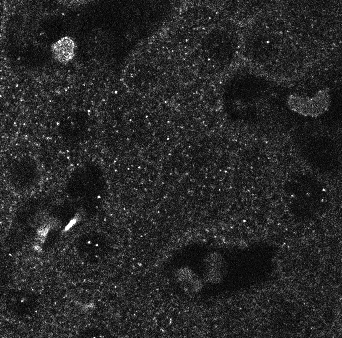

Supplement: Supplementary file 14 — Source Data for Figure 3 [file EMBR-24-e57181-s011.zip › Figure_3/3B/7_Control_PanCK_647_Phall_488_DAPI_60x_F0.6_2-1.tif]

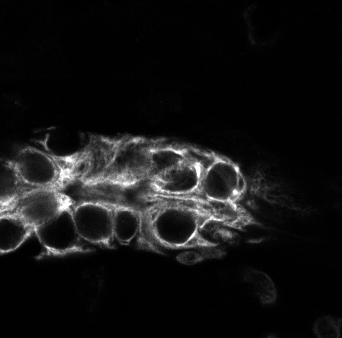

Supplement: Supplementary file 14 — Source Data for Figure 3 [file EMBR-24-e57181-s011.zip › Figure_3/3B/7_Control_PanCK_647_Phall_488_DAPI_60x_F0.6_3-1.tif]

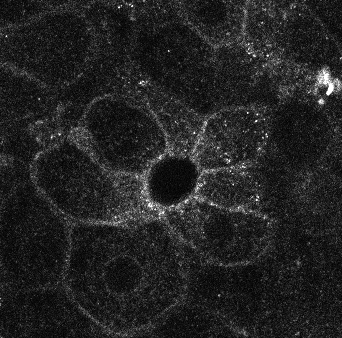

Supplement: Supplementary file 14 — Source Data for Figure 3 [file EMBR-24-e57181-s011.zip › Figure_3/3B/8_PSC_PanCK_647_Phall_488_DAPI_60x_F0.6_3-1.tif]

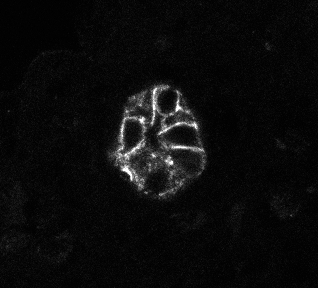

Supplement: Supplementary file 14 — Source Data for Figure 3 [file EMBR-24-e57181-s011.zip › Figure_3/3C/5_Control_Trop2_647_Phall_488_DAPI_60x_F0.6_1_Zoom_10umSB.tif]

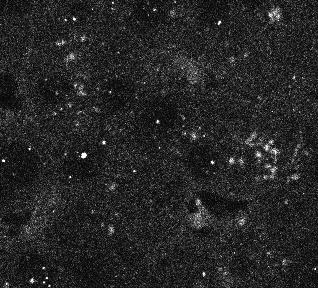

Supplement: Supplementary file 14 — Source Data for Figure 3 [file EMBR-24-e57181-s011.zip › Figure_3/3C/5_Control_Trop2_647_Phall_488_DAPI_60x_F0.6_3_zoom_10umSB.tif]

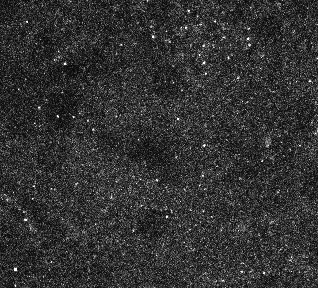

Supplement: Supplementary file 14 — Source Data for Figure 3 [file EMBR-24-e57181-s011.zip › Figure_3/3C/6_PSC_Trop2_647_Phall_488_DAPI_60x_F0.6_4_zoom_10umSB.tif]

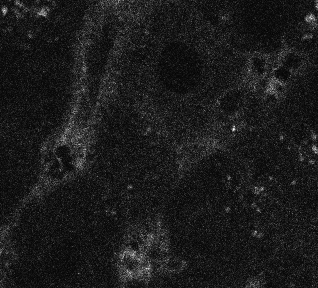

Supplement: Supplementary file 14 — Source Data for Figure 3 [file EMBR-24-e57181-s011.zip › Figure_3/3D/1_Control_Prom1_647_BSEP_555_Phall_488_DAPI_60x_F0.6_2-1.tif]

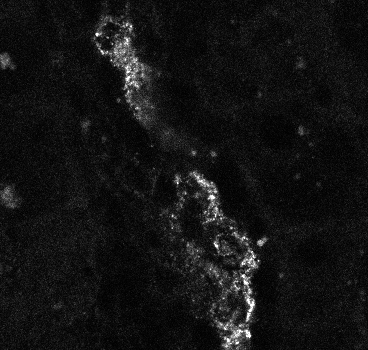

Supplement: Supplementary file 14 — Source Data for Figure 3 [file EMBR-24-e57181-s011.zip › Figure_3/3D/1_Control_Prom1_647_BSEP_555_Phall_488_DAPI_60x_F0.6_4_bsep.tif]

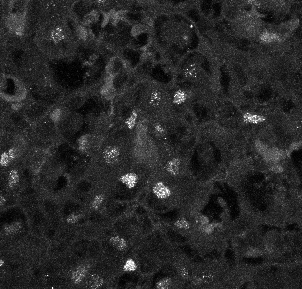

Supplement: Supplementary file 14 — Source Data for Figure 3 [file EMBR-24-e57181-s011.zip › Figure_3/3E/MAX_IF_50um_#21-6_Sox9_647_Phall_488_DAPI_PSC6890_Pseudobd_1_stack-1.tif]

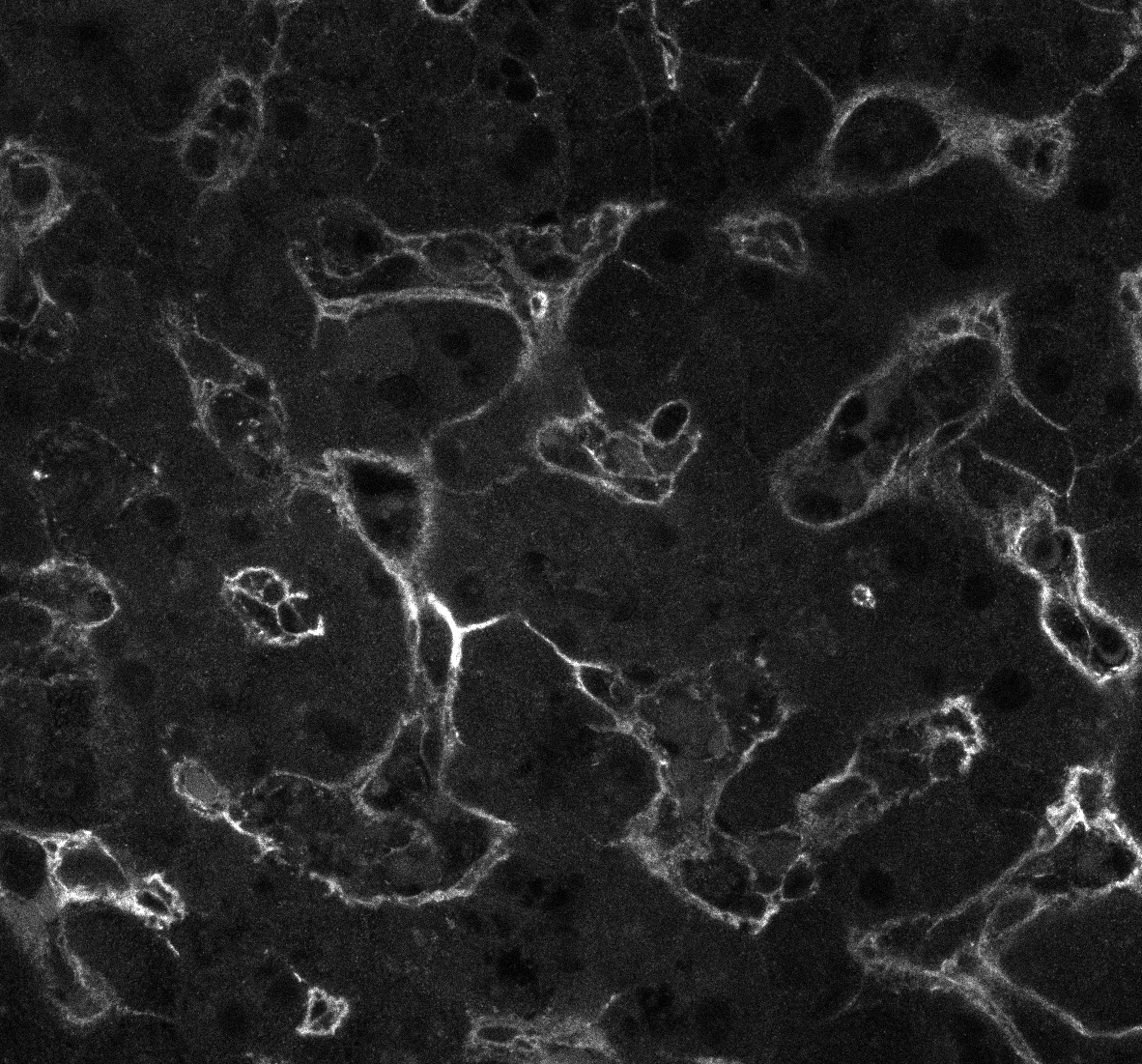

Supplement: Supplementary file 16 — Source Data for Figure 5 [file EMBR-24-e57181-s003.zip › Figure_5/5A/IF_50um_#16-2_Fibronectin_647_555_BSEP_Phall_488_DAPI_EarlyPSC_axis_1-2.tif]

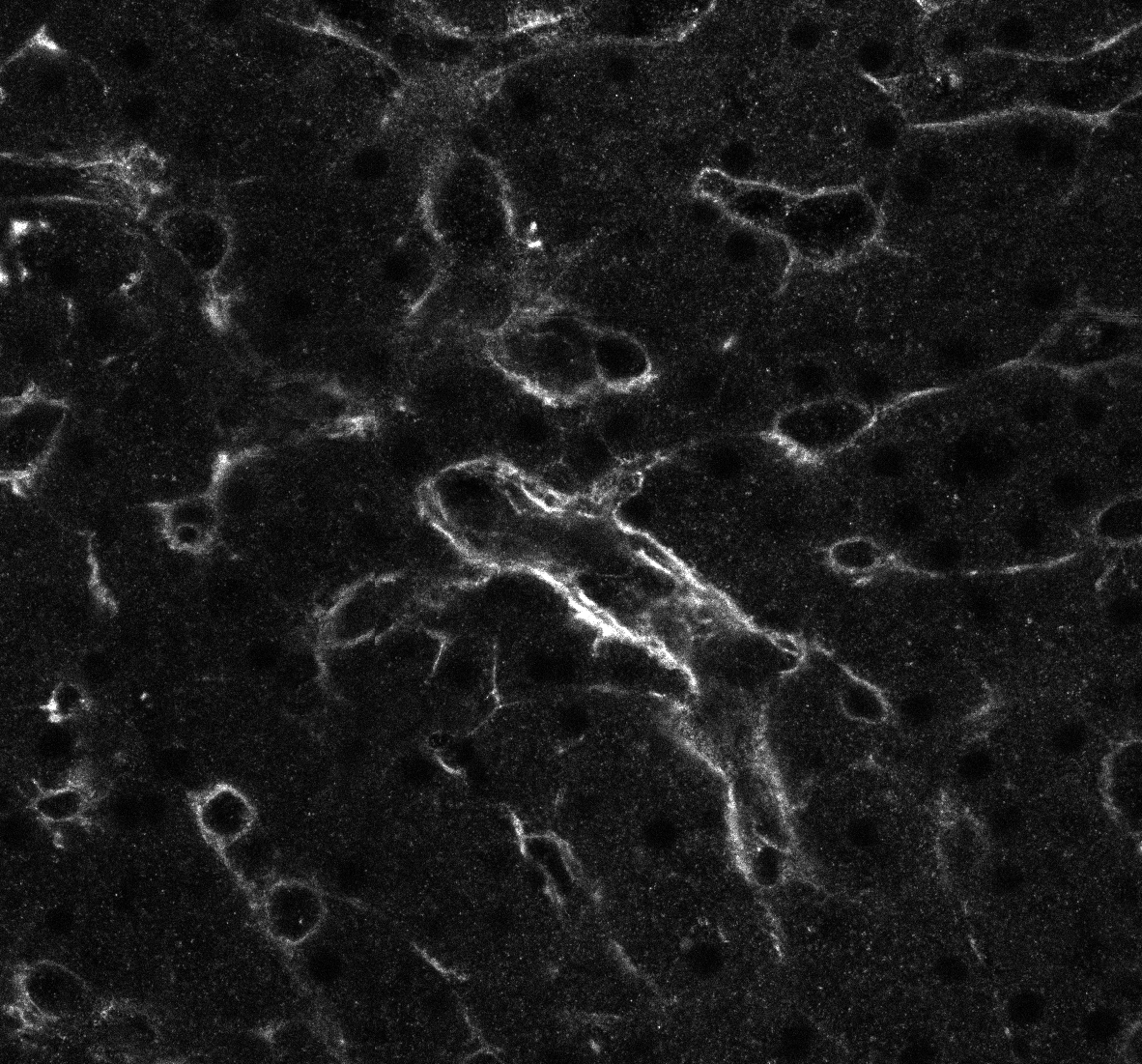

Supplement: Supplementary file 16 — Source Data for Figure 5 [file EMBR-24-e57181-s003.zip › Figure_5/5A/IF_50um_#16-5_Fibronectin_647_555_BSEP_Phall_488_DAPI_LatePSC_axis_1-1.tif]

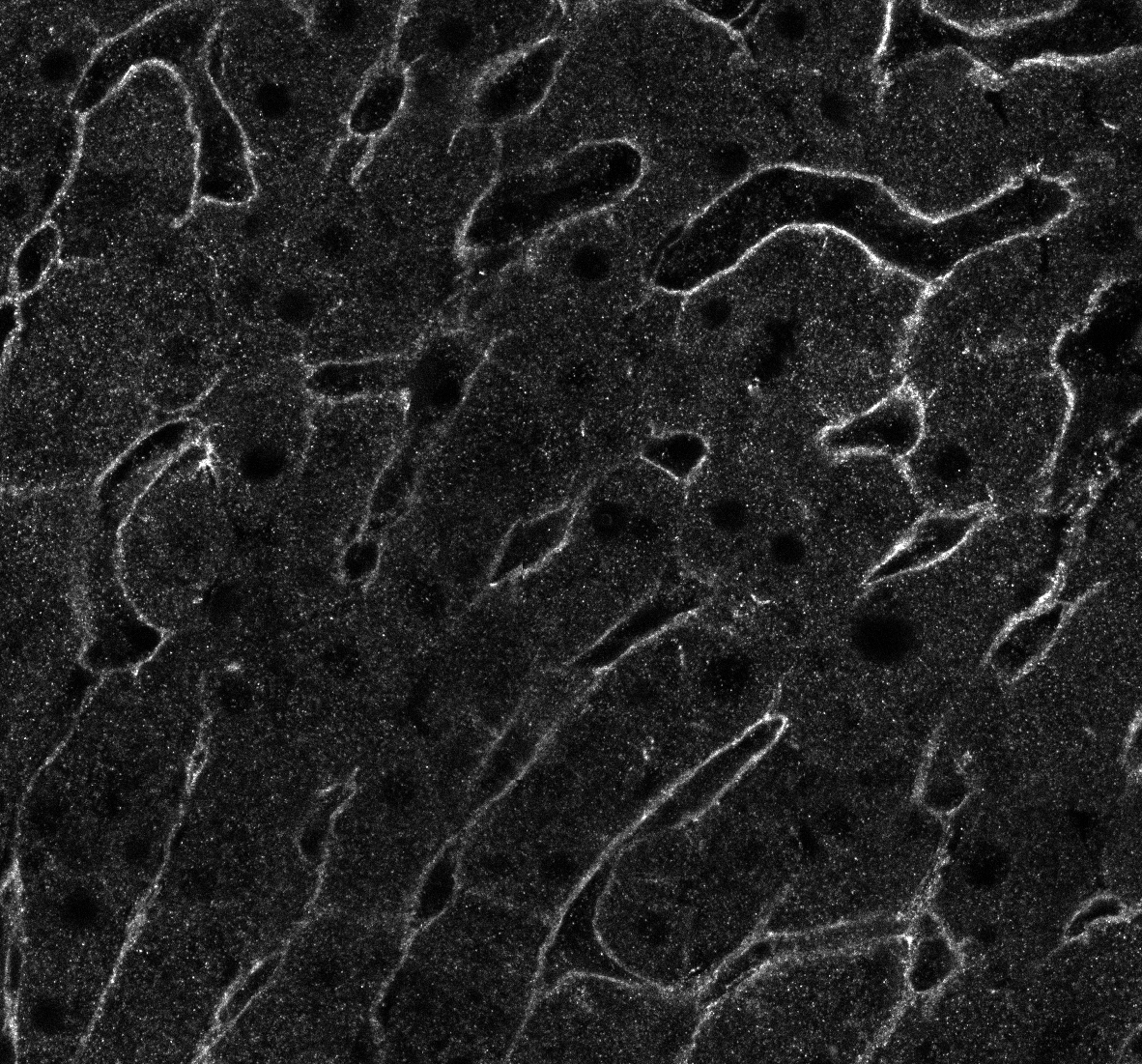

Supplement: Supplementary file 16 — Source Data for Figure 5 [file EMBR-24-e57181-s003.zip › Figure_5/5A/IF_50um_#17-2_Fibronectin_647_555_BSEP_Phall_488_DAPI_LateALD_axis_1-1.tif]
