# Supplementary material for: Apical bulkheads accumulate as adaptive response to impaired bile flow in liver disease
Source: EMBO Rep. 2023 Jul 31;24(9):e57181. doi: 10.15252/embr.202357181 (PMC10481669; doi:10.15252/embr.202357181)
Supplement: Supplementary file 17 [file EMBR-24-e57181-s001.pdf]

EMBO reports

Apical bulkheads accumulate as adaptive response to impaired bile flow in liver disease

Carlotta Mayer, Sophie Nehring, Michael Kuecken, Urska Repnik, Sarah Seifert, Aleksandra Sljukic, Julien Delpierre, Hernán Morales-Navarrete, Sebastian Hinz, Mario Brosch, Brian Chung, Tom Karlsen, Meritxell Huch, Yannis Kalaidzidis, Lutz Brusch, Jochen Hampe, Clemens Schafmayer, and Marino Zerial
DOI: 10.15252/embr.202357181

Corresponding author(s): Marino Zerial (zerial@mpi-cbg.de)

Review Timeline:

Submission Date:	13th Mar 23
Editorial Decision:	4th May 23
Revision Received:	8th Jun 23
Editorial Decision:	7th Jul 23
Revision Received:	11th Jul 23
Accepted:	13th Jul 23

Editor: Martina Rembold

Transaction Report:

(Note: With the exception of the correction of typographical or spelling errors that could be a source of ambiguity, letters and reports are not edited. Depending on transfer agreements, referee reports obtained elsewhere may or may not be included in this compilation. Referee reports are anonymous unless the Referee chooses to sign their reports.)

1st Editorial Decision 4th May 2023

Dear Dr. Zerial

Thank you for the submission of your research manuscript to our journal. We have now received the full set of referee reports that is copied below.

As you will see, referee 2 and 3 acknowledge that the findings are potentially interesting, while referee 1 raises concerns regarding the advance provided. That said, given the positive evaluation from referee 2 and 3, I would like to give you the chance to revise your study for potential publication in EMBO reports. Please address all referee concerns, except for the analysis of transciptional changes under increased pressure, which is not mandatory for publication here and might be the subject of a future study.

Please address all referee concerns in a complete point-by-point response. Acceptance of the manuscript will depend on a positive outcome of a second round of review. It is EMBO reports policy to allow a single round of revision only and acceptance or rejection of the manuscript will therefore depend on the completeness of your responses included in the next, final version of the manuscript.

We realize that it is difficult to revise to a specific deadline. In the interest of protecting the conceptual advance provided by the work, we recommend a revision within 3 months (August 4). Please discuss the revision progress ahead of this time with the editor if you require more time to complete the revisions.

I am also happy to discuss the revision further via e-mail or a video call, if you wish.

*****IMPORTANT NOTE:
We perform an initial quality control of all revised manuscripts before re-review. Your manuscript will FAIL this control and the handling will be DELAYED if the following APPLIES:

1) A data availability section providing access to data deposited in public databases is missing. If you have not deposited any data, please add a sentence to the data availability section that explains that.

2) Your manuscript contains statistics and error bars based on n=2. Please use scatter blots in these cases. No statistics should be calculated if n=2.

When submitting your revised manuscript, please carefully review the instructions that follow below. Failure to include requested items will delay the evaluation of your revision.*****

When submitting your revised manuscript, we will require:

1) a .docx formatted version of the manuscript text (including legends for main figures, EV figures and tables). Please make sure that the changes are highlighted to be clearly visible.

2) individual production quality figure files as .eps, .tif, .jpg (one file per figure).
Please download our Figure Preparation Guidelines (figure preparation pdf) from our Author Guidelines pages https://www.embopress.org/page/journal/14693178/authorguide for more info on how to prepare your figures.

3) a .docx formatted letter INCLUDING the reviewers' reports and your detailed point-by-point responses to their comments. As part of the EMBO Press transparent editorial process, the point-by-point response is part of the Review Process File (RPF), which will be published alongside your paper.

4) a complete author checklist, which you can download from our author guidelines (<https://www.embopress.org/page/journal/14693178/authorguide>). Please insert information in the checklist that is also reflected in the manuscript. The completed author checklist will also be part of the RPF.

5) Please note that all corresponding authors are required to supply an ORCID ID for their name upon submission of a revised manuscript (<https://orcid.org/>). Please find instructions on how to link your ORCID ID to your account in our manuscript tracking system in our Author guidelines
(<https://www.embopress.org/page/journal/14693178/authorguide#authorshipguidelines>)

6) We replaced Supplementary Information with Expanded View (EV) Figures and Tables that are collapsible/expandable online.

A maximum of 5 EV Figures can be typeset. EV Figures should be cited as 'Figure EV1, Figure EV2" etc... in the text and their respective legends should be included in the main text after the legends of regular figures.

- For the figures that you do NOT wish to display as Expanded View figures, they should be bundled together with their legends in a single PDF file called *Appendix*, which should start with a short Table of Content. Appendix figures should be referred to in the main text as: "Appendix Figure S1, Appendix Figure S2" etc. See detailed instructions regarding expanded view here: <https://www.embopress.org/page/journal/14693178/authorguide#expandedview>

- Additional Tables/Datasets should be labeled and referred to as Table EV1, Dataset EV1, etc. Legends have to be provided in a separate tab in case of .xls files. Alternatively, the legend can be supplied as a separate text file (README) and zipped together with the Table/Dataset file.

7) Please note that a Data Availability section at the end of Materials and Methods is now mandatory. Computer code that is publicly available should also be listed in this section, with a link that resolves to the resource.
See also < https://www.embopress.org/page/journal/14693178/authorguide#dataavailability>). Please note that the Data Availability Section is restricted to new primary data that are part of this study.

8) At EMBO Press we ask authors to provide source data for the main figures. Our source data coordinator will contact you to discuss which figure panels we would need source data for and will also provide you with helpful tips on how to upload and organize the files.

Additional information on source data and instruction on how to label the files are available <https://www.embopress.org/page/journal/14693178/authorguide#sourcedata>.

9) The journal requires a statement specifying whether or not authors have competing interests (defined as all potential or actual interests that could be perceived to influence the presentation or interpretation of an article). In case of competing interests, this must be specified in your disclosure statement. Further information: https://www.embopress.org/competing-interests

10) Figure legends and data quantification:
The following points must be specified in each figure legend:

- the name of the statistical test used to generate error bars and P values,
- the number (n) of independent experiments (please specify technical or biological replicates) underlying each data point,
- the nature of the bars and error bars (s.d., s.e.m.)

- If the data are obtained from n {less than or equal to} 5, show the individual data points in addition to the SD or SEM.
- If the data are obtained from n {less than or equal to} 2, use scatter blots showing the individual data points.

Discussion of statistical methodology can be reported in the materials and methods section, but figure legends should contain a basic description of n, P and the test applied.

See also the guidelines for figure legend preparation:
https://www.embopress.org/page/journal/14693178/authorguide#figureformat

- Please also include scale bars in all microscopy images.

11) Our journal encourages inclusion of *data citations in the reference list* to directly cite datasets that were re-used and obtained from public databases. Data citations in the article text are distinct from normal bibliographical citations and should directly link to the database records from which the data can be accessed. In the main text, data citations are formatted as follows: "Data ref: Smith et al, 2001" or "Data ref: NCBI Sequence Read Archive PRJNA342805, 2017". In the Reference list, data citations must be labeled with "[DATASET]". A data reference must provide the database name, accession number/identifiers and a resolvable link to the landing page from which the data can be accessed at the end of the reference. Further instructions are available at <https://www.embopress.org/page/journal/14693178/authorguide#referencesformat>.

12) As part of the EMBO publication's Transparent Editorial Process, EMBO reports publishes online a Review Process File to accompany accepted manuscripts. This File will be published in conjunction with your paper and will include the referee reports, your point-by-point response and all pertinent correspondence relating to the manuscript.

You are able to opt out of this by letting the editorial office know (emboreports@embo.org). If you do opt out, the Review Process File link will point to the following statement: "No Review Process File is available with this article, as the authors have chosen not to make the review process public in this case."

We would also welcome the submission of cover suggestions, or motifs to be used by our Graphics Illustrator in designing a cover.

I look forward to seeing a revised form of your manuscript when it is ready and please let me know if you have questions or comments regarding the revision.

Please use this link to submit your revision: https://embor.msubmit.net/cgi-bin/main.plex

Yours sincerely,

Martina Rembold, PhD
Senior Editor
EMBO reports

****

Referee #1:

This manuscript reports an interesting study demonstrating that excessive canalicular pressure can cause the the formation of aberrant spherical lumens and concomitant loss of apical bulkheads. The authors suggest that apical bulkheads are a protective mechanism of adult hepatocytes against impaired bile flow. The authors have previously published a related article (Bebelman et al. 2023, Hepatocyte apical bulkheads provide a mechanical means to oppose bile pressure', Journal of Cell Biology, 222: e202208002.). The advance of the current manuscript with regard to these earlier studies is that these phenomena also occur in adult liver and liver disease. In general, I find the findings in this manuscript mostly additive to their earlier studies and descriptive in nature and the underlying mechanisms that regulate the apical membrane dynamics and change in gene transcription await further studies.

Specific comments:
1. In figure 1B,D,G,H, figure 2B,C and figure 3A, figure 5 and 6: a marker for bile canalicular plasma membrane should be included to unequivocally demonstrate that the F-actin-stained structures are indeed of bile canalicular and not ductal or sinusoidal origin (which both also contain F-actin). This is important given the severe morphological alterations of bile canaliculi and formation of cyst-like lumens that may be mistaken for ducts or sinusoids.
2. BSEP staining in figure 2 (A) is unclear.
3. CD13 may not be a good bile canaliculi marker in mdr2 KO livers as it appears to have disappeared from canaliculi (Figure 1C).
4. Figure 3: the percentage of SOX9- and pan-CK-positive rosettes should be quantified.
5. Page 6: "Also in this regard, rosettes in PSC do not resemble the cholestatic liver cell rosettes described in PBC (Nagore et al. 1989) or a typical ductular reaction (Gouw, Clouston, and Theise 2011).". Please explain in what ways these are different.
6. Bile flows in the liver from the pericentral towards the periportal area (opposite blood flow). How does this relate to the difference in pericentral versus periportal canalicular pressure upon bile flow impairment (figure 4)?
7. Figure 5A: a selective bile canalicular marker instead of F-actin is preferred, as F-actin also is present at bile ducts and sinusoids (cf. figure 1A,C and figure 2C).
8. The statement on page 7 about fibrosis (aberrant ECM deposition) in end-stage PSC is of interest. It has been reported that changes in extracellular matrix composition can alter the morphogenesis and possibly the polarity organization of liver cells. Does the presence of rosettes correlate with altered ECM composition, notably presence of the otherwise in parenchyma absent laminin or collagen IV? This is not critical for the manuscript but would be a nice addition as change of ECM may also relate to change in mechanics.

Referee #2:

It was a pleasure to review the manuscript "Accumulation of apical bulkheads and hepatocyte rosettes as adaptive responses upon impaired bile flow in liver diseases" by Marino Zerial and colleagues. The authors first described apical bulkheads 2 years ago and since then expanded their studies of the apical bulkhead function as protective mechanism agains excessive canalicular pressure in a further paper in 2023 (Bebelman et al) by studying it in embryonic liver.
In the current study submitted to EMBO reports the authors further expand their analysis of the apical bulkheads now venture into the disease models both mouse (bile duct ligation and mdr2 ko) and humans (primary sclerosing cholangitis and alcoholic liver disease). They discover that with the increased pressure there is an increase in apical bulkheads especially in PSC livers. It appears that absence of apical bulkheads is associated with pseudo-canaliculi formation and hepatocyte dedifferentiation into hybrid hepatocyte-cholangiocyte cells (they call cholangiocytes "bile duct cells" for some reason).

In my opinion this is a very interesting descriptive paper which requires some minor english corrections.
I believe that the authors are in the position to provide more in depth analysis of transcriptional changes of the hepatocytes under increased pressure. This would strengthen the study greatly.

Referee #3:

In this paper, entitled "Accumulation of apical bulkheads and hepatocyte rosettes as
Accumulation of apical bulkheads and hepatocyte rosettes as adaptive responses upon impaired bile flow in liver disease", the other describes the morphological changes in bile duct morphology upon bile flow obstruction. They show that in both mice and humans, in several diseases (such as primary sclerosing cholangitis, bile duct ligation or primary biliary cholangitis), the bile canaliculi can inflate, forming a rosette-like structure that actually results from the development of larger multi-hepatocyte tubes as well as the disappearance of the bulkhead membrane folds that the authors have already described in two previous publications.
The paper reports an original and interesting study of the morphological changes attributed to the increase in pressure inside the bile canaliculi. The data are of high quality, comparing several systems in vitro and in vivo and showing the consistency of the observations. The experimental methods are quite appropriate and the conclusions are overall well supported by the statistical analysis of the data. Overall, I found the paper to be of good quality.

I would like to make some comments on how the paper could be improved and how some terms could be clarified.
1- Although I understand the logical flow that the authors have adopted in the paper, I would recommend that the studies on the bulkheads and the rosette be separated more. The paper goes back and forth between the description of both morphological features and it would be clearer for a less experienced reader in liver biology (EMBO reports) if both were dissociated. In the same vein, I felt that the abstract focused too much on the bulkhead parts, which ended up being less than half the topic of the paper.

2- The presence of BSEP at the apical pole of the dilated BC in Figure 1F is not really clear (although central to the argument).

3- The statistics (how many samples, bile canaliculi, rosettes) are not shown for Figures 2 and 3, which only show what I think are representative images.

4- A central concern of the paper is to demonstrate an increase in pressure inside the bile canaliculi in cholestatic cases and to causally relate this to the appearance of rosettes and the disappearance of bulkheads. However, the authors assume that a larger BC indicates a higher pressure. This is particularly clear for the treatments shown in Figure 7. I would like to point out here that it is not so obvious. The inflation of the BC results from the influx of water driven by an osmotic gradient created by the secretion of ions. This results in the build-up of a PHYSICAL pressure, only the peri-canalicular tension can resist the osmotic gradient. Bulk heads could certainly help. A clear example of this is the fact that BC inflate a lot when treated with blebbistatin.A simple explanation is that the cortical actomyosin network can no longer resist the osmotic gradient, which is therefore constantly compensated by an influx of water, leading to inflation with a DECREASE in physical pressure. Leaks at the edges of the BC also control these parameters. The difference between the physical pressure inside the cells and in the BC cannot exceed a few 100 Pa without causing the membrane to rupture. These effects are calculated in the following paper, which I admit can be a bit difficult to read at first glance: Dasgupta, S., K. Gupta, Y. Zhang, V. Viasnoff and J. Prost (2018). "Physics of lumen growth." Proc Natl Acad Sci U S A 115(21): E4751-E4757. All in all, I do not think that the authors should completely revise their conclusions, but at least tame their claim and reconsider the possibility of other conclusions based on this fact.
5- The sentence: "Our analysis sheds light on the molecular mechanism underlying the appearance of liver cell rosettes in PSC and possibly other liver diseases". is a bit exaggerated in the Discussion section. I am not quite sure what molecular mechanism has been revealed. This other sentence in the same paragraph: "Second, we provide the first that these pressure-induced changes in the hepatocyte apical membrane can affect gene transcription, with induction of bile duct cell markers in some of the hepatocytes in the rosettes" is also exaggerated. I would just say that you show a correlation that suggests...

Minor comments:
1- The word structure is spelt stricture several times in the paper.
2- In the legend of figure 1, canaliculi is spelled cancanliculi.
3- In the paragraph Liver cell patients do not... the reference to Figure 2E seems to be incorrect.
4- In the paragraph Liver cell patients do not... the reference to Figure 2E seems to be incorrect.
5- The values of R2 in the correlation in Figure 4M are quite small. It might be better to tame the text describing this panel/result.

Martina Rembold, PhD
Senior Editor
EMBO reports

Dresden, 8th June 2023

Dear Martina,

Thank you for your editorial response to our manuscript and your encouragement to proceed with the revision for potential publication in EMBO reports.

We have conducted new stainings to unambiguously proof the identity of bile canaliculi in multiple figures. Further we performed several new quantifications and modified the text to increase the clarity.

Below please find our detailed answers to the Referees' comments.

Referee #1:
1. In figure 1B,D,G,H, figure 2B,C and figure 3A, figure 5 and 6: a marker for bile canalicular plasma membrane should be included to unequivocally demonstrate that the F-actin-stained structures are indeed of bile canalicular and not ductal or sinusoidal origin (which both also contain F-actin). This is important given the severe morphological alterations of bile canaliculi and formation of cyst-like lumens that may be mistaken for ducts or sinusoids.
Figure 1B,D,G,H: As requested we added the staining of CD13 as canalicular membrane marker in figure 1B,D, H,. For better visibility of the apical bulkheads in the main figure we decided to keep only the F-Actin staining there. Additional single channel panels of CD13 and F-Actin are now in figure EV1 B and D.
Figure 1G shows ZO-1 in magenta, which is a tight junction marker, which seal the bile canaliculi and, hence, is a canalicular marker itself. Therefore, we did not add any further marker. However, we added the single channel panels into EV1 E.
Figure 2B,C: We added the staining for BSEP, another canalicular membrane marker in figure EV2 B (together with F-Actin) and figure 2C.
Figure 3A: It was transferred to figure 3E to avoid confusion with a non-representative image for the main phenotype described (which is quantified, see Rev. 3). Unfortunately, both (Sox9 and canalicular marker BSEP) antibodies are rabbit and, thus, incompatible for co-staining. To address the referee's concern, we added the maximum projection of a full Z stack a) to show a more zoomed out view and appreciate that there is no portal vein close, reducing the likelihood of the rosette being a bile duct and b) to show that the rosette is directly connected to the bile canalicular network (highlighted with a red arrowhead) and, thus, excluding that they are sinusoids. Also, the F-Actin intensity at the bile canaliculi is much higher than at the sinusoids, which also helps to differentiate them.
Figure 5A: We added a panel with Fibronectin staining, a marker for sinusoids, to show that the lumina of the rosettes are on opposite sides of the sinusoids (marked by red arrowheads). This shows that the rosettes are of canalicular origin.
Figure 6A,B: We added CD13 as bile canalicular marker and additionally put the individual channels into figure EV3 A. For figure 6B, we added the colocalization with CD13 into figure EV3 B. We did not implement it into the main figure because it makes it harder to see the apical bulkheads.
Figure 6J: We added ZO-1 as additional canalicular marker into expanded view 3I.
Figure 7B: In line with the prior suggestions we also added CD13 as canalicular marker in figure 7B.

BSEP staining in figure 2 (A) is unclear.
We increased the brightness in the main figures and added panels of individual channels to better visualize the BSEP staining in EV 2A.

CD13 may not be a good bile canaliculi marker in mdr2 KO livers as it appears to have disappeared from canaliculi (Figure 1C).
Indeed, the intensity of CD13 appears reduced upon BDL and MDR2 KO, but it is not absent. The lower signal may be simply due the larger surface area of the canaliculi, as they are very dilated. To better visualize the structures, we increased the brightness in the main figures 1A and 1C. Additionally we added the single channel panels into EV1 A and C.

Figure 3: the percentage of SOX9- and pan-CK-positive rosettes should be quantified.
We added the quantification of SOX9 and pan-CK-positive rosettes in figure 3F and G.

Page 6: "Also in this regard, rosettes in PSC do not resemble the cholestatic liver cell rosettes described in PBC (Nagore et al. 1989) or a typical ductular reaction (Gouw, Clouston, and Theise 2011).". Please explain in what ways these are different.
To further clarify the differences between rosettes in different disease, we edited the text: “This is different from the cholestatic liver cell rosettes described in PBC (Nagore et al. 1989) or a typical ductular reaction (Gouw, Clouston, and Theise 2011), which are directly connected to the bile ducts.” This point was also addressed in the discussion in more detail (page 9).

Bile flows in the liver from the pericentral towards the periportal area (opposite blood flow). How does this relate to the difference in pericentral versus periportal canalicular pressure upon bile flow impairment (figure 4)?
We thank the referee for this consideration and question. Flow from pericentral to periportal areas requires a pressure gradient, i.e. decreasing canalicular pressure from pericentral to periportal areas. This gradient appears in the model predictions for PSC samples and hence is correctly captured by the model. This is the same in normal and disease conditions.
The model currently assumes a similar bile load for control and PSC patients. Due to the overall reduction of the bile canaliculi network in PSC patients this results in a higher flow speed (similar fluid volume gets pressed with higher pressure through narrower channels and that would result in higher flow speed). Thus, the median elevation of pericentral over periportal pressure is predicted to be higher in PSC patients.
However, if there would be a reduction in bile load or a significant change in speed inside the bile ducts, elevation of pericentral over periportal pressure would be affected. So far, we kept bile load parameter (and other parameters like membrane permeability and bile composition, bile viscosity) unchanged as we focus on the effects of perturbation of the bile canaliculi network geometry.
In response to the referee, we modified the text in order to clarify this point (page 6).

Figure 5A: a selective bile canalicular marker instead of F-actin is preferred, as F-actin also is present at bile ducts and sinusoids (cf. figure 1A,C and figure 2C).
We added a panel with Fibronectin staining, a marker for sinusoids, to show that the lumina of the rosettes are on opposite sides of the sinusoids (marked by red arrowheads). This shows that the rosettes are of canalicular origin.

The statement on page 7 about fibrosis (aberrant ECM deposition) in end-stage PSC is of interest. It has been reported that changes in extracellular matrix composition can alter the morphogenesis and possibly the polarity organization of liver cells. Does the presence of rosettes correlate with altered ECM composition, notably presence of the otherwise in parenchyma absent laminin or collagen IV? This is not critical for the manuscript but would be a nice addition as change of ECM may also relate to change in mechanics.
We thank the referee for this interesting consideration. Although we did not conduct a comprehensive analysis of ECM composition with respect to rosettes in PSC, we mentioned in the discussion that the deposition of ECM

could affect the polarization of hepatocytes and the morphology of the bile canaliculi network, and thus that ECM should be inspected in future analysis of cell polarity and rosette formation (p. 10).

Referee #2:

I believe that the authors are in the position to provide more in depth analysis of transcriptional changes of the hepatocytes under increased pressure. This would strengthen the study greatly.

We very much agree with the referee's suggestion about the transcriptional changes under increased pressure. To do so, it is essential to differentiate pressure-driven effects from bile acid signalling and, thus, only looking at the transcriptional changes upon DCA treatment is not sufficient. We are currently developing another approach to achieve this in order to enable such transcriptomics analysis.

Referee #3:

Although I understand the logical flow that the authors have adopted in the paper, I would recommend that the studies on the bulkheads and the rosette be separated more. The paper goes back and forth between the description of both morphological features and it would be clearer for a less experienced reader in liver biology (EMBO reports) if both were dissociated. In the same vein, I felt that the abstract focused too much on the bulkhead parts, which ended up being less than half the topic of the paper.

There may be a misunderstanding, in the sense that we do not wish to separate the bulkheads and rosette parts of the study. Quite on the contrary, we spent effort to explain that apical bulkheads accumulation and formation of rosettes in PSC are not separate phenomena but linked mechanistically as evolution of the cellular and tissue response to different degrees of luminal (bile canaliculi) pressure. We further edited the abstract, introduction (p. 4), results (p.5, 7, 8) and discussion (p.9) to harmonize better the two parts and make this point clearer. We acknowledge of course the fact that the EMBO report readership is primarily a basic cell biology community. We would view the approach to study a cell biology mechanism in the context of a physiological system (liver tissue analysed in 3D) and a disease model (PSC) a point of strength that should indeed capture the interests of both the cell biology as well as liver physiology/pathology communities.

The presence of BSEP at the apical pole of the dilated BC in Figure 1F is not really clear (although central to the argument).

We believe the referee is referring to figure 2F. To make the presence of BSEP at the apical pole more visible we added the BSEP staining additionally as individual panel.

The statistics (how many samples, bile canaliculi, rosettes) are not shown for Figures 2 and 3, which only show what I think are representative images.

For figures 2 and 3 we qualitatively analysed the same samples that were later fully quantified in Figure 4. In total we analysed for that N=3 control patients and N=4 PSC patients. We added the number of analysed samples into the figure legend.

Upon request of referee #1 we additionally quantified the number of Sox9- and pan-CK positive rosettes in figure 3.

A central concern of the paper is to demonstrate an increase in pressure inside the bile canaliculi in cholestatic cases and to causally relate this to the appearance of rosettes and the disappearance of bulkheads. However, the authors assume that a larger BC indicates a higher pressure. This is particularly clear for the treatments shown in Figure 7. I would like to point out here that it is not so obvious. The inflation of the BC results from the influx of water driven by an osmotic gradient created by the secretion of ions. This results in the build-up of a PHYSICAL pressure, only the peri-canalicular tension can resist the osmotic gradient. Bulk heads could certainly help. A clear example of this is the fact that BC inflate a lot when treated with blebbistatin.A simple explanation is that the cortical actomyosin network can no longer resist the osmotic gradient, which is therefore constantly

compensated by an influx of water, leading to inflation with a DECREASE in physical pressure. Leaks at the edges of the BC also control these parameters. The difference between the physical pressure inside the cells and in the BC cannot exceed a few 100 Pa without causing the membrane to rupture. These effects are calculated in the following paper, which I admit can be a bit difficult to read at first glance: Dasgupta, S., K. Gupta, Y. Zhang, V. Viasnoff and J. Prost (2018). "Physics of lumen growth." Proc Natl Acad Sci U S A 115(21): E4751-E4757. All in all, I do not think that the authors should completely revise their conclusions, but at least tame their claim and reconsider the possibility of other conclusions based on this fact.

We would like to thank the referee for this comment that made us realize that our explanation for the mentioned conclusions were insufficient. Therefore, we added also further explanations into the main text (p. 9) and the discussion (p. 10).

Lumina are balanced by hydrostatic pressure and the actomyosin cortex strength. The hydrostatic pressure is built by water influx into the lumen due to an osmotic gradient, which is maintained by osmolyte transporters. The actomyosin cortex does not resist the osmotic gradient (because it is not a physical pressure), it can only resist the hydrostatic pressure. Only if the hydrostatic pressure exceeds the actomyosin cortex tension, dilation occurs. Thus, dilation is caused either by weakening of the actomyosin cortex (e.g. Blebbistatin treatment) or by increase in hydrostatic pressure. Other compensating mechanisms like bile leakage, bile regurgitation or altered bile transporters do play a role, but if dilation occurs the hydrostatic pressure nevertheless has exceeded the actomyosin cortex strength.

Cholestasis is characterized by an accumulation of bile that directly increases the osmotic gradient and thus the hydrostatic pressure. It is well-established that in cholestasis the canalicular actomyosin cortex is strengthened (Gupta et al. 2017) and also DCA treatment raises phosphorylation of myosin light chain levels *in-vitro* (Meyer et al. 2020). This further supports the fact that dilation of bile canaliculi under cholestatic conditions is directly associated with an increase in hydrostatic pressure and balancing actomyosin tension. In the experiment of figure 7B inhibiting apical water influx in the presence of DCA prevents dilation because the increase of hydrostatic pressure is blocked although the osmotic gradient is still present. Also, it is important to note that the hydrostatic pressure does not decrease after dilation, otherwise the bile canaliculus would shrink back.

To verify that canalicular dilation does not only occur upon DCA treatment but also with other bile acids we performed additional experiments with the sodium-conjugated bile acid taurocholic acid (TCA). The results confirm that also treatment of primary hepatocytes with 50 µm TCA leads to canalicular dilation and changes the expression of the known cholestasis-associated genes Bsep and Cyp7. The results are in expanded view 3 D-H.

Additionally, we want to comment on the opinion that pressures of >100 Pa lead to membrane rupture. It is important to note that the pressure is not kept by the cell membrane but by the underlying actomyosin cortex which sustains much higher pressures than the membrane alone. For example, the sinusoidal blood pressure in the liver is between 150-700 Pascal and much higher pressures can be observed in case of portal hypertension (De Franchis et al. 2022). Although bile canalicular pressure cannot be measured directly, there are multiple manometric studies in the common bile duct of human and muse liver. Since the bile canaliculi network has no insulation (like valves or sphincters), the pressure inside the bile canaliculi has to be equal or higher than the pressure inside the common bile duct. These studies provided direct experimental evidence of a baseline common bile duct pressure in dogs, cats and rabbits between 2000-3000 Pa (Herring and Simpson 1907) and in humans between 500-1500 Pa (Beltrán and Beltrán 2021; Wong, Frey, and Gagic 1980; Csendes et al. 1979). In patients with gallstones, this pressure is elevated in the range of 2000 Pa but can reach also values of 5000 Pa. In order to really clarify this point also for the general EMBO Reports readership, we added this aspect to the main text (p. 6) and thank again the referee for raising this important issue.

The sentence: "Our analysis sheds light on the molecular mechanism underlying the appearance of liver cell rosettes in PSC and possibly other liver diseases". is a bit exaggerated in the Discussion section. I am not quite

sure what molecular mechanism has been revealed. This other sentence in the same paragraph: "Second, we provide the first that these pressure-induced changes in the hepatocyte apical membrane can affect gene transcription, with induction of bile duct cell markers in some of the hepatocytes in the rosettes" is also exaggerated. I would just say that you show a correlation that suggests...
We agree with the referees comment and modified the indicated sentences accordingly (p. 10)

Minor comments:
The word structure is spelt stricture several times in the paper.
In this context, the word “strictures” is correct. PSC is a disease is characterized by strictures and dilatations in the bile ducts (Karlsen et al. 2017).

In the legend of figure 1, canaliculi is spelled cancanliculi.
Corrected

In the paragraph Liver cell patients do not... the reference to Figure 2E seems to be incorrect.
Corrected

In the paragraph Liver cell patients do not... the reference to Figure 2E seems to be incorrect.
Corrected

The values of $R2$ in the correlation in Figure 4M are quite small. It might be better to tame the text describing this panel/result.
We thank the referee for this important comment. We decided that linear regression analysis is not the right analysis because all parameters are not certain. Thus, a Pearson’s correlation analysis is more suitable. We corrected the results in the text (p.7). In light of these correlation values, we don’t think that our conclusion that “[our results] suggest that with increasing canalicular pressure the liver cell rosettes occupy more volume of the canalicular network and reduce the overall network connectivity.” is overstated.

Having revised the manuscript according to the suggestions, we hope that it can be accepted for publication in EMBO reports.

Thanking you for your consideration, I look forward to hearing from you.

Yours sincerely

Marino

Dear Dr. Zerial

Thank you for the submission of your revised manuscript to EMBO reports. We have now received the full set of referee reports that is copied below.

As you can see, the referees find that the revision addressed all their concerns and recommend publication. Before I can accept the manuscript, I need you to address some editorial points below:

- Please provide up to 5 keywords.

- Please update the 'Conflict of interest' paragraph to our new 'Disclosure and competing interests statement'. For more information see
https://www.embopress.org/page/journal/14693178/authorguide#conflictsofinterest

- Please remove the Author Contributions from the manuscript and make sure that the author contributions in our online submission system are correct and up-to-date. The information you specified in the system will be automatically retrieved and typeset into the article. You can enter additional information in the free text box provided, if you wish.

- References: Please only list the first 10 authors followed by et al.
Please update the format to match our style. The first reference would e.g., be:
Nadim A, Saviano A, Laurent Mailly S, Durand S, Herman JS, Pessaux P, Baumert TF, Grün D (2019) A human liver cell atlas reveals heterogeneity and epithelial progenitors', Nature 572 [Bold]: 199-204.

- The "Financial support statement" should be included in the "Acknowledgements".

- Figure callouts to Expanded View Figures should be 'Figure EV#'.

- Please add callouts to Fig. EV3A

- Figure panels should be called out in an alphabetical order. If possible, please rearrange Fig. 3 and EV3.

- Supplementary methods: It seems that the file reports the Fiji script used for image segmentation. I think it is fine to provide it as Supplementary file but please call it Appendix and add a title page with a table of content (incl page numbers). I also recommend adding some description or legend indicating that this is the Fiji script used for ..."

- Movies should be renamed to Movie EV# with the corresponding callouts in the text. The legends should be removed from the manuscript file, provided as a simple README.txt file and zipped with each movie file.

- The final size of the synopsis image will be 550 pixels width. At this size the text describing e.g. 'Bile canaliculi' is rather small. You might want to increase text size.

- Please provide a draft for the synopsis text that will accompany the synopsis image. We need (A) 1 - 2 sentences summarizing the findings and their significance and (B) 3 - 4 bullet points highlighting key results.

- It seems that some images shown in Fig. EV4A are the same as those in Fig. 7B but represent a larger view. To avoid any ambiguities, please state the re-use in the figure legends.

- Data availability section: Please add the link to the code at github.com there as well.

- It seems that you had uploaded 2 files for Fig 6, one of which seemed to be Fig. 7. We have corrected this for you, but please double-check these files.

- Structured Methods: Please either use our template for the Reagents and tools table (see https://www.embopress.org/page/journal/14693178/authorguide and look for Structure Methods) or upload the tables as separate Table 1-3 or Expanded View table (Table EV1-3). In the latter case, you need to change 'Methods and Protocols' to 'Material and Methods'.

- I attach to this email a related manuscript file with comments by our data editors. Please address all comments and upload a revised file with tracked changes with your final manuscript submission.

I look forward to seeing a new revised version of your manuscript as soon as possible. Please use this link to submit your revision: https://embor.msubmit.net/cgi-bin/main.plex

Kind regards,

Martina

Martina Rembold, PhD
Senior Editor
EMBO reports

****

Referee #1:

All comments I raised have been addressed satisfactorily.

Referee #3:

I congratulate the authors for their additional changes and experiments that nicely strengthen their conclusions.

The authors have addressed all minor editorial requests.’

Marino Zerial
Max Planck Institute of Molecular Cell Biology and Genetics
-
Pfotenhauerstr. 108
Dresden 01307
Germany

Dear Marino,

I am very pleased to accept your manuscript for publication in the next available issue of EMBO reports. Thank you for your contribution to our journal.

At the end of this email I include important information about how to proceed. Please ensure that you take the time to read the information and complete and return the necessary forms to allow us to publish your manuscript as quickly as possible.

As part of the EMBO publication's Transparent Editorial Process, EMBO reports publishes online a Review Process File to accompany accepted manuscripts. As you are aware, this File will be published in conjunction with your paper and will include the referee reports, your point-by-point response and all pertinent correspondence relating to the manuscript.

If you do NOT want this File to be published, please inform the editorial office within 2 days, if you have not done so already, otherwise the File will be published by default [contact: emboreports@embo.org]. If you do opt out, the Review Process File link will point to the following statement: "No Review Process File is available with this article, as the authors have chosen not to make the review process public in this case."

Thank you again for your contribution to EMBO reports and congratulations on a successful publication. Please consider us again in the future for your most exciting work.

Kind regards,

Martina

Martina Rembold, PhD
Senior Editor
EMBO reports

****

THINGS TO DO NOW:

Please note that you will be contacted by Wiley Author Services to complete licensing and payment information. The required 'Page Charges Authorization Form' is available here: https://www.embopress.org/pb-assets/embo-site/er_apc.pdf - please download and complete the form and return to embopressproduction@wiley.com

EMBO Press participates in many Publish and Read agreements that allow authors to publish Open Access with reduced/no publication charges. Check your eligibility: https://authorservices.wiley.com/author-resources/Journal-Authors/open-access/affiliation-policies-payments/index.html

You will receive proofs by e-mail approximately 2-3 weeks after all relevant files have been sent to our Production Office; you should return your corrections within 2 days of receiving the proofs.

Please inform us if there is likely to be any difficulty in reaching you at the above address at that time. Failure to meet our deadlines may result in a delay of publication, or publication without your corrections.

All further communications concerning your paper should quote reference number EMBOR-2023-57181V3 and be addressed to emboreports@wiley.com.

Should you be planning a Press Release on your article, please get in contact with emboreports@wiley.com as early as possible, in order to coordinate publication and release dates.

EMBO Press Author Checklist

Corresponding Author Name: Marino Zerial	
Journal Submitted to: EMBO Reports	
Manuscript Number: EMBOR-2023-57181-T	

USEFUL LINKS FOR COMPLETING THIS FORM
[The EMBO Journal - Author Guidelines]
[EMBO Reports - Author Guidelines]
[Molecular Systems Biology - Author Guidelines]
[EMBO Molecular Medicine - Author Guidelines]

Reporting Checklist for Life Science Articles (updated January 2022)
This checklist is adapted from Materials Design Analysis Reporting (MDAR) Checklist for Authors. MDAR establishes a minimum set of requirements in transparent reporting in the life sciences (see Statement of Task: 10.31222/osf.io/9sm4x). Please follow the journal's guidelines in preparing your manuscript.
Please note that a copy of this checklist will be published alongside your article.

Abridged guidelines for figures
1. Data
The data shown in figures should satisfy the following conditions:
- the data were obtained and processed according to the field's best practice and are presented to reflect the results of the experiments in an accurate and unbiased manner.
- ideally, figure panels should include only measurements that are directly comparable to each other and obtained with the same assay.
- plots include clearly labeled error bars for independent experiments and sample sizes. Unless justified, error bars should not be shown for technical replicates.
- if n<5, the individual data points from each experiment should be plotted. Any statistical test employed should be justified.
- Source Data should be included to report the data underlying figures according to the guidelines set out in the authorship guidelines on Data Presentation.

2. Captions
Each figure caption should contain the following information, for each panel where they are relevant:
- a specification of the experimental system investigated (eg cell line, species name).
- the assay(s) and method(s) used to carry out the reported observations and measurements.
- an explicit mention of the biological and chemical entity(ies) that are being measured.
- an explicit mention of the biological and chemical entity(ies) that are altered/varied/perturbed in a controlled manner.
- the exact sample size (n) for each experimental group/condition, given as a number, not a range;
- a description of the sample collection allowing the reader to understand whether the samples represent technical or biological replicates (including how many animals, litters, cultures, etc.).
- a statement of how many times the experiment shown was independently replicated in the laboratory.
- definitions of statistical methods and measures:
  - common tests, such as t-test (please specify whether paired vs. unpaired), simple χ2 tests, Wilcoxon and Mann-Whitney tests, can be unambiguously identified by name only, but more complex techniques should be described in the methods section;
  - are tests one-sided or two-sided?
  - are there adjustments for multiple comparisons?
  - exact statistical test results, e.g., P values = x but not P values < x;
  - definition of 'center values' as median or average;
  - definition of error bars as s.d. or s.e.m.

Please complete ALL of the questions below.
Select "Not Applicable" only when the requested information is not relevant for your study.

Materials

Newly Created Materials	Information included in the manuscript?	In which section is the information available? (Reagents and Tools Table, Materials and Methods, Figures, Data Availability Section)
New materials and reagents need to be available; do any restrictions apply?	Not Applicable	

Antibodies	Information included in the manuscript?	In which section is the information available? (Reagents and Tools Table, Materials and Methods, Figures, Data Availability Section)
For **antibodies** provide the following information:<br>- Commercial antibodies: RRID (if possible) or supplier name, catalogue number and or/clone number<br>- Non-commercial: RRID or citation	Yes	Materials and Methods, Table 2 and 3

DNA and RNA sequences	Information included in the manuscript?	In which section is the information available? (Reagents and Tools Table, Materials and Methods, Figures, Data Availability Section)
**Short novel DNA or RNA including primers, probes**: provide the sequences.	Yes	Materials and Methods, Table 1

Cell materials	Information included in the manuscript?	In which section is the information available? (Reagents and Tools Table, Materials and Methods, Figures, Data Availability Section)
**Cell lines:** Provide species information, strain. Provide accession number in repository **OR** supplier name, catalog number, clone number, and/**OR** RRID.	Not Applicable	
**Primary cultures:** Provide species, strain, sex of origin, genetic modification status.	Yes	Materials and Methods
Report if the cell lines were recently **authenticated** (e.g., by STR profiling) and tested for mycoplasma contamination.	Not Applicable	

Experimental animals	Information included in the manuscript?	In which section is the information available? (Reagents and Tools Table, Materials and Methods, Figures, Data Availability Section)
**Laboratory animals or Model organisms:** Provide species, strain, sex, age, genetic modification status. Provide accession number in repository **OR** supplier name, catalog number, clone number, **OR** RRID.	Yes	Materials and Methods, Animals
**Animal observed in or captured from the field:** Provide species, sex, and age where possible.	Not Applicable	
Please detail **housing and husbandry conditions**.	Yes	Materials and Methods, Animals

Plants and microbes	Information included in the manuscript?	In which section is the information available? (Reagents and Tools Table, Materials and Methods, Figures, Data Availability Section)
**Plants:** provide species and strain, ecotype and cultivar where relevant, unique accession number if available, and source (including location for collected wild specimens).	Not Applicable	
**Microbes:** provide species and strain, unique accession number if available, and source.	Not Applicable	

Human research participants	Information included in the manuscript?	In which section is the information available? (Reagents and Tools Table, Materials and Methods, Figures, Data Availability Section)
If collected and within the bounds of privacy constraints report on age, sex and gender or ethnicity for all study participants.	Yes	Materials and Methods, Human liver samples

Core facilities	Information included in the manuscript?	In which section is the information available? (Reagents and Tools Table, Materials and Methods, Figures, Data Availability Section)
If your work benefited from core facilities, was their service mentioned in the acknowledgments section?	Yes	Acknowledgement

Design

Study protocol	Information included in the manuscript?	In which section is the information available? (Reagents and Tools Table, Materials and Methods, Figures, Data Availability Section)
If study protocol has been **pre-registered, provide DOI in the manuscript**. For clinical trials, provide the trial registration number **OR** cite DOI.	Not Applicable	
Report the **clinical trial registration number** (at ClinicalTrials.gov or equivalent), where applicable.	Not Applicable	

Laboratory protocol	Information included in the manuscript?	In which section is the information available? (Reagents and Tools Table, Materials and Methods, Figures, Data Availability Section)
Provide DOI OR other citation details if **external detailed step-by-step protocols** are available.	Not Applicable	

Experimental study design and statistics	Information included in the manuscript?	In which section is the information available? (Reagents and Tools Table, Materials and Methods, Figures, Data Availability Section)
Include a statement about **sample size** estimate even if no statistical methods were used.	Yes	Figure legends
Were any steps taken to minimize the effects of subjective bias when allocating animals/samples to treatment (e.g. **randomization procedure**)? If yes, have they been described?	Yes	Materials and Methods
Include a statement about **blinding** even if no blinding was done.	Yes	Materials and Methods
Describe **inclusion/exclusion criteria** if samples or animals were excluded from the analysis. Were the criteria pre-established?<br><br>If sample or data points were omitted from analysis, report if this was due to attrition or intentional exclusion and provide justification.	Not Applicable	
For every figure, are **statistical tests** justified as appropriate? Do the data meet the assumptions of the tests (e.g., normal distribution)? Describe any methods used to assess it. Is there an estimate of variation within each group of data? Is the variance similar between the groups that are being statistically compared?	Yes	Figure legends and main text

Sample definition and in-laboratory replication	Information included in the manuscript?	In which section is the information available? (Reagents and Tools Table, Materials and Methods, Figures, Data Availability Section)
In the figure legends: state number of times the experiment was **replicated** in laboratory.	Yes	Figure legends
In the figure legends: define whether data describe **technical or biological replicates**.	Yes	Figure legends

Ethics

Ethics	Information included in the manuscript?	In which section is the information available? (Reagents and Tools Table, Materials and Methods, Figures, Data Availability Section)
Studies involving **human participants**: State details of **authority granting ethics approval** (IRB or equivalent committee(s), provide reference number for approval.	Yes	Materials and Methods, Human liver samples
Studies involving **human participants**: Include a statement confirming that **informed consent** was obtained from all subjects and that the experiments conformed to the principles set out in the WMA Declaration of Helsinki and the Department of Health and Human Services Belmont Report.	Yes	Materials and Methods, Human liver samples
Studies involving **human participants:** For publication of **patient photos**, include a statement confirming that consent to publish was obtained.	Not Applicable	
Studies involving experimental **animals**: State details of **authority granting ethics approval** (IRB or equivalent committee(s), provide reference number for approval. Include a statement of compliance with ethical regulations.	Yes	Materials and Methods
Studies involving **specimen and field samples:** State if relevant **permits** obtained, provide details of authority approving study; if none were required, explain why.	Not Applicable	

Dual Use Research of Concern (DURC)	Information included in the manuscript?	In which section is the information available? (Reagents and Tools Table, Materials and Methods, Figures, Data Availability Section)
Could your study fall under dual use research restrictions? Please check biosecurity documents and list of **select agents and toxins** (CDC): https://www.selectagents.gov/sat/list.htm	Not Applicable	
If you used a select agent, is the security level of the lab appropriate and reported in the manuscript?	Not Applicable	
If a study is subject to dual use research of concern regulations, is the name of the **authority granting approval and reference number** for the regulatory approval provided in the manuscript?	Not Applicable	

Reporting

The MDAR framework recommends adoption of discipline-specific guidelines, established and endorsed through community initiatives. Journals have their own policy about requiring specific guidelines and recommendations to complement MDAR.

Adherence to community standards	Information included in the manuscript?	In which section is the information available? (Reagents and Tools Table, Materials and Methods, Figures, Data Availability Section)
State if relevant guidelines or checklists (e.g., **ICMJE, MIBBI, ARRIVE, PRISMA**) have been followed or provided.	Yes	
For **tumor marker prognostic studies**, we recommend that you follow the **REMARK** reporting guidelines (see link list at top right). See author guidelines, under 'Reporting Guidelines'. Please confirm you have followed these guidelines.	Not Applicable	
For **phase II and III randomized controlled trials**, please refer to the **CONSORT** flow diagram (see link list at top right) and submit the CONSORT checklist (see link list at top right) with your submission. See author guidelines, under 'Reporting Guidelines'. Please confirm you have submitted this list.	Not Applicable	

Data Availability

Data availability	Information included in the manuscript?	In which section is the information available? (Reagents and Tools Table, Materials and Methods, Figures, Data Availability Section)
Have **primary datasets** been deposited according to the journal's guidelines (see 'Data Deposition' section) and the respective accession numbers provided in the Data Availability Section?	Not Applicable	
Were **human clinical and genomic datasets** deposited in a public access-controlled repository in accordance to ethical obligations to the patients and to the applicable consent agreement?	Not Applicable	
Are **computational models** that are central and integral to a study available without restrictions in a machine-readable form? Were the relevant accession numbers or links provided?	Yes	Materials and Methods, Canalicular bile pressure model
If publicly available data were reused, provide the respective **data citations in the reference list**.	Not Applicable	
